# Common Modality Effects in Immediate Free Recall and Immediate Serial Recall

**DOI:** 10.1037/xlm0000430

**Published:** 2017-05-29

**Authors:** Rachel Grenfell-Essam, Geoff Ward, Lydia Tan

**Affiliations:** 1 School of Sport, Exercise and Health Sciences, Loughborough University; 2 Department of Psychology, University of Essex; 3 Department of Psychology, City, University of London

**Keywords:** free recall, serial recall, modality effects, inverse modality effects, output interference

## Abstract

In 2 experiments, participants were presented with lists of between 2 and 12 words for either immediate free recall (IFR) or immediate serial recall (ISR). Auditory recall advantages at the end of the list (modality effects) and visual recall advantages early in the list (inverse modality effects) were observed in both tasks and the extent and magnitude of these effects were dependent upon list length. Both tasks displayed modality effects with short lists that were large in magnitude but limited to the final serial position, consistent with those observed in the typically short lists used in ISR, and both tasks displayed modality effects with longer lists that were small in magnitude and more extended across multiple end-of-list positions, consistent with those observed in the typically longer lists used in IFR. Inverse modality effects were also observed in both tasks at early list positions on longer lengths. Presentation modality did not affect where recall was initiated, but modality effects were greatest on trials where participants initiated recall with the first item. We argue for a unified account of IFR and ISR. We also assume that the presentation modality affects the encoding of all list items, and that modality effects emerge due to the greater resistance of auditory items to output interference.

The purpose of this research is twofold. First, we seek to examine the similarities and differences between two highly influential and widely used immediate memory tasks, immediate serial recall (ISR) and immediate free recall (IFR), with a view to encourage greater theoretical integration between the two literatures. Second, we seek to examine the characteristics and cause of the *modality effect*, the recall advantage for words presented at the end of the list that are spoken or read aloud over words that are read silently, in order to determine whether the modality effects that are observed in ISR are underpinned by the same mechanisms as the modality effects that are observed in IFR.

Let us first consider the basic methodology of IFR and ISR. In both tasks, participants are presented with a sequence of items, one at a time, and at the end of the list participants are required to recall as many items as they can, either in the same order as that presented (ISR) or in any order they wish (IFR). The two tasks typically differ in the number of words that are presented in a sequence. ISR is usually studied using short sequences of around five to eight items. A list item in ISR is conventionally scored as correct only if it is output in the same serial position as it was presented in (*SR scoring*), and performance is dominated by primacy effects (the recall advantage for early list items, e.g., Drewnowski & Murdock, 1980). By contrast, IFR is usually studied using far longer sequences of around 10 to 40 items. A list item is scored as correct regardless of its output position (*FR scoring*), and performance is dominated by extended recency effects (the recall advantage for later list items, e.g., Murdock, 1962).

As reviewed by Ward, Tan, and Grenfell-Essam (2010), until recently, the data from the two tasks were largely explained by different sets of theories. Theories of ISR tended to focus on explaining primacy effects, and said relatively little about performance in IFR (e.g., Anderson & Matessa, 1997; Baddeley, 1986, 2007, 2012; Botvinick & Plaut, 2006; Brown, Preece, & Hulme, 2000; Burgess & Hitch, 1999, 2006; Farrell & Lewandowsky, 2002; Henson, 1998; Lee & Estes, 1981; Lewandowsky & Farrell, 2008; Lewandowsky & Murdock, 1989; Nairne, 1988, 1990; Neath, 2000; Oberauer & Lewandowsky, 2008; Page & Norris, 1998, 2003). By contrast, theories of IFR tended to focus on explaining recency effects and said relatively little about performance in ISR (e.g., Davelaar, Goshen-Gottstein, Ashkenazi, Haarmann, & Usher, 2005; Farrell, 2010; Howard & Kahana, 2002; Laming, 2006, 2010; Lehman & Malmberg, 2013; Metcalfe & Murdock, 1981; Polyn, Norman, & Kahana, 2009; Raaijmakers & Shiffrin, 1981; Sederberg, Howard, & Kahana, 2008; Tan & Ward, 2000; Unsworth & Engle, 2007).

## Toward a Unification of IFR and ISR

However, more recently, a number of studies have shown that when the two tasks are performed under identical methodologies, list lengths, and scoring systems, performance in the two tasks is much more similar than different over a wide range of variables. For example, Bhatarah, Ward, Smith, and Hayes (2009) showed similar effects of rehearsal, presentation rate, word length, and articulatory suppression on the two tasks; Spurgeon, Ward, and Matthews (2014a) found similar effects of phonological similarity on the two tasks; Spurgeon, Ward, Matthews, and Farrell (2015) found similar effects of temporal grouping on the two tasks; and Grenfell-Essam and Ward (2012) have shown similar effects of knowledge of list length and test expectancy (see also Bhatarah, Ward, & Tan, 2008). Finally, there is clear evidence of forward ordered recall even in IFR, where participants could recall in any order (Bhatarah et al., 2008; Grenfell-Essam & Ward, 2012; Ward, Tan, & Grenfell-Essam, 2010, see also Beaman & Jones, 1998; Golomb, Peelle, Addis, Kahana, & Wingfield, 2008; Klein, Addis, & Kahana, 2005).

Importantly, we have consistently found the list length has a large effect on the output order in the two tasks. At short list lengths, both tasks show “ISR-like” recall, that is, a strong tendency to initiate recall with the first list item and proceed in a forward order. When participants initiate recall with the first list item, the resultant serial position curves show forward serial recall and more generally elevated recall of the early list items. At longer list lengths, both tasks show “IFR-like” recall, that is, a strong tendency to initiate recall with one of the last four items in the list, and the resultant serial position curves show extended recency effects.

This growing body of supporting evidence has led us to conclude that many of the differences previously attributed to IFR and ISR were actually due to the differences in the list lengths used rather than due to differences in the memory mechanisms underpinning performance on the two tasks. Consistent with the motivation behind these studies, there have been a number of attempts to model both tasks within a single unified theory (Anderson, Bothell, Lebiere, & Matessa, 1998; Brown, Neath, & Chater, 2007; Farrell, 2012; Grossberg & Pearson, 2008).

## The Modality Effect in IFR and ISR

In this article, we focus our attention on the magnitude, extent, and explanation of the modality effect in IFR and ISR. That the modality effect is robust is not in question. It is consistently observed in both ISR (e.g., Campbell & Dodd, 1980; Conrad & Hull, 1968; Corballis, 1966; Cowan, Saults, & Brown, 2004; Cowan, Saults, Elliott, & Moreno, 2002; Crowder, 1970; Crowder & Morton, 1969; Greene & Crowder, 1984; Harvey & Beaman, 2007; Henmon, 1912; Laughery & Pinkus, 1966; Madigna, 1971; Margrain, 1967; Metcalfe & Sharpe, 1985; Murray & Roberts, 1968; Nairne & McNabb, 1985; Nairne & Walters, 1983; Routh, 1970, 1971; Sherman & Turvey, 1969; Spoehr & Corin, 1978; Watkins & Watkins, 1973, 1980; Watkins, Watkins, & Crowder, 1974) and IFR (e.g., Beaman & Morton, 2000; Craik, 1969; Engle, Clark, & Cathcart, 1980; Gardiner, Gathercole, & Gregg, 1983; Glenberg, 1984; Marks & Crowder, 1997; Murdock & Walker, 1969; Nilsson, Wright, & Murdock, 1979; Roberts, 1972; Shand & Klima, 1981; Watkins, 1972; Watkins et al., 1974; Wong & Blevings, 1966).

The modality effect is a particularly interesting phenomenon to examine within an integrated outlook to IFR and ISR because although modality effects are consistently observed in both tasks, there are characteristic differences in the magnitude and the extent of the observed effect in IFR and ISR. In all these studies, there were consistent recall advantages for items presented at the end of the list when the list is spoken compared to when the list is presented visually and these auditory recall advantages are not present at the prerecency serial positions. However, when one examines the modality effects more closely, a number of authors (e.g., Penney, 1975, 1989; Watkins & Watkins, 1973, 1980; Watkins et al., 1974) have noted subtle differences between the magnitude and the extent of the modality effects in the two tasks: the modality effect in ISR is often large in magnitude and limited to the final list item (e.g., Conrad & Hull, 1968; Corballis, 1966), whereas the modality effect in IFR is often smaller in magnitude but is spread over multiple end-of-list items (e.g., Craik, 1969; Engle, 1974; Murdock & Walker, 1969). One motivation for the current work was therefore to determine whether these subtle differences resulted from the differences in list length and scoring that are typically used in IFR and ISR.

A second motivation concerns how the modality effects are best explained: whether different ISR- and IFR-specific mechanisms are necessary, or whether the same account can explain the different patterns of modality effects across the two tasks. Many different explanations of the modality effect have been proposed in the literature but currently there is no single agreed explanation. An early approach that has received continued support in many contemporary accounts (e.g., Hurlstone, Hitch, & Baddeley, 2014) assumes that the modality effect reflects the existence and properties of an echoic sensory memory or precategorical acoustic store (PAS, Crowder & Morton, 1969) that can be used to augment recall from a modality-independent short-term memory (STM) store. The main feature of PAS, as originally construed, was that it was capable of holding auditory verbal information for at least a few seconds, sufficiently long to affect immediate memory tasks. Within the PAS model, information is lost in two ways: displacement or overwriting by subsequent events, and through temporal decay. The modality advantage arises because auditory presentation supplies participants with additional information compared with visual information that still persists at the time of retrieval. Murdock (1967) and Murdock and Walker (1969) propose that this auditory advantage arises due to the greater *capacity* of the auditory store relative to the visual store, and Craik (1969) argued that auditory advantage arises due to the greater *persistence* of the contents of the auditory store. He claimed that participants output from STM before auditory memory, explaining why there was little difference in recall between the visual and auditory modalities when recall was initiated at the end of the list, and why the magnitude of the modality effects are far more pronounced when recall is initiated from the start of the list.

A key line of evidence in support of an auditory sensory store approach is the finding that an irrelevant stimulus speech suffix, which would mask or overwrite the final item, leads to a substantial reduction in recall of terminal items (e.g., Crowder, 1967; Crowder & Raeburn, 1970; Dallett, 1965; Morton, 1968). Whereas, if the suffix is visual, or a nonspeech sound, the suffix effect is greatly reduced or abolished, presumably, because the final list item remains unmasked (Crowder, 1971; Morton & Holloway, 1970). Additionally, evidence that the suffix is stored in a precategorical store comes from Morton, Crowder, and Prussin (1971) who found that postcategorical features of a suffix (meaning, frequency, and emotionality) did not affect the size of the suffix effect. Further evidence comes from the finding that no modality effect occurs with homophones, which will create the same echoic trace (Crowder, 1978). It should be noted that the Grossberg and Pearson (2008) LIST-PARSE model is the only integrated account of IFR and ISR that attempts to explain modality effects. It also assumes that modality effects arise from direct output from a transient sensory memory that is distinct from a cognitive or motor working memory.

While there was considerable early support for PAS, there has been subsequent evidence that clearly questions whether the modality effect can be attributed to a sensory store that is precategorical, acoustic or a store. Ayres, Jonides, Reitman, Egan, and Howard (1979; see also Neath, Surprenant, & Crowder, 1993) found the strength of the suffix effect was affected by whether or not the participants were led to believe that an auditory suffix was speech or a nonspeech sound. As PAS was considered precategorical, it cannot account for context-dependent suffix effects. Suffix effects have also been found with nonacoustic suffixes, thus bringing into question the acoustic nature of PAS (Campbell & Dodd, 1980; Greene & Crowder, 1984; Nairne & McNabb, 1985; Nairne & Walters, 1983; Shand & Klima, 1981; Spoehr & Corin, 1978). Finally, the labeling of the model as a “store” has also been challenged due to the inverse duration effect and the empirical separation of stimulus persistence from information persistence (Surprenant & Neath, 2009).

An alternative explanation has been proposed by Cowan and colleagues (Cowan et al., 2004; Cowan et al., 2002), who posit that the modality effect is mainly due to the greater resistance of auditory items in the sensory store to output interference. Cowan, Saults, Elliott, and Moreno (2002) presented participants with nine-item digit lists and cued participants to initiate silent recall at either serial positions 1, 4, or 7. They found that under low output interference (recall initiated from serial position 7), modality effects were small, whereas under high output interference (recall initiated from serial position 1) modality effects were much larger. Cowan, Saults, and Brown (2004) revisited this earlier work using recall involving auditory components to investigate whether the differing effect of output interference was related to the absence of acoustic interference during recall. They also performed analyses which allowed detection of modality differences under low output interference conditions due to ceiling performance. In contrast to Cowan et al. (2002), Cowan et al. (2004) found that the magnitude of the modality effect was very similar under high and low output interference conditions when the correction for ceiling performance at low output interference conditions was applied. Nilsson, Wright, and Murdock (1979) also attribute modality effects to the greater resistance of auditory items to output interference. They used mixed-modality lists, where both auditory and visual list items were presented randomly within each list, and found that when recalled second, the recall of visual items decreased by 40% but the recall of auditory items only decreased by 8%.

One difficulty for accounts that predict auditory recall advantages that are exclusively available to the last list item(s) is that modality effects can be observed using methodologies in which an auditory sensory store explanation is untenable. For example, a modality effect is observed in the continuous distractor free recall paradigm, where an auditory distractor item is presented before and after every list item (Gardiner & Gregg, 1979; Glenberg, 1984). Under such conditions, one might presume that the auditory distractor would displace the contents of echoic memory for both visual and auditory to-be-remembered list items. Nevertheless, both studies showed clear auditory advantages for the recency items.

This difficulty can be overcome if one assumes that the effect of modality is not limited to the item at the end of the list. According to Nairne’s feature model (1988, 1990, see also Neath & Nairne, 1995; Neath, 2000), different groups of items are represented in memory by vectors containing their features, or attributes. Items within the same group overwrite the preceding item within the same group, and auditory items are assumed to be encoded with greater number of e features than visual items. Although the feature model assumes that all auditory items in a list are more richly encoded than all visual items, the overwriting mechanism and retrieval dynamics ensure that there is an auditory recency advantage only for the last item within a list, or if a list is grouped, the last item from each group.

Alternatively, Glenberg and Swanson (1986) favor a temporal distinctiveness explanation for modality effects, in which temporal information is encoded as part of the memory trace of a to-be-remembered item. Modality effects are assumed to occur because stored temporal information is relatively fine grained trace for all auditory items but more coarse grained trace for visually presented items (Gardiner, 1983). A related hypothesis is provided by Henson’s (1998) Start End Model who assumes that an item’s position within the list is encoded relative to the start and end of the list using start and end markers. This positional information produces a token in STM. Retrieval involves using positional cues for each location and selecting the token that overlaps most with the cue. Modality effects are explained as a function of stronger positional coding for auditory items compared with visual items. This is achieved by increasing the strength of the end marker for auditory items. Henson emphasizes that this explanation is rather ad hoc and should be seen as a first step to modeling modality effects.

A more fundamental concern for complete theoretical explanations of the modality effect arises from studies that show an *inverse modality effect:* the observation that prerecency *visual* items can sometimes show a *recall advantage* relative to the prerecency *auditory* items (e.g., Beaman, 2002). To date, there has not been a systematic analysis of all experiments reporting modality effects. Nevertheless, of the 44 cited articles within the current article that report standard modality effects, eight reported an inverse modality effect (either verbally or statistically; Beaman, 2002; Craik, 1969; Engle, 1974; Glenberg, 1984; Macken, Taylor, Kozlov, Hughes, & Jones, 2016; Marks & Crowder, 1997; Metcalfe & Sharpe, 1985; Watkins & Watkins, 1973), a further 13 showed a prerecency visual advantage in the results but this effect was not noted (Beaman & Morton, 2000; Conrad & Hull, 1968; Corballis, 1966; Engle, Clark, & Cathcart, 1980; Gardiner, Gathercole, & Gregg, 1983; Gardiner & Gregg, 1979; Harvey & Beaman, 2007; Henson, 1998; Murdock & Walker, 1969; Nairne & McNabb, 1985; Watkins, 1972; Watkins & Waktins, 1973; Watkins, Watkins, & Crowder, 1974), whereas 15 showed no evidence of a prerecency visual advantage. Finally, in eight cases it was not possible to determine whether or not inverse modality effects were present from the results that were provided.

Theoretical interpretations of inverse modality effects are in their infancy. Within the feature model, Figure 4 of Nairne (1990) suggests that the inverse modality effect may arise with lower values of *a*, the attentional scaling parameter,^1^ but disappear as the value of this scaling parameter increases. A key role for attention in both standard and inverse modality effects has also recently been provided by Macken, Taylor, Kozlov, Hughes, and Jones (2016). They used the reconstruction of order task, in which sequences of seven consonants were presented sequentially, and at test, participants saw the same seven study items in a new random order and were required to indicate the original order by clicking on each item in turn with a computer mouse. The most clearly interpretable comparisons are those between the serial reconstruction of the auditory and the visual silent lists, where Macken et al. (2016) observed the standard modality effect (the recall advantage at the end of the list for the auditory modality) and a significant inverse modality effect, a visual silent recall *advantage* relative to the auditory condition in the middle portion of the serial position curve. Subsequent experiments showed that the inverse modality effect was clearly present in control conditions but abolished under articulatory suppression (Experiment 2) and present at standard (750 ms per item) and slower rates (1,500 ms per item) but abolished at very fast rates (375 ms per item, Experiment 3).

Macken et al.’s (2016) Experiment 1 also examined serial reconstruction of order using visual mouthed and visual vocalized (read aloud) conditions. Interpreting the differences in serial position curves between these other conditions is made more difficult because serial recall performance in the prerecency serial positions was far lower for the read aloud and visual mouthed conditions than for either the visual silent or the auditory conditions. The authors argued that the IME were abolished in these two conditions, but this conclusion seems somewhat contentious given that, for whatever reason, the visual silent prerecency advantage is far more apparent when compared with the visual mouthed and read aloud than when the visual silent is compared with the auditory condition.

What is clear is that there can be significant and reliable modality effects in which auditory items are recalled better than visual silent items at the end of the list, and inverse modality effects in which visual silent items are recalled better than auditory counterparts at early mid prerecency positions. Together with the differences that occur within the other two presentation modalities, this work suggests that there are multiple differences that can occur when participants are presented with stimuli in different presentation conditions and that these effects can occur across the entire serial position curve.

Macken et al. (2016) argue that the modality of presentation affects the way in which individual to-be-remembered items are perceptually organized into objects (see also Hughes, Marsh, & Jones, 2009; Jones, 1993; Jones, Hughes, & Macken, 2006; Nicholls & Jones, 2002). Specifically, stronger obligatory object formation is assumed to occur for auditory items relative to visual items. One consequence is that a new auditory item is more likely to be organized into the current auditory sequenced object, whereas a new visual item is more easily isolated and manipulated such that it can be incorporated into a cumulatively expanding subsequence through verbal rehearsal. Primacy and recency emerge because items are more readily addressable at the object boundaries and less addressable in the object interior.

According to Macken et al. (2016) the standard auditory recency advantage in serial reconstruction arises because recall of the last auditory item is more likely to benefit from the enhanced addressability at the object boundary, whereas the last visual item is less likely to have been incorporated into the object. The inverted modality effect in serial reconstruction emerges because it is assumed to be easier to rehearse each newly presented item into a cumulatively expanding rehearsal set when the list items are visual, and harder to isolate and manipulate newly presented auditory items because they are more likely to be incorporated into the current object. In line with these hypotheses, the inverse modality effect is reduced or eliminated under conditions that interfere with cumulative forward ordered rehearsal, such as concurrent articulation and very fast presentation (Macken et al., 2016).

## Examining Modality Effects at Different List Lengths

The current studies examine: (a) the role of list length in determining the magnitude and extent of the modality effect, (b) the effects of where participants initiate their recall in the list, and (c) the resultant effect this has on performance in explaining modality effects. Visual silent conditions are contrasted with auditory (Experiment 1) and read aloud (Experiment 2) conditions.

At a more general level, theories of modality effects have often been associated with specific methodologies (e.g., the feature model, Nairne, 1988, 1990; ISR), or have been assumed to apply to both IFR and ISR but have not as yet been empirically examined across a wide range of list lengths. We were interested in whether the list length manipulation could also help explain why there are often subtle differences between the modality effects typically observed in IFR and ISR. If we could attribute these differences to the scoring systems or list lengths used, then this would help support our earlier claims for theoretical unification of the two tasks.

We were also interested in whether inverse modality effects observed with consonants in serial reconstruction of order (cf. Macken et al., 2016) could so readily be observed with experiment-unique word sets in IFR and ISR, and if so, whether these effects would be more or less apparent when visual silent conditions are contrasted with auditory conditions in Experiment 1 than when visual silent conditions are contrasted with read aloud conditions in Experiment 2.

We were also interested in whether the analyses that we typically perform could shed light on the locus of the standard modality effect on the two tasks. We were particularly interested in whether the presentation modality interacted with the participants’ choice of first word recalled. Our earlier work showed that the serial position curves are strongly affected by the first recall (e.g., Grenfell-Essam & Ward, 2012; Ward et al., 2010), and that the first word recalled was affected by the list length and task instruction. Based on our earlier research, we considered three reasons why there could be differences in the modality effects between IFR and ISR.

First, there may be modality differences in the initial starting point of recall in the list. When output order is constrained to either begin at the start, or begin at the end there are large differences in the modality effect (Craik, 1969; Metcalfe & Sharpe, 1985; Nilsson et al., 1979). Reduced modality effects are found when recall is initiated from the end of the list compared with the start. We already know the tendency to initiate recall with the first item decreases with increasing list length, whereas the tendency to initiate recall with one of the last four items increases with increasing list length (Grenfell-Essam & Ward, 2012, 2015; Grenfell-Essam, Ward, & Tan, 2013; Spurgeon et al., 2014a, 2014b; Spurgeon et al., 2015; Ward et al., 2010). In auditory trials, participants may be more likely to initiate their recall with one of the last four items in the list compared to visual trials; thus leading to greater recall of other auditory items, enhancing the recency advantage for auditory lists.

Second, participants may be equally likely to initiate their recall with one of the last four list items, but there may be differences in how far back they start. Murdock and Walker (1969) found with auditory presentation there was a peak four words back from the end of the list, whereas with visual presentation there was a large increase for the final word. Nilsson, Wright, and Murdock (1975) conducted lag analyses on the Murdock and Walker (1969) data and interpreted the differences between the visual and auditory items as reflecting backward output order (visual items) and forward output order initiated near the end of the list (auditory items; see also Nilsson et al., 1979). In auditory trials, participants may choose to initiate their recall with a nonterminal list item (e.g., starting with list item *n* − 3 or *n* − 2, where *n* is the list length) compared with visual items (e.g., starting with list item *n* or *n* − 1). This would lead to participants in auditory trials recalling a longer run of terminal list items than for visual items. If this were the case then one might expect to see a graded recency effect in the first recall data for visual items, but a rather different trend for the auditory items, such that recall might tend to start with nonterminal list items more often than the final item in the list with auditory items.

Finally, the modality effect might be due to greater persistence of the recency items when presented auditorily compared with visually (Beaman & Morton, 2000; Cowan et al., 2004; Cowan et al., 2002; Craik, 1969; Nilsson et al., 1979). If this is the case, then one might expect to see no difference in the first recall data, but a large modality effect in the resultant serial position curves where recall was initiated with the first word, and a much reduced, or abolished modality effect in the resultant serial position curves where recall was initiated with one of the last four words.

## Experiment 1

In Experiment 1, we examined both IFR and ISR, while manipulating presentation modality and list length. There were two groups of participants: One group performed only ISR, and the other only IFR. All participants were presented with five auditory trials and five visual trials of each of the seven different list lengths (lists of two, four, five, six, eight, and 12 words). Presentation modality was blocked, and counterbalanced across participants, such that in one half of the experiment the participant experienced only visual trials, and in the other half of the experiment the participant experienced only auditory trials. In the visual trials, participants saw the words appear on screen, and were instructed to read each word silently as it was presented. In the auditory trials, participants heard the words through headphones, and were instructed to listen to each word silently as it was presented. The list lengths of the trials were randomized within each block such that each list length was presented five times. This also resulted in participants not knowing the length of the list in advance of its presentation (note that prior work suggests that advance knowledge of the list length does not greatly affect our IFR and ISR data; Grenfell-Essam & Ward, 2012).

### Method

#### Participants

Forty participants from the University of Essex took part in this experiment. There were five males and 35 females ranging from 18 to 39 years of age. The mean age was 20.8 years (standard deviation = 4.2). All participants confirmed that they were fluent in English.

#### Materials and apparatus

The materials consisted of a subset of 440 words that were randomly selected for each participant from the Toronto Noun Pool (Friendly, Franklin, Hoffman, & Rubin, 1982). Audio file versions of each word were obtained from Michael Kahana’s Computational Memory Laboratory web site (Kahana, 2010; http://memory.psych.upenn.edu/WordPools). The shortest sound file was 0.350 s; the longest sound file was 0.860 s. Audio files were presented via a Logitech USB Headset 4.330 at a volume comfortable to each participant. The words were presented in 60-point Times New Roman font. The materials were presented on an Apple eMac computer monitor using the Supercard application.

#### Design

The experiment used a mixed design. The between-subjects independent variable was the task with two levels (ISR or IFR). There were three within-subjects independent variables: modality with two levels (visual and auditory), list length with seven levels (two, four, five, six, seven, eight, and 12), and serial position with up to 12 levels (serial position 1–12). The main dependent variables were the proportion of words recalled in any order (FR scoring), the proportion of words recalled in the correct serial position (SR scoring), and the probability of initiating recall with the very first list item—that is, Probability of First Recall (PFR) = Serial Position 1.

#### Procedure

Participants were tested individually and informed that they would be shown two practice lists, of seven words each, followed by 70 experimental lists of words. Participants were allocated to one of two groups: either the ISR group (where they should remember the words in the correct order), or the IFR group (where they can remember the words in any order). The two practice trials consisted of one visual trial and one auditory trial. The experimental trials were split into two equal blocks of 35 trials each. Each block consisted of only visual trials or auditory trials. The order of the blocks was counterbalanced across participants. In all conditions, the order of the list lengths was randomized, such that each block contained five repetitions of each list length. Participants were not aware of the length of the list in advance of its presentation.

Each trial started with a fixation cross displayed for 2 s, followed after 1 s by a sequence of between two and 12 words; presented one at a time in the center of the computer screen (visual trials) or auditorily through headphones (auditory trials). The presentation rate was one word every second. In the visual trials, each word was displayed for 750 ms with an additional 250 ms interstimulus interval in which the stimulus field was blank. Participants were instructed to read each word silently as it was presented. In the auditory trials, each sound file was played from the start of this time until it was finished. The remaining time until 1 s elapsed was filled with silence. Participants were instructed to listen to each word silently as it was presented. For both visual and auditory trials, participants were instructed to ensure that they did not mouth or whisper the stimuli. After the last item had been presented, an empty grid appeared on screen that contained the same number of numbered rows as there had been words presented on that trial, to inform participants of the list length of that trial. Participants were instructed to recall as many words as they could on the paper response sheet (which always contained 12 rows). Trials had no maximum recall period; the participants ended recall when they felt they had remembered all the words that they could.

Participants performing IFR were free to write their words in any order they wished and filled their response grids from the top of the grid. Participants performing ISR were asked to try and start their recall with the first item and proceed in forward serial order working down the grid writing each word in the row that corresponded to that items’ serial position. If they could not remember the first item, they were asked to remember the earliest item that they could and try to write it on the corresponding row. They were asked not to fill in earlier responses following later responses. The experimenter was present in the room for the two practice trials and ensured that all instructions were obeyed. Once the experimenter was confident that the participants would adhere to the instructions, they left the room and allowed the participant to begin the experimental trials.

### Results

There were two types of scoring an item correct: a recalled word was scored as correct using *FR scoring* if it had been presented at any position in the immediately preceding list, a recalled word was scored as correct using *SR scoring* if it had been written in the response grid in the numbered row corresponding to that serial position in the immediately preceding list. The data were considered in three different analyses: serial position curves, probability of first recall (PFR), and resultant serial position curves. The current experiment is specifically interested in differences in modality across all serial positions (to determine both modality effects and inverse modality effects) and as such these are planned comparisons. Therefore, simple main effects were conducted, even after nonsignificant interactions, to find if any significant differences existed at any serial position. All of the Appendixes mentioned in this Results section can be found within the supplementary material that accompanies this article. Due to the large numbers of comparisons, we have adjusted the significance levels with Bonferroni corrections.

Before undertaking the three main analyses, we first performed a preliminary analysis to determine whether the order of the visual block of trials and auditory block of trials influenced the serial position curves and the probability of first recall data. As detailed in supplementary Appendix A, we found that there was little or no effect of block order on any of these analyses, and so we collapse across the block order in the following analyses.

#### Analyses of the serial position curves of all the data

**Figure 1 fig1:**
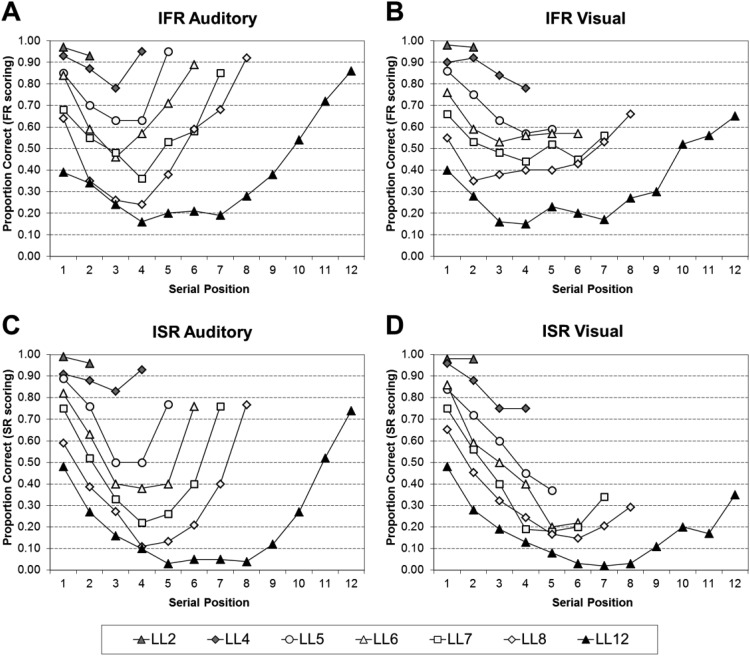
Experiment 1. Overall serial position curves for the auditory and visual IFR conditions (using FR soring) and ISR conditions (using SR scoring).

Figure 1 shows the serial position curves for IFR auditory using FR scoring (Panel A), IFR visual using FR scoring (Panel B), ISR auditory using SR scoring (Panel C), and ISR visual using SR scoring (Panel D). For both IFR and ISR, recall is close to ceiling for very short list lengths, but as the list length increases so the curves become more bowed; showing primacy and recency effects for the auditory conditions, but much reduced recency in the visual conditions.[Fig-anchor fig1]

The serial position curves were analyzed by a series of 2 (Task: ISR or IFR) × 2 (Modality: Auditory and Visual) × *n* (Serial Position, where *n* is the list length) mixed ANOVAs; full statistical analyses for each list length can be found in the supplementary Appendix B1. To summarize the main points, all of the main effects of task, and the two-way interactions between task and modality were nonsignificant. Thus the performance in the tasks does not differ significantly, nor does performance differ significantly as a function of modality and task.

The statistical analyses were then further broken down by task into a series of 2 (Modality: Auditory and Visual) × *n* (Serial Position, where *n* is the list length) within-subject ANOVAs; full statistical analyses for each list length can be found in supplementary Appendix B2 for IFR using FR scoring, supplementary Appendix B3 for ISR using FR scoring, and supplementary Appendix B4 for ISR using SR scoring. Table 1 visually simplifies the significant findings from supplementary Appendixes B2–4. To summarize, the modality effect is present in both tasks at all list lengths (LL)—except LL2. The simple main effects showed that the modality effect was very similar in both tasks: capturing only the final serial position in short lists, but extending back to the final two or three serial positions at longer lists.[Table-anchor tbl1]

Interestingly, inverse modality effects were observed in early list positions at longer list lengths for both tasks when using free recall scoring. These pairwise comparisons reached significance at Serial Position 4 at LL8 (for IFR with FR scoring) and Serial Positions 2, 4, and 5 at LL12 (for ISR with FR scoring).

**Figure 2 fig2:**
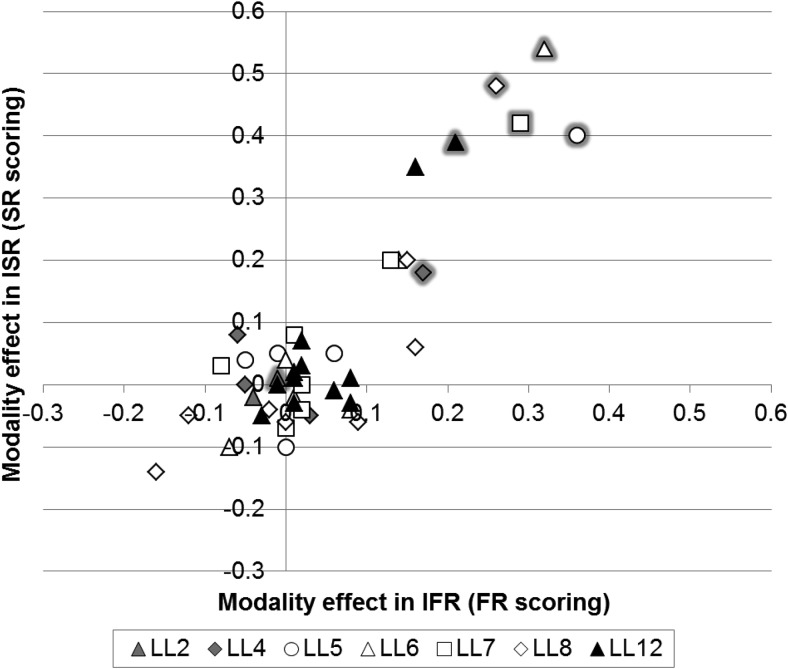
Experiment 1. Scatterplot showing the magnitude of the modality effect (difference between auditory and visual recall) for IFR against ISR for each list length and serial position. Note that symbols with gray edging denote the final serial position at each list length.

We were interested in exploring the overall recall patterns further in order to determine whether the magnitude of the modality effect was similar across tasks and list lengths. Figure 2 plots the magnitude of the modality effect (the difference scores of auditory–visual conditions) for IFR on the *x*-axis, using FR scoring, against ISR on the *y*-axis, using SR scoring, for each list length at each serial position. This figure allows for a direct magnitude comparison across serial positions at different list lengths. Symbols with gray edging denote the final serial position at each list length. The large negative values in the lower left hand quadrant of Figure 2 reflect instances of significant inverse modality effects. The figure shows that the largest modality effects are found for the final serial position and that the magnitude of the modality effect is broadly similar across list lengths and tasks, particularly at LL5–12 where performance is not at ceiling. A Pearson’s correlation, performed to directly compare the magnitude of performance for IFR and ISR at the same list lengths and serial positions, resulted in a highly significant, strong, positive correlation, *r*(44) = 0.858, *p* < .001. This indicates that performance in IFR and ISR is highly related across list lengths and serial positions.[Fig-anchor fig2]

#### The probability of first recall (PFR)

**Figure 3 fig3:**
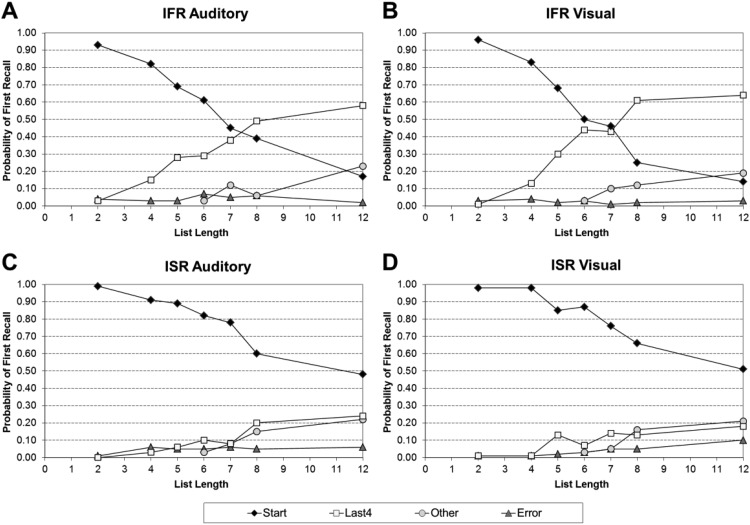
Experiment 1. Probability of first recall (PFR) data for the auditory and visual IFR and ISR conditions.

The main PFR findings are summarized in Figure 3 (Panel A: auditory IFR; Panel B: visual IFR; Panel C: auditory ISR; Panel D: visual ISR). The full values these figures are based on can be found in supplementary Appendix C1 for IFR and supplementary Appendix C2 for ISR. Figure 3 collapses the data into the four categories used in Ward et al. (2010): “Start” (those trials that started with the first word presented in the list), “Last 4” (those trials that started with any one of the last four list items), “Other” (those trials that started with any of the other list items), and “Error” (those trials in which nothing was recalled or where recall began with an error).[Fig-anchor fig3]

Importantly, the tendency to initiate recall with the first list item is unaffected by the modality of list presentation, but is strongly affected by the task. In IFR, the tendency for both visual and auditory lists was to initiate recall with the first item is present up to and including LL7, but at longer list lengths the modal tendency was to initiate recall with one of the last four items. With ISR, there was again no effect of modality on the initial recall but consistent with instructions, participants’ modal tendency was to start with the first item even at the longest lists.

Table 2 summarizes the findings of a series of two 2 (Task: ISR or IFR) × 2 (Modality: Auditory and Visual) × 7 (List Length: 2, 4, 5, 6, 7, 8, and 12) mixed ANOVAs that were performed on the proportion of trials where recall started with Serial Position 1, and the proportion of trials where recall started with one of the last four serial positions. In both analyses, there was a significant main effect of task, a nonsignificant main effect of modality, and a significant main effect of list length. This confirmed that participants were more likely to initiate their recall with Serial Position 1 in the ISR conditions compared to the IFR conditions, and one of the last four serial positions in the IFR conditions compared to the ISR conditions.[Table-anchor tbl2]

In both analyses the three-way interactions between task, modality, and list length were nonsignificant, as were the two-way interactions between task and modality, and modality and list length. However, in both analyses, the two-way interactions between task and list length were significant. Simple main effects showed that participants were more likely to initiate their recall with Serial Position 1 in ISR compared with IFR at all list lengths, except two and four, and with one of the last four in IFR compared with ISR at all list lengths, except two and four. Thus, modality had no overall, or interactive, effect so there is little evidence that the modality effect is due to an increased likelihood of starting toward the end of the list with auditory lists compared with visual lists.

There is an additional, subtler way that the modality effect could be caused. Within the last category in Figure 3, participants might start further back from the end of the list in the auditory condition compared with the visual condition. Table 3 shows how far back participants started their recall for the last four items over LL6–12.[Table-anchor tbl3]

A chi-square was performed on this data. Due to the auditory ISR cell at position *n* containing 0 responses, *n* − 3 was grouped with *n* − 2, and *n* − 1 was grouped with *n*. There were significant differences between IFR and ISR in the initial item output: Participants were more likely to start with either *n* − 1 or *n*, compared with *n* − 3 or *n* − 2, in IFR than ISR for both auditory (χ^2^ = 32.9, *p* < .001) and visual (χ^2^ = 12.7, *p* < .001) modalities. These differences are not that surprising because they reflect the different constraints placed on recall between the two tasks. In ISR, participants are not allowed to recall earlier items and so initiating recall with one of the last four items in ISR reflects suboptimal performance; participants’ optimal starting point is Serial Position 1. In line with this, participants performing ISR started with one of the last four less than 15% of the time, but started with Serial Position 1 69% of the time.

However, more importantly, there were no significant differences in how far back participants started in the auditory compared to visual conditions for either IFR (χ^2^ = 0.724, *p* = .395), or ISR (χ^2^ = 1.53, *p* = .216), confirming that there is little evidence that the modality effect was due to participants starting further back from the end of the list in the auditory condition, compared to the visual condition, for either ISR or IFR.

#### The effect of the first word recalled on the resultant serial position curves

**Figure 4 fig4:**
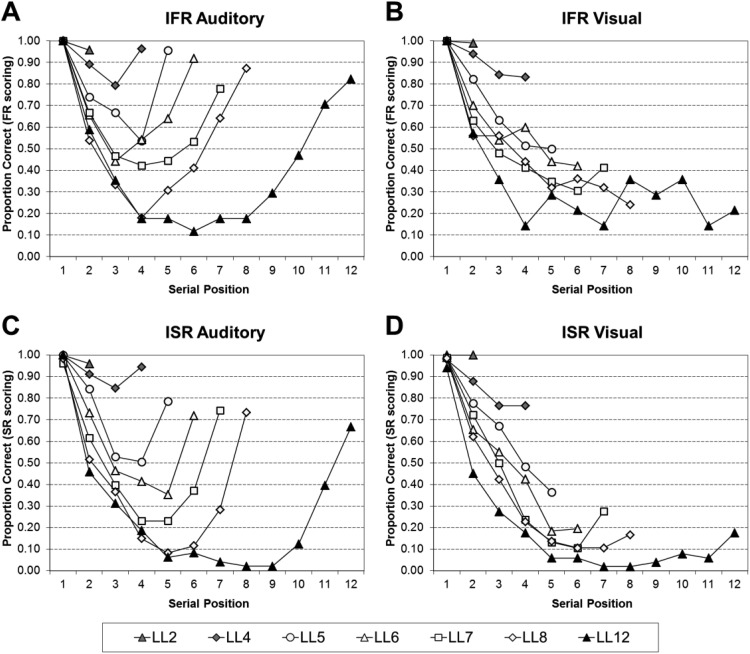
Experiment 1. Resultant serial position curves for trials that began with Serial Position 1 for the auditory and visual IFR conditions (using FR soring) and ISR conditions (using SR scoring).

Figure 4 shows the resultant serial position curves given recall was initiated with Serial Position 1 for IFR auditory using FR scoring (Panel A), IFR visual using FR scoring (Panel B), ISR auditory using SR scoring (Panel C), and ISR visual using SR scoring (Panel D). For both IFR and ISR when recall is initiated with Serial Position 1 the resulting recall shows elevated primacy levels and extended recency in the auditory conditions, but little to no recency in the visual conditions. There is some evidence in IFR that at particular list lengths (LL6 and 8) that an inverse modality effect is occurring, in which prerecency serial positions in the visual modality are superior to the auditory modality. It appears as though auditory recency items may be less susceptible to output interference compared with the visual recency items.[Fig-anchor fig4]

The resultant serial position curves were analyzed by a series of 2 (Task: ISR or IFR) × 2 (Modality: Auditory and Visual) × *n* − 1 (Serial Position: 2 − *n*, where *n* is the list length) mixed ANOVAs. Full statistical analyses for each list length can be found in supplementary Appendix B5 for trials in which recall was initiated with Serial Position 1. To summarize the main points, all of the main effects of task, and the two-way interactions between task and modality were nonsignificant. Thus, given that recall was initiated with Serial Position 1 performance in the tasks does not differ significantly, nor does performance differ significantly as a function of modality and task.

The statistical analyses were then further broken down by task into a series of 2 (Modality: Auditory and Visual) × *n* (Serial Position: 2 − *n*, where *n* is the list length) within-subject ANOVAs; full statistical analyses for each list length can be found in supplementary Appendix B6 for IFR using FR scoring, supplementary Appendix B7 for ISR using FR scoring, and supplementary Appendix B8 for ISR using SR scoring. Table 4 visually simplifies the significant findings from supplementary Appendixes B6–8. The most important finding is that the modality effect is still very much present in the resultant serial position curves when recall was initiated with Serial Position 1. There were a few cases where the apparent inversion in the modality effect at prerecency serial positions did reach significance (LL5 and 8).[Table-anchor tbl4]

**Figure 5 fig5:**
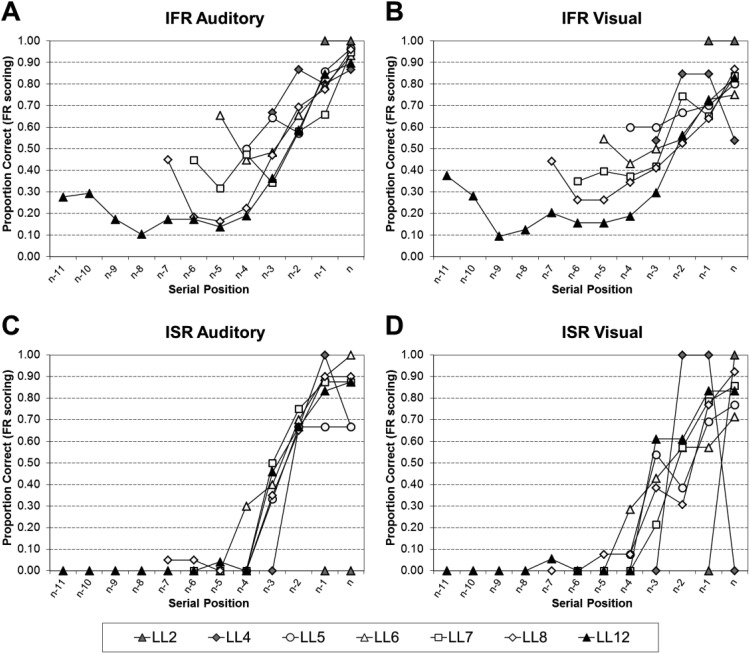
Experiment 1. Resultant serial position curves for trials that began with one of the last four words for the auditory and visual IFR conditions (using FR soring) and ISR conditions (using FR scoring).

Turning now to the resultant serial position curves given recall was initiated with one of the last four words; Figure 5 shows the resultant serial position curves given recall was initiated with any one of the last four words for IFR auditory (Panel A), IFR visual (Panel B), ISR auditory (Panel C), and ISR visual (Panel D). All Panels use FR scoring. For both IFR and ISR when recall is initiated with one of the last four words the resulting recall shows reduced primacy and extended recency in both the auditory and visual conditions. It appears as though the modality effect has been greatly reduced/eliminated at most list lengths.[Fig-anchor fig5]

The resultant serial position curves were analyzed by a series of 2 (Task: ISR or IFR) × 2 (Modality: Auditory and Visual) × *n* (Serial Position, where *n* is the list length) mixed ANOVAs. Full statistical analyses for trials in which recall was initiated with one of the last four words, for each list length, can be found in supplementary Appendix B9 for all serial positions, and in supplementary Appendix B10 limited to only the final four serial positions. Supplementary Appendix B10 was included as due to the nature of the forward ordered constraint on recall in ISR the comparison in supplementary Appendix B9 produced task differences that were due to the constraints on recall. The latter analysis was included as due to the nature of the forward ordered constraint on recall in ISR the comparison in the former analysis produced task differences that were due to the constraints on recall. In summary, the main point to highlight is that when limited to the final four serial positions only, the only significant findings were for the main effect of serial position at LL7 onward. Thus, given that recall was initiated with one of the last four words all task differences and all modality effects disappear.

The statistical analyses were then further broken down by task into a series of 2 (Modality: Auditory and Visual) × *n* (Serial Position, where *n* is the list length) within-subject ANOVAs; full statistical analyses for each list length can be found in supplementary Appendix B11 for the IFR data using FR scoring, and supplementary Appendix B12 for the ISR data using FR scoring. Table 5 visually simplifies the significant findings from supplementary Appendixes B11–B12. The ISR LL4 ANOVA is missing as no participants contributed to this analysis. The most important finding is that the modality effect is greatly reduced/eliminated in the resultant serial position curves when recall was initiated with one of the last four words. Therefore, it appears that the recency advantage for auditorily presented items does not come from trials in which participants initiated their recall with the end items.[Table-anchor tbl5]

### Discussion

This experiment examined the effect of presentation modality on performance in IFR and ISR. We were specifically interested in (a) the role of list length in determining the magnitude and extent of the modality effect, (b) the effects of where participants initiate their recall in the list and (c) the resultant effect this has on performance in explaining modality effects. Finally, we were also interested in whether we could observe inverse modality effects using a very large pool of words with IFR and ISR.

The main finding from this experiment was that the modality effects in IFR and ISR are remarkably similar when equivalent methodologies were used. The current experiment suggests that the previous differences reported in the magnitude and the extent of the modality effects in IFR and ISR were to a greater extent due to the role of list length that is typically used in these studies. When short lists of four to five words were presented, the modality effects for both IFR and ISR were large in magnitude and limited to the final serial position, showing an “ISR-like” modality effect similar to that observed in ISR by Conrad and Hull (1968). However, when longer lists of six to 12 words were presented, the modality effects for both tasks were smaller and often extended over a greater number of later serial positions, showing an “IFR-like” modality effect, reminiscent of the IFR modality effect found by Murdock and Walker (1969).

Our analyses also discriminated between three possible reasons for the modality effect. We found little or no evidence that the modality influenced whether participants’ initiated recall with the first or one of the last few words. Participants in the auditory condition did not show any greater tendency to initiate recall toward the end of the list than in the visual condition. For both visual and spoken lists, participants performing IFR started with the first item with lists of up to seven words, but started with one of the last items from lists of eight words or more. For both visual and auditory trials, participants’ modal response in ISR was to start with the first item in the list (thus obeying the recall instructions).

Neither did we find any modality difference in exactly which of the last four words were recalled first. Contrary to Murdock and Walker (1969) and Nilsson et al. (1975), there was little or no evidence that there were more extended terminal runs of items with spoken rather than visual items. Rather, there was clear evidence of extended recency in the probability of first recall data for both modalities. One possibility for this difference was that the exact output strategy may result from knowledge of the list length. Grenfell-Essam and Ward (2012) found a slight tendency for increased terminal runs when the list length was known (as in Murdock & Walker, 1969) compared with when the list length was unknown (as in this study).

However, we found a large difference in the modality effect in both tasks depending upon which word was first recalled. For both tasks, when participants initiated recall with the first word in the list, a strong modality effect was present. By contrast, when participants initiated recall with one of the last few words in the list, the modality effect was greatly reduced or eliminated for both tasks. Thus, it appears that the presence of the modality effect in both tasks may be related to the greater resilience to output interference of auditory words relative to visual words (Beaman & Morton, 2000; Cowan et al., 2004; Cowan et al., 2002; Craik, 1969; Nilsson et al., 1979).

Finally, we also observed inverse modality effects at early serial positions in longer lists in the two tasks when the same scoring system was used (FR scoring). The effect was obtained in early midserial positions in longer list lengths, similar to those observed by Macken et al. (2016), who found inverse modality effects at Serial Positions 3 and 4 of seven-item lists. This finding is difficult to explain by conventional accounts of the modality effect, as it reflects a visual recall advantage at these list positions. Like Macken et al. (2016), we interpret this finding that the modality of presentation affects the ability of participants to rehearse the list items. Covert rehearsal is possible for both visual silent and auditory presentation modalities, but like Macken et al. (2016), we assume that it may be easier to incorporate a just-presented item into a sequence for cumulative forward ordered recall if it is presented visually rather than if it is spoken.

## Experiment 2

Experiment 2 repeated the methodology of Experiment 1 with a modification to the modality manipulation. In many studies of the modality effect, the auditory condition consists of participants hearing the stimuli (as in Experiment 1). However, in many other studies, the auditory condition consists of participants reading aloud each visually presented word as it appears (e.g., Conrad & Hull, 1968; Gardiner & Gregg, 1979; Metcalfe & Sharpe, 1985; Watkins et al., 1974). In order to more fully address the range of different methodologies used in modality experiments, we felt that it was important to also examine the modality effect under this *read aloud* manipulation.

Although some researchers have claimed that the modality effect is unaffected by whether *auditory* or *read aloud* manipulations are employed (e.g., Crowder, 1970; Henmon, 1912; Penney, 1975; Metcalfe & Sharpe, 1985; Wong & Blevings, 1966), Macken et al. (2016) observed differences in serial reconstruction between auditory and read aloud lists. Specifically, Macken et al. (2016) observed that the recall of the end of the list items was of similar magnitude in the auditory spoken and read aloud conditions, but the recall of the prerecency items was reduced in the read aloud condition relative to both the spoken and the visual silent. Macken et al. (2016) attribute this reduction in prerecency to the reduced ability to freely rehearse in the read aloud condition.

We therefore wanted to test the generality of our conclusions from Experiment 1 (which contrasted visual silent with auditory presentation) in Experiment 2 (which contrasted visual silent with read aloud presentation) to see whether the magnitude and the extent of the modality effects are still broadly similar in IFR and ISR when compared using equivalent methodologies and list lengths. Thus, the only difference between Experiment 1 and Experiment 2 is that we changed the method of presentation in the *auditory* modality condition. Whereas in Experiment 1, participants heard the words through headphones but no visual words were presented, in Experiment 2, the auditory comparison was the *read aloud* modality condition, where participants saw the words presented on the computer screen and were asked to read each word aloud as it appeared.

### Method

#### Participants

Forty participants from the University of Essex took part in this experiment. None had participated in Experiment 1. There were 14 males and 26 females ranging from 20 to 51 years of age. The mean age was 26.9 years (standard deviation = 7.1 years). All participants confirmed that they were fluent in English.

#### Materials and apparatus

The materials used were the same as Experiment 1 excluding the audio files.

#### Design

The design was the same as in Experiment 1 with the exception that the independent within-subjects variable modality had two levels (visual and read aloud).

#### Procedure

The procedure was identical to that used in Experiment 1, with the exception that the auditory practice, and experiential, trials were replaced with read aloud trials. Read aloud trials share exactly the same procedure as visual trials except that participants were instructed to read each word out loud as it appeared on the screen.

### Results

The data were considered in the same three analyses as Experiment 1. We again performed a preliminary analysis to determine whether the order of the visual block of trials and the read aloud block of trials influenced the serial position curves and the probability of first recall data. Once again, we found little or no effect of block order. Therefore, as in Experiment 1, we collapsed across the block order in the analyses that follow. As in Experiment 1, simple main effects were conducted even after nonsignificant interactions, to investigate modality effects, and significant modality effects are displayed on the relevant figures (square markers denote an auditory advantage and cross markers denote a visual advantage). All of the Appendixes mentioned in this Results section can be found within the supplementary material which accompanies this article.

#### Analyses of the serial position curves of all the data

**Figure 6 fig6:**
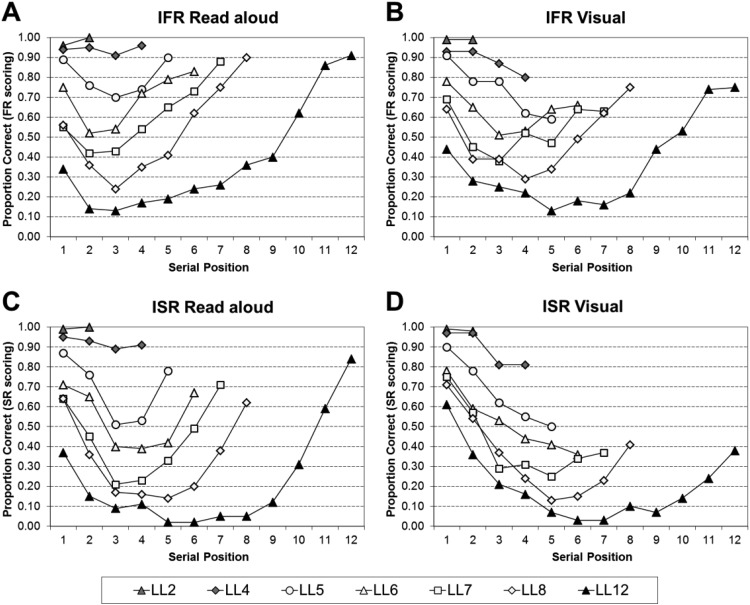
Experiment 2. Overall serial position curves for the read aloud and visual IFR conditions (using FR soring) and ISR conditions (using SR scoring).

Figure 6 shows the serial position curves for IFR read aloud using FR scoring (Panel A), IFR visual using FR scoring (Panel B), ISR read aloud using SR scoring (Panel C), and ISR visual using SR scoring (Panel D). For both IFR and ISR, for very short list lengths recall is close to ceiling, but as the list length increases so the curves become more bowed; showing primacy and recency effects for the read aloud conditions, but much reduced recency in the visual conditions.[Fig-anchor fig6]

The serial position curves were analyzed by a series of 2 (Task: ISR or IFR) × 2 (Modality: Read Aloud and Visual) × *n* (Serial Position, where *n* is the list length) mixed ANOVAs; full statistical analyses for each list length can be found in supplementary Appendix B13. To summarize the main points, all of the main effects of task, and the two-way interactions between task and modality were nonsignificant. Thus, the performance in the tasks does not differ significantly, nor does performance differ significantly as a function of modality and task.

The statistical analyses were then further broken down by task into a series of 2 (Modality: Read Aloud and Visual) × *n* (Serial Position, where *n* is the list length) within-subject ANOVAs; full statistical analyses for each list length can be found in supplementary Appendix B14 for IFR using FR scoring, supplementary Appendix B15 for ISR using FR scoring, and supplementary Appendix B16 for ISR using SR scoring. Table 6 visually simplifies the significant findings from supplementary Appendixes B14–B16. To summarize, the modality effect is present in both tasks at all list lengths—except LL2. Additionally no modality effect was found for LL8 in IFR; the analyses just failed to reach significance. The simple main effects show less coherence between the two tasks. The modality effect in ISR captured only the final serial position in short lists, but extended back to the final two/three serial positions at longer lists. The modality effect in IFR showed some agreement with ISR at short list lengths but was reduced at the longest LLs (LL8 and LL12).[Table-anchor tbl6]

**Figure 7 fig7:**
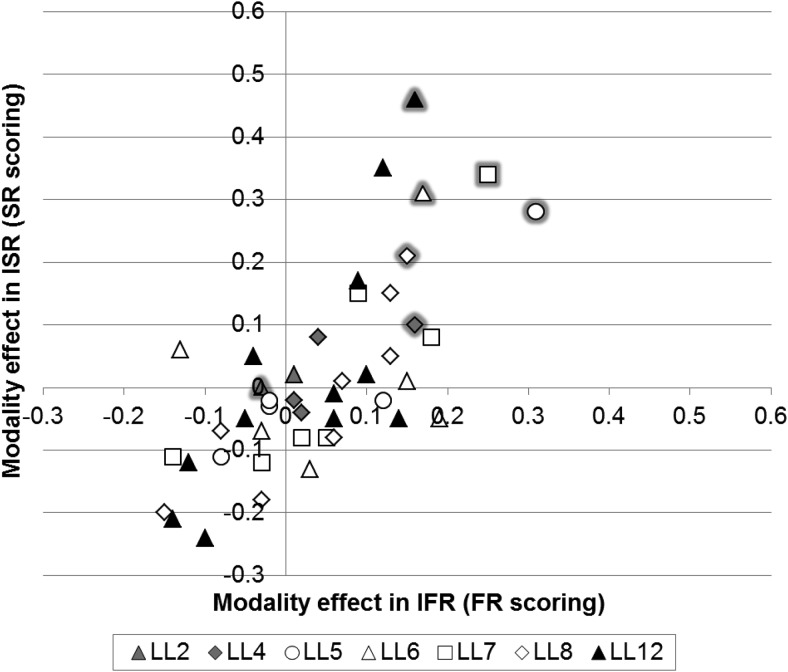
Experiment 2. Scatterplot showing the magnitude of the modality effect (difference between auditory and visual recall) for IFR against ISR for each list length and serial position. Note that symbols with gray edging denote the final serial position at each list length.

We were interested in exploring the overall recall patterns further in order to determine whether the magnitude of the modality effect was similar across tasks and list lengths. Figure 7 follows the format of Figure 2 and plots the magnitude of the modality effect for IFR against ISR for each list length at each serial position. Symbols with gray edging denote the final serial position at each list length. The large negative values in the lower left hand quadrant of Figure 7 reflect instances of significant inverse modality effects. The figure shows that the largest modality effects are found for the final serial position and that the magnitude of the modality effect is broadly similar across list lengths and tasks, particularly at LL5–12 where performance is not at ceiling. A Pearson’s correlation, performed to directly compare the magnitude of performance for IFR and ISR at the same list lengths and serial positions, resulted in a highly significant, strong, positive correlation, *r*(44) = 0.709, *p* < .001. This indicates that performance in IFR and ISR is highly related across list lengths and serial positions.[Fig-anchor fig7]

Finally we were also interested in comparing magnitude effects across Experiment 1 and 2. Looking across Figures 2 and 7 the magnitude of the modality effect appears to be larger in Experiment 1 but a higher number of inverse modality effects are found for Experiment 2. A Pearson’s correlation was performed to directly compare the magnitude of performance for IFR and ISR at the same list lengths and serial positions across the two experiments. This resulted in a highly significant, strong, positive correlation, *r*(88) = 0.764, *p* < .001. This indicates that performance in Experiment 1 and Experiment 2 is highly similar for IFR and ISR across list lengths and serial positions.

#### The PFR data

**Figure 8 fig8:**
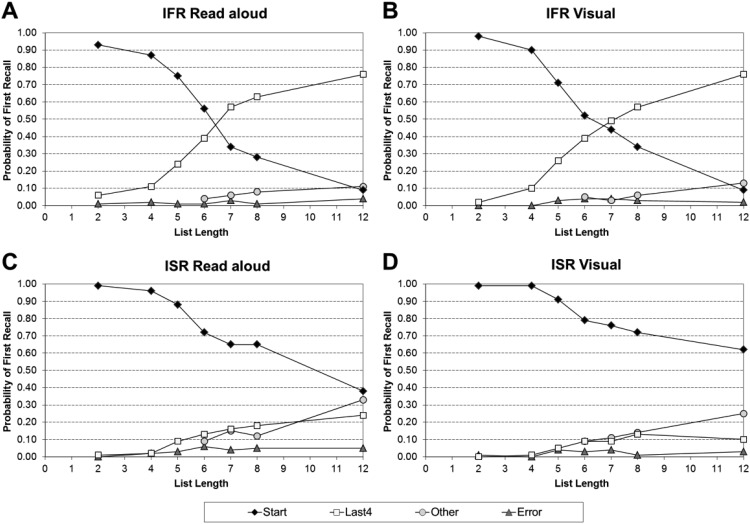
Experiment 2. Probability of first recall (PFR) data for the read aloud and visual IFR and ISR conditions.

The main PFR findings are summarized in Figure 8 (Panel A: read aloud IFR; Panel B: visual IFR; Panel C: read aloud ISR; Panel D: visual ISR). The full values these figures are based on can be found in supplementary Appendix C3 for IFR and supplementary Appendix C4 for ISR. Importantly, the cross-over points (where the modal response changes from “Start” to “Last 4”) look very similar within each task. The cross-over point is between LL6 and LL7 for IFR, but for ISR there is no cross-over point; participants’ modal output is always to start their recall with the first word—regardless of list length.[Fig-anchor fig8]

Table 7 summarizes the findings of a series of two 2 (Task: ISR or IFR) × 2 (Modality: Read Aloud and Visual) × 7 (List Length: 2, 4, 5, 6, 7, 8, and 12) mixed ANOVAs that were performed on the proportion of trials where recall started with Serial Position 1, and the proportion of trials where recall started with one of the last four serial positions. In both analyses, there was a significant main effect of task and a significant main effect of list length. This confirmed that participants were more likely to initiate their recall with: Serial Position 1 in the ISR conditions compared with the IFR conditions, and one of the last four serial positions in the IFR conditions compared with the ISR conditions. The main effect of modality was significant for trials where recall was initiated with Serial Position 1, but nonsignificant for trials where recall was initiated with one of the last four serial positions. Participants were more likely to initiate their recall with Serial Position 1 in the visual condition compared to the read aloud condition.[Table-anchor tbl7]

In both analyses the three-way interactions between task, modality, and list length were nonsignificant, as were the two-way interactions between task and modality, and modality and list length. However, in both analyses, the two-way interactions between task and list length were significant. Simple main effects showed that participants were more likely to initiate their recall with Serial Position 1 in ISR compared with IFR at all list lengths, except LL2 and LL4, and with one of the last four in IFR compared with ISR at all list lengths, except LL2 and LL4. Due to the significant main effect of modality for trials initiated with Serial Position 1, but no interactive effect of modality, there is limited evidence that the modality effect is due to an increased likelihood of starting toward the end of the list with read aloud lists compared to visual lists.

Table 8 shows how far back participants started their recall for the last four items over LL6–12.[Table-anchor tbl8]

A chi-square was performed on this data. Due to the read aloud ISR cell containing fewer than 5 data points, *n* − 3 was grouped with *n* − 2, and *n* − 1 was grouped with *n*. There were significant differences between IFR and ISR in the initial item output: participants were more likely to start with either *n* − 1 or *n*, compared with *n* − 3 or *n* − 2, in IFR than ISR for both read aloud (χ^2^ = 57.9, *p* < .001) and visual (χ^2^ = 17.9, *p* < .001) modalities. These differences reflect the different constraints placed on recall between the two tasks. In ISR, participants are not allowed to recall earlier items and so initiating recall with one of the last four items in ISR reflects suboptimal performance.

However, more importantly, there were no significant differences in how far back participants started in the read aloud compared with visual conditions for either IFR (χ^2^ = 0.877, *p* = .349), or ISR (χ^2^ = 1.79, *p* = .181), confirming that there is little evidence that the modality effect was due to participants starting further back from the end of the list in the read aloud condition, compared to the visual condition, for either ISR or IFR.

#### The effect of the first word recalled on the resultant serial position curves

**Figure 9 fig9:**
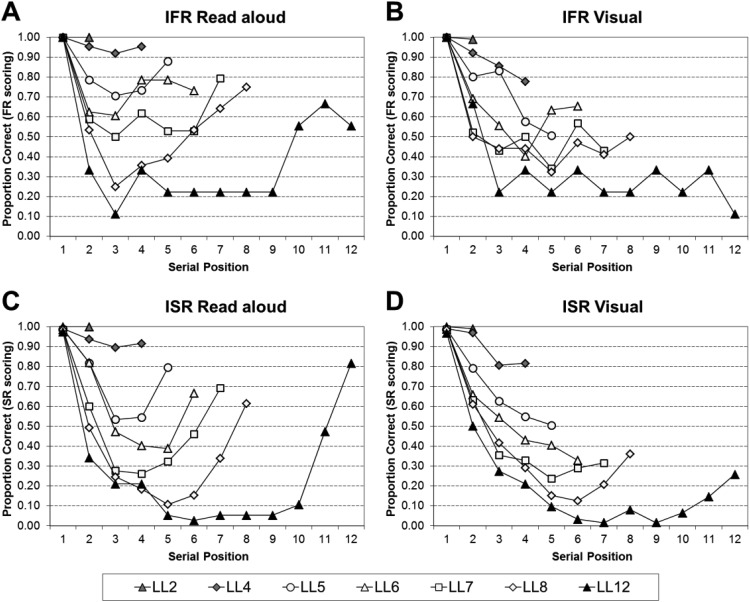
Experiment 2. Resultant serial position curves for trials that began with Serial Position 1 for the read aloud and visual IFR conditions (using FR soring) and ISR conditions (using SR scoring).

Figure 9 shows the resultant serial position curves given recall was initiated with Serial Position 1 for IFR read aloud using FR scoring (Panel A), IFR visual using FR scoring (Panel B), ISR read aloud using SR scoring (Panel C), and ISR visual using SR scoring (Panel D). For both IFR and ISR when recall is initiated with Serial Position 1 the resulting recall shows elevated primacy levels and extended recency in the read aloud conditions, but little to no recency in the visual conditions. Thus, it appears as though read aloud recency items may be less susceptible to output interference compared with the visual recency items.[Fig-anchor fig9]

The resultant serial position curves were analyzed by a series of 2 (Task: ISR or IFR) × 2 (Modality: Read Aloud and Visual) × *n* − 1 (Serial Position: 2 − *n*, where *n* is the list length) mixed ANOVAs. Full statistical analyses for each list length can be found in supplementary Appendix B17 for trials in which recall was initiated with Serial Position 1. To summarize the main points, all of the main effects of task, and the two-way interactions between task and modality were nonsignificant. Thus, given that recall was initiated with Serial Position 1 performance in the tasks does not differ significantly, nor does performance differ significantly as a function of modality and task.

The statistical analyses were then further broken down by task into a series of 2 (Modality: Read Aloud and Visual) × *n* (Serial Position: 2 − *n*, where *n* is the list length) within-subject ANOVAs; full statistical analyses for each list length can be found in supplementary Appendix B18 for IFR using FR scoring, supplementary Appendix B19 for ISR using FR scoring, and supplementary Appendix B20 for ISR using SR scoring. Table 9 visually simplifies the significant findings from supplementary Appendixes B18–B20. The most important finding is that the modality effect is still present in the resultant serial position curves when recall was initiated with Serial Position 1, although this is attenuated for IFR. There were a few cases where the apparent inversion in the modality effect at prerecency serial positions did reach significance (LL5, 7, and 12).[Table-anchor tbl9]

**Figure 10 fig10:**
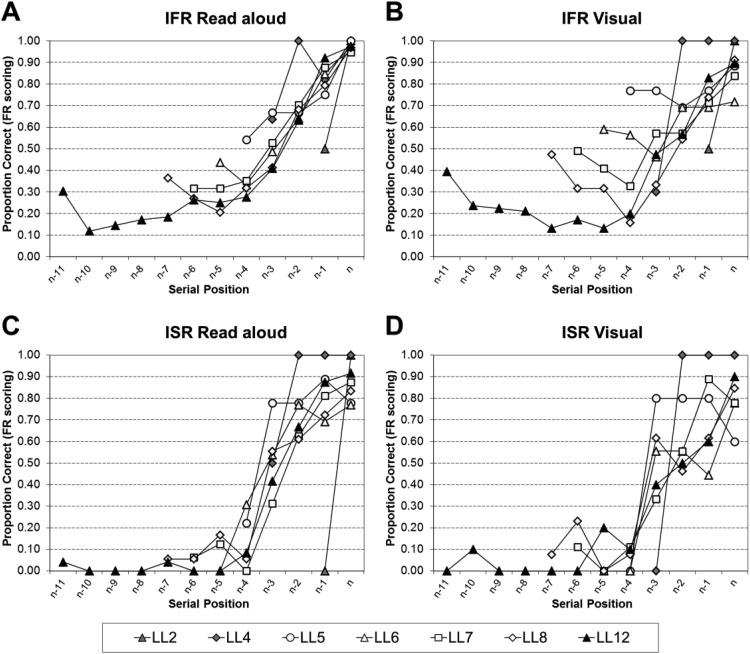
Experiment 2. Resultant serial position curves for trials that began with one of the last four words for the read aloud and visual IFR conditions (using FR soring) and ISR conditions (using FR scoring).

Turning now to the resultant serial position curves given recall was initiated with one of the last four words; Figure 10 shows the resultant serial position curves given recall was initiated with any one of the last four words for IFR read aloud (Panel A), IFR visual (Panel B), ISR read aloud (Panel C), and ISR visual (Panel D). For both IFR and ISR when recall is initiated with one of the last four words the resulting recall shows reduced primacy and extended recency in both the read aloud and visual conditions. It appears as though the modality effect has been greatly reduced/eliminated at most list lengths.[Fig-anchor fig10]

The resultant serial position curves were analyzed by a series of 2 (Task: ISR or IFR) × 2 (Modality: Read Aloud and Visual) × *n* (Serial Position, where *n* is the list length) mixed ANOVAs. Full statistical analyses for trials in which recall was initiated with one of the last four words, for each list length, can be found in supplementary Appendix B21 for all serial positions, and in supplementary Appendix B22 limited to only the final four serial positions. Supplementary Appendix B22 was included as due to the nature of the forward ordered constraint on recall in ISR the comparison in supplementary Appendix B21 produced task differences that were due to the constraints on recall. In summary, the main point to highlight is that when limited to the final four serial positions only, the only significant findings were for the main effect of serial position at LL7 onward. Thus, given that recall was initiated with one of the last four words, all task differences and all modality effects disappear.

The statistical analyses were then further broken down by task into a series of 2 (Modality: Read Aloud and Visual) × *n* (Serial Position, where *n* is the list length) within-subject ANOVAs; full statistical analyses for each list length can be found in supplementary Appendix B23 for the IFR data using FR scoring, and supplementary Appendix B24 for the ISR data using FR scoring. Table 10 visually simplifies the significant findings from supplementary Appendixes B23–B24. The ISR LL4 and LL5 ANOVAs are missing as no participants contributed to these analyses. The most important finding is that the modality effect is greatly reduced/eliminated in the resultant serial position curves when recall was initiated with one of the last four words. Therefore, it appears that the recency advantage for read aloud presented items does not come from trials in which participants initiated their recall with the end items.[Table-anchor tbl10]

### Discussion

This experiment examined modality effects by comparing visual silent instructions with read aloud instructions on performance in IFR and ISR. As in Experiment 1, we were specifically interested in (a) the role of list length in determining the magnitude and extent of the modality effect, (b) the effects of modality and list length in determining the serial position of the item with which participants initiated their recall, and (c) the resultant effect this has on performance in explaining modality effects. We were also interested in whether inverse modality effects at prerecency positions would be more or less frequently observed with the read aloud condition.

The main finding from this experiment was that the modality effects in IFR and ISR are again relatively similar when equivalent methodologies were used. The modality effects for both IFR and ISR were large in magnitude and limited to the final serial position, showing an “ISR-like” modality effect, for short lists (Conrad & Hull, 1968). However, the modality effects for both tasks were smaller and often extended over a greater number of later serial positions, showing an “IFR-like” modality effect, for longer lists (Murdock & Walker, 1969). However, it should also be noted that at the longest lists (LL8 and LL12) the modality effect in IFR was somewhat limited compared with ISR.

The experiment examined the same three possible loci of the modality effect as in Experiment 1: (a) increased tendency to initiate recall with one of the last four items in the read aloud condition compared with the visual condition; (b) more extended terminal runs of items with read aloud rather than visual items (Murdock & Walker, 1969; Nilsson et al., 1975); and (c) greater resilience to output interference of auditory words relative to visual words (Beaman & Morton, 2000; Cowan et al., 2004; Cowan et al., 2002; Craik, 1969; Nilsson et al., 1979).

We found only limited evidence that modality of presentation affected the tendency to initiate recall with the first word. Specifically, participants were significantly less likely to initiate their recall with the first word in the read aloud condition compared to the visual condition. However, there was a nonsignificant effect of modality in the proportion of trials where recall started with one of the last four words. Thus, participants in the read aloud condition did not show any greater tendency to initiate recall toward the end of the list than in the visual condition.

There was little or no evidence that participants started their recall further from the end of the list to give more extended terminal runs of items with read aloud rather than visual items. Rather, there was clear evidence of similar, extended recency in the probability of first recall data for both modalities.

As in Experiment 1, the clearest evidence of modality effects occurred on trials in which participants initiated recall with the first word in the list. On these trials, there were clear auditory recall advantages for items at the end of the list. By contrast, when participants initiated recall with one of the last few words in the list, the modality effect was greatly reduced or eliminated for both tasks. Thus, it appears the presence of the modality effect in both tasks may be related to the greater resilience to output interference of read aloud words relative to visual words (Beaman & Morton, 2000; Cowan et al., 2004; Cowan et al., 2002; Craik, 1969; Nilsson et al., 1979).

Finally, we again observed inverse modality effects at the early to middle serial positions at longer list lengths using the read aloud methodology, but these appear to be more frequent for ISR than IFR, perhaps reflecting a greater importance on rehearsal in ISR than IFR for maintaining sequential order (Macken et al., 2016).

## Comparison of Experiment 1 and Experiment 2

We performed a set of analyses comparing the auditory condition of Experiment 1 with the read aloud condition of Experiment 2. There are differences between these two “auditory” methods, most obviously in the voices in which the auditory stimuli are heard, that could potentially result in different findings. In addition, one advantage of using the read aloud method is that there is no question as to whether participants have misheard the item. By contrast, an advantage of using the auditory method is that the stimuli are passively presented to the participants, and so (as in the visual condition) participants are free to perform whatever additional encoding (e.g., imagery, rehearsal) they wish while stimuli are being presented. The full set of analyses can be found in Appendix D of the supplementary material, but the main conclusion is that there are very few, if any, significant differences between the auditory and read aloud conditions within the serial position curves, PFR, and resultant serial position curves.

## General Discussion

Modality effects have previously been shown to be different in magnitude and extent in IFR and ISR. The modality effect in ISR is often large but limited to the final list item, whereas the modality effect in IFR is often smaller but spread over multiple recency positions (Conrad & Hull, 1968; Corballis, 1966; Craik, 1969; Engle, 1974; Murdock & Walker, 1969; Penney, 1975, 1989; Watkins & Watkins, 1973, 1980; Watkins et al., 1974). In line with our earlier work that has shown that the findings in IFR and ISR are more similar when the two tasks are compared at similar list lengths, we have shown that these empirical differences in the magnitude and extent of the modality effects between the tasks may instead be attributable to differences in the list lengths that are typically used in the two tasks. Specifically, we found that “ISR-like” modality effects that were large in magnitude and limited in extent to single items in both tasks at short list lengths (more typical of ISR) and “IFR-like” modality effects that were smaller in magnitude and more extended to multiple items in both tasks at longer list lengths (more typical of IFR).

Across two experiments, this research has demonstrated that the modality effect in both tasks appear to be related to the greater resilience to output interference of auditory (Experiment 1) and read aloud (Experiment 2) words relative to silently read visual words (Beaman & Morton, 2000; Cowan et al., 2004; Cowan et al., 2002; Craik, 1969; Nilsson et al., 1979). These substantial similarities in performance between IFR and ISR have provided further evidence for the need for greater theoretical integration between IFR and ISR. The finding that observed differences in the effects of immediate memory variables on IFR and ISR may be related to the differences in the list lengths that are used rather than the task is consistent with a growing body of work showing reduced differences in rehearsal, presentation rate, word length, and articulatory suppression (Bhatarah, Ward, Smith, & Hayes, 2009), phonological similarity (Spurgeon et al., 2014a) and knowledge of list length and test expectancy (Grenfell-Essam & Ward, 2012) when the two tasks are examined using identical methodologies.

Our finding that broadly similar modality effects are observed in IFR and ISR supports the notion that there may be common mechanisms underpinning modality effects in the two tasks. Currently, the most influential account, the phonological loop account of working memory (Baddeley, 1986) addresses the three-way interactions between modality, articulatory suppression and variables such as word length and phonological similarity, but considers modality effects to be outside the scope of the model, while the leading account of the modality effect (the Feature model, Nairne, 1988, 1990; Neath, 2000) addresses both serial position and modality effects, but at present is limited to explaining only ISR. Alternative models that could in principle explain modality effects across a wide range of tasks do exist. They tend to concentrate on the capacity of working memory (Cowan, 2001), the role of attention and memory (Cowan, 1988, 1998), and the interplay between attention, perception and action (Jones, 1993; Jones, Macken, & Nicholls, 2004; Jones et al., 2006; Macken et al., 2016) but tend to focus on serial recall, and do not provide detailed explanations of serial position curves and output order in the two tasks.

Of the four formal integrated accounts of IFR and ISR identified in the introduction (Anderson et al., 1998; Brown et al., 2007; Farrell, 2012; Grossberg & Pearson, 2008), only the latter’s LIST-PARSE model currently offers an account of the modality effect, and in common with the account of Cowan (1998), this explanation relies upon an auditory sensory store, not so different from the PAS account of Crowder and Morton (1969). Within our data sets, we used silent written recall, such that auditory features of most recently presented study lists items may still be available at different points at output throughout recall. One issue with the LIST-PARSE model is that it assumes different patterns of rehearsal in the two tasks. Although this may at first seem reasonable, Bhatarah et al. (2009) have shown that the patterns of rehearsals in the two tasks are very similar when the list lengths are equated.

Considering the three other accounts, Anderson et al. offer an integrated theory of list memory, but the specific simulations for IFR and ISR assume rather different representational structures. When simulating serial recall, Anderson, Bothell, Lebiere, and Matessa (1998) propose a hierarchically organized chunk structure with the List chunks associated with subordinate group chunks which are in turn associated with individual element chunks each representing items at specific serial positions. By contrast, in free recall, Anderson et al. (1998) modify a STM buffer model such that about four items are actively maintained through rehearsal; there is no hierarchical organization, such that it is a moot point as to whether the accounts of IFR and ISR are truly integrated into a set of common memory mechanisms. The Brown et al. (2007) SIMPLE model does not currently account for modality effects, but it does assume that items are represented within a multidimensional space, and so the model could be developed such that items were represented with modality-dependent and modality-independent features, such as in the feature model (Nairne, 1988, 1990; Neath, 2000; Neath & Nairne, 1995) such that it could be well placed to model modality effects. Finally, Farrell (2012) also assumes a hierarchical list structure with individual list items assumed to be associated with group nodes and the different group nodes associated with lists. Farrell’s (2012) model assumes that participants must access individual items by first retrieving their group node. Recalling the most recent or current group is assumed to be straightforward, but retrieving other groups is more difficult. Once a group node has been accessed, it is assumed that recall will proceed in a forward ordered manner. The model simulates the change in PFR with increasing list length (Ward et al., 2010), and correctly predicts that the first recall will affect the resultant serial position curve. The model has some success in modeling grouping effects in IFR and ISR of grouped and ungrouped lists of different list lengths (Spurgeon et al., 2015). Although the model focuses on simulating aspects of recall other than modality, the use of groups is potentially beneficial, as it is known that the effects of grouping manipulations are more exaggerated in the auditory than the visual modality (Frankish, 1985), and there is some evidence that later auditory groups are easier to access than the corresponding visual groups in forward-ordered recall (Metcalfe & Sharpe, 1985).

Our data constrain different possible unified accounts of IFR and ISR. The finding that the PFR is unaffected by the modality of presentation suggests that the relative accessibilities of the different visual items at test is equivalent to the relative accessibilities of the different auditory items. This might be the case if the differences between visual and auditory items apply to all the list items, but this might not be expected to be the case if the last one or more auditory (but not visual) items are assumed to be particularly highly accessible relative to the earlier list items owing to additional source of highly accessible items or particularly well preserved sets of features.

A second finding that may constrain unified accounts of modality effects is the finding that the extent and magnitude of the modality effects are affected by the list length in both IFR and ISR. Our data show that the modality effects in both IFR and ISR are large and limited to the last word on short lists, but smaller in magnitude but extending over several end of list items in longer lists. We believe that a grouping account of modality effects may offer one solution. If we assume that participants parse lists of short lengths into one group, but longer lists into multiple groups of varying sizes (Farrell, 2012; Spurgeon et al., 2015), then for short lists, the group boundaries consistently fall at the end of the list, but for longer lists with multiple groups, the group boundaries are more varied, and are spread across several end of list positions. If we assume accessibility to later cued groups is superior for auditory groups (Metcalfe & Sharpe, 1985) and/or the effects of grouping are more exaggerated with auditory lists (Frankish, 1985) then auditory advantages at group boundaries may be expected to be distributed across several list positions, leading to a smaller but more extended modality advantage.

A third feature of our data that may constrain unified accounts of modality effects is that the magnitude of modality differences is greatest when participants initiate their recall from the start of the list. This suggests that a major factor in determining the modality effect in both IFR and ISR is the greater resistance to output interference observed with auditory stimuli (including visual words that are read aloud) relative to words in the visual silent presentation condition (Cowan et al., 2002, 2004). One explanation for the resilience of auditory items within our data sets is that auditory words are stored with more enriched sets of features than visual items (e.g., Nairne, 1990; Neath, 2000), and that recalled list items interfere with the recall of as yet to-be-remembered items. If we further assume that the internalized voice that accompanies silent written recall at output is more similar to the internalized voice that accompanies covert phonological recoding in the visual silent presentation condition, then as recall progresses the differences in the effect of subsequent recalls (output interference) on visual relative to auditory yet-to-be-recalled items will increase.

A fourth and final feature of our data that may constrain unified accounts of IFR and ISR is the confirmation of inverse modality effects (Beaman, 2002; Macken et al., 2016): visual recall advantages that occur in early mid serial positions with medium to long list lengths. Consistent with Macken et al. (2016), we assume that the modality of presentation affects the way that *all* the list items are processed. We agree that presenting stimuli in the visual silent conditions may be more conducive to cumulative forward-ordered rehearsal than visual read aloud as the former but not the latter is unimpeded by the need to articulate or vocalize each currently presented item. We are also willing to accept that participants might either feel a greater necessity to rehearse the visual silent items relative to the words in the auditory condition in order to form groups or subsequences of lists and/or be less able to incorporate the current auditory presented word into a cumulatively expanding rehearsal set. The benefits of cumulative forward-ordered rehearsal may be greatest not at the start of the list but at early mid list positions (see Grenfell-Essam et al., 2013), and the benefit may be greatest at longer list lengths, where there might be both greater rehearsal opportunities and where the difficulty in recalling unrehearsed early list items might be more difficult (see Ward, 2002). Currently, many contemporary accounts of IFR and ISR (e.g., the SIMPLE model, Brown et al., 2007; the feature model, e.g., Nairne, 1990; Neath, 2000; and the Farrell, 2012 model) do not incorporate an account of rehearsal. However, our data suggest that recall of read aloud stimuli is reasonably similar to the recall of auditory stimuli, and we do not find the lowered levels of overall performance observed in the read aloud conditions of Macken et al. (2016). Of course, there are many differences between the methodologies used in our study and that of Macken et al. (2016). Most notably, we used different tasks, an experiment-unique set of stimuli, words rather than consonants and a rate of presentation of one word per second that was slightly slower than that standardly used by Macken et al. (2016; 750 ms per word).

Our preferred explanation of our data is that the modality of presentation affects the processing of the entire list of items. We assume that longer lists are parsed into multiple groups (of varying sizes), and the presentation of each auditory (or read aloud) item leads to a set of more richly encoded features than those encoded following the presentation of each visual silent item. The modality of presentation does not affect the PFR, as each item within the same modality in the list is encoded with the same richness of features. However, the encoding of relatively impoverished written recalled items will create greater relative interference for the impoverished visual items than the richly encoded auditory items, leading to improved recall of auditory over visual items on later recalls. We finally assume that participants might rehearse in a cumulative forward-ordered manner with all lists, but they may be able to rehearse longer sequences of early lists items with visual silent lists than with either auditory or read aloud lists.

In summary, we have reported a systematic exploration of the modality effects across a wide range of list lengths in both IFR and ISR, contrasting visual silent presentation with both auditory (Experiment 1) and read aloud (Experiment 2) presentation. We have shown that the magnitude and extent of modality effects are similar across tasks when the list length is controlled, and we have also shown inverse modality effects in both tasks in early mid list positions for medium to long lists. We believe these findings encourage attempts to provide a unified account of both IFR and ISR. We believe that in both tasks, modality effects arise through the greater resilience of auditory items to output interference. Our data provide four findings that will constrain the development of such a theory, and additionally provide a rich data set that could be used to model the development of the serial position curve with each successively presented list item.

## Supplementary Material

10.1037/xlm0000430.supp

## Figures and Tables

**Table 1 tbl1:** Experiment 1: Summary of the ANOVA Analyses Conducted on the Overall Serial Position Curves

List length	Task	Scoring	Main effects		Two-way interaction	
			Modality	Serial position	Modality × Serial position	Simple main effects (Serial position)
2	IFR	FR	x	x	x	—
	ISR	FR	x	x	x	—
	ISR	SR	x	x	x	—
4	IFR	FR	x	x	x	4
	ISR	FR	x	x	✓	4
	ISR	SR	x	✓	✓	4
5	IFR	FR	x	✓	✓	5
	ISR	FR	x	✓	✓	5
	ISR	SR	x	✓	✓	5
6	IFR	FR	✓	✓	✓	5, 6
	ISR	FR	✓	✓	✓	5, 6
	ISR	SR	✓	✓	✓	5, 6
7	IFR	FR	x	✓	x	7
	ISR	FR	✓	✓	✓	6, 7
	ISR	SR	x	✓	✓	6, 7
8	IFR	FR	x	✓	✓	4, 6, 7, 8
	ISR	FR	x	✓	✓	7, 8
	ISR	SR	x	✓	✓	7, 8
12	IFR	FR	✓	✓	x	11, 12
	ISR	FR	✓	✓	✓	2, 4, 5, 10, 11, 12
	ISR	SR	✓	✓	✓	11, 12
*Note*. ✓ indicates a significant finding and x indicates a nonsignificant finding. The final column displays which serial positions showed a significant advantage for the auditory condition; underlined values signify a significant inversion of the modality effect.						

**Table 2 tbl2:** Experiment 1: Summary of the ANOVA Analyses

Variable	*df*	*MSE*	*F*	η_*p*_^2^	*p*
Probability of first recall = Serial Position 1					
Task	1, 38	.270	27.7	.421	<.001
Modality	1, 38	.085	.068	.002	.795
List length	6, 228	.046	88.0	.698	<.001
Task × Modality	1, 38	.085	1.15	.029	.289
Task × List Length	6, 228	.046	5.89	.134	<.001
Modality × List Length	6, 228	.028	.572	.015	.752
Task × Modality × List Length	6, 228	.028	1.80	.045	.100
Probability of first recall = Last four					
Task	1, 32	.241	20.5	.391	<.001
Modality	1, 32	.083	.159	.005	.692
List length	6, 192	.045	34.6	.519	<.001
Task × Modality	1, 32	.083	2.14	.063	.153
Task × List Length	6, 192	.045	4.76	.130	<.001
Modality × List Length	6, 192	.029	.492	.015	.814
Task × Modality × List Length	6, 192	.029	2.14	.063	.051

**Table 3 tbl3:** Experiment 1: Summary of the Probability of First Recall Data Showing the Frequency of Trials That Commenced With Either n − 3, n − 2, n − 1, or n for LL6–12

Task	*n* − 3	*n* − 2	*n* − 1	*n*
ISR Auditory	24	22	16	0
ISR Visual	22	11	11	8
ISR Subtotal	**46**	**33**	**27**	**8**
IFR Auditory	18	38	50	68
IFR Visual	32	45	67	68
IFR Subtotal	**50**	**83**	**117**	**136**
Total	96	116	144	144
*Note.* values in bold represent the task subtotal.				

**Table 4 tbl4:** Experiment 1: Summary of the ANOVA Analyses Conducted on the Resultant Serial Position Curves Given Recall was Initiated With Serial Position 1

List length	Task	Scoring	Main effects		Two-way interaction	
			Modality	Serial position	Modality × Serial position	Simple main effects (Serial position)
4	IFR	FR	x	x	x	4
	ISR	FR	✓	x	x	4
	ISR	SR	x	x	x	4
5	IFR	FR	x	x	✓	5
	ISR	FR	✓	✓	✓	5
	ISR	SR	x	✓	✓	3, 5
6	IFR	FR	x	x	✓	6
	ISR	FR	✓	✓	✓	2, 6
	ISR	SR	✓	✓	✓	6
7	IFR	FR	x	x	x	6, 7
	ISR	FR	x	✓	✓	6, 7
	ISR	SR	x	✓	✓	6, 7
8	IFR	FR	x	x	✓	3, 4, 7, 8
	ISR	FR	x	✓	✓	8
	ISR	SR	x	✓	✓	7, 8
12	IFR	FR	x	x	✓	9, 11, 12
	ISR	FR	x	✓	✓	11, 12
	ISR	SR	✓	✓	✓	11, 12
*Note*. ✓ indicates a significant finding and x indicates a nonsignificant finding. The final column displays which serial positions showed a significant advantage for the auditory condition; underlined values signify a significant inversion of the modality effect.						

**Table 5 tbl5:** Experiment 1: Summary of the ANOVA Analyses Conducted on the Resultant Serial Position Curves Given Recall was Initiated With One of the Last Four Words

List length	Task	Scoring	Main effects		Two-way interaction	
			Modality	Serial position	Modality × Serial position	Simple main effects (Serial position)
4	IFR	FR	x	x	x	—
	ISR	FR	—	—	—	—
5	IFR	FR	x	x	x	—
	ISR	FR	x	x	x	—
6	IFR	FR	x	x	x	6
	ISR	FR	x	x	x	—
7	IFR	FR	x	✓	x	—
	ISR	FR	x	✓	x	—
8	IFR	FR	x	✓	x	—
	ISR	FR	x	✓	x	6
12	IFR	FR	x	✓	x	—
	ISR	FR	x	✓	x	—
*Note*. ✓ indicates a significant finding and x indicates a nonsignificant finding. The final column displays which serial positions showed a significant advantage for the auditory condition.						

**Table 6 tbl6:** Experiment 2: Summary of the ANOVA Analyses Conducted on the Overall Serial Position Curves

List length	Task	Scoring	Main effects		Two-way interaction	
			Modality	Serial position	Modality × Serial position	Simple main effects (Serial position)
2	IFR	FR	x	x	x	—
	ISR	FR	x	x	x	—
	ISR	SR	x	x	x	—
4	IFR	FR	x	x	x	4
	ISR	FR	x	x	x	4
	ISR	SR	x	✓	✓	3, 4
5	IFR	FR	x	✓	✓	4, 5
	ISR	FR	x	✓	✓	5
	ISR	SR	x	✓	✓	5
6	IFR	FR	x	✓	✓	4, 5, 6
	ISR	FR	✓	✓	✓	6
	ISR	SR	x	✓	✓	6
7	IFR	FR	✓	✓	x	5, 7
	ISR	FR	x	✓	✓	6, 7
	ISR	SR	x	✓	✓	6, 7
8	IFR	FR	x	✓	x	−
	ISR	FR	x	✓	✓	2, 3, 7, 8
	ISR	SR	x	✓	✓	2, 3, 7, 8
12	IFR	FR	x	✓	✓	2, 8, 12
	ISR	FR	x	✓	✓	1, 2, 3, 4, 9, 10, 11, 12
	ISR	SR	x	✓	✓	1, 2, 3, 10, 11, 12
*Note*. ✓ indicates a significant finding and x indicates a nonsignificant finding. The final column displays which serial positions showed a significant advantage for the auditory condition; underlined values signify a significant inversion of the modality effect.						

**Table 7 tbl7:** Experiment 2: Summary of the ANOVA Analyses

Variable	*df*	*MSE*	*F*	η_*p*_^2^	*p*
Probability of first recall = Serial Position 1					
Task	1, 38	.266	27.4	.419	<.001
Modality	1, 38	.070	5.03	.117	.031
List length	6, 228	.055	87.7	.698	<.001
Task × Modality	1, 38	.070	1.48	.038	.231
Task × List Length	6, 228	.055	7.48	.164	<.001
Modality × List Length	6, 228	.028	1.62	.041	.141
Task × Modality × List Length	6, 228	.028	1.69	.043	.125
Probability of first recall = Last four					
Task	1, 35	.226	42.4	.548	<.001
Modality	1, 35	.079	2.93	.077	.096
List length	6, 210	.048	44.7	.561	<.001
Task × Modality	1, 35	.079	.534	.015	.470
Task × List Length	6, 210	.048	14.0	.285	<.001
Modality × List Length	6, 210	.027	.660	.019	.682
Task × Modality × List Length	6, 210	.027	.719	.020	.635

**Table 8 tbl8:** Experiment 2: Summary of the PFR Data Showing the Frequency of Trials That Commenced With Either n − 3, n − 2, n − 1, or n for LL6–12

Task	*n* − 3	*n* − 2	*n* − 1	*n*
ISR Read aloud	26	29	12	4
ISR Visual	18	9	7	7
ISR Subtotal	**44**	**38**	**19**	**11**
IFR Read aloud	15	49	84	87
IFR Visual	36	33	72	80
IFR Subtotal	**51**	**82**	**156**	**167**
Total	95	120	175	178
*Note.* values in bold represent the task subtotal.				

**Table 9 tbl9:** Experiment 2: Summary of the ANOVA Analyses Conducted on the Resultant Serial Position Curves Given Recall was Initiated With Serial Position 1

List length	Task	Scoring	Main effects		Two-way interaction	
			Modality	Serial position	Modality × Serial position	Simple main effects (Serial position)
4	IFR	FR	x	x	x	4
	ISR	FR	x	✓	x	4
	ISR	SR	x	✓	✓	3, 4
5	IFR	FR	x	x	✓	3, 4, 5
	ISR	FR	x	x	x	5
	ISR	SR	x	✓	✓	5
6	IFR	FR	x	x	x	4
	ISR	FR	x	✓	x	6
	ISR	SR	x	✓	x	6
7	IFR	FR	x	x	x	7
	ISR	FR	x	✓	✓	3, 6, 7
	ISR	SR	x	✓	✓	6, 7
8	IFR	FR	x	✓	x	—
	ISR	FR	x	✓	✓	7, 8
	ISR	SR	x	✓	✓	8
12	IFR	FR	x	x	x	10
	ISR	FR	x	✓	✓	2, 11, 12
	ISR	SR	x	✓	✓	2, 11, 12
*Note*. ✓ indicates a significant finding and x indicates a nonsignificant finding. The final column displays which serial positions showed a significant advantage for the auditory condition; underlined values signify a significant inversion of the modality effect.						

**Table 10 tbl10:** Experiment 2: Summary of the ANOVA Analyses Conducted on the Resultant Serial Position Curves Given Recall was Initiated With One of the Last Four Words

List length	Task	Scoring	Main effects		Two-way interaction	
			Modality	Serial position	Modality × Serial position	Simple main effects (Serial position)
4	IFR	FR	x	x	x	3
	ISR	FR	—	—	—	—
5	IFR	FR	x	x	x	—
	ISR	FR	—	—	—	—
6	IFR	FR	x	✓	x	6
	ISR	FR	x	✓	x	5
7	IFR	FR	x	✓	x	1, 5
	ISR	FR	x	✓	x	—
8	IFR	FR	x	✓	x	3
	ISR	FR	x	✓	x	3
12	IFR	FR	x	✓	x	—
	ISR	FR	x	✓	x	—
*Note*. ✓ indicates a significant finding and x indicates a nonsignificant finding. The final column displays which serial positions showed a significant advantage for the auditory condition; underlined values signify a significant inversion of the modality effect.						

## Footnotes

1 We would like to thank Ian Neath for this suggestion.
