# Supplementary material for: Common Modality Effects in Immediate Free Recall and Immediate Serial Recall
Source: J Exp Psychol Learn Mem Cogn. 2017 May 29;43(12):1909–33. doi: 10.1037/xlm0000430 (PMC5729966; doi:10.1037/xlm0000430)
Supplement: Supplementary file 1 [file zfv999173543so1.docx]

**Supplemental Materials**

**Common Modality Effects in Immediate Free Recall and Immediate Serial Recall**

**by R. Grenfell-Essam et al., 2017, *Journal of Experimental Psychology: Learning, Memory, and Cognition***

**http://dx.doi.org/10.1037/xlm0000430**

This supplementary material document contains Appendices A-D which provide integral tables and analyses.

**Supplementary Appendix A: Additional analyses examining the effect of block order on the extent and magnitude of the modality effects**

Experiments 1 and 2 both used a counterbalanced blocked design where half the participants received first a block of visual trials followed by a second block of auditory trials, and the other half received blocks of trials with the order of the presentation modality reversed.

This set of supplementary analyses presents a breakdown of immediate free recall (IFR) and immediate serial recall (ISR) in Experiment 1 and Experiment 2 examining whether the block order affects performance within each task and each modality. We will consider Experiment 1 and 2 in turn. For both experiments we will present the figures and ANOVAs for serial position curves (SPCs) and the probability of first recall (PFR) analyses.

**Experiment 1**

**Analyses of the serial position curves of all the data.** A series of four figures below compare the block 1 and block 2 performance for IFR auditory (Figure A1), IFR visual (Figure A2), ISR auditory (Figure A3), ISR visual (Figure A4) for Experiment 1. IFR tasks are scored with FR scoring, ISR tasks are scored with SR scoring. All figures show very similar performance indicating that the order in which they experienced the modalities did not greatly affect recall.

-----------------------------------

--Figures A1-A4 about here—

------------------------------------

The serial position curves were analysed separately for each task by a series of 2 (block: 1 or 2) x *n* (serial position, where *n* is the list length) mixed ANOVAs; full statistical analyses for each list length can be found in Table A1 (IFR) and Table A2 (ISR). To summarise these findings, there were no significant main effects of block order and the majority of interactions between block and serial position were also not significant. The one significant interaction, ISR auditory list length 8, was driven by the higher recall in block 2 for SP6 only (p = .023, all other p’s > 0.05). Therefore, for both tasks and both modalities there was very little difference in recall based on block.

-----------------------------------

--Tables A1-A2 about here—

------------------------------------

**PFR.** Figure A5 compares likelihood of initiating recall with one the four PFR categories (start, last four, other and error) for block 1 and block 2 for both tasks. The PFR categories are split over two panels to improve clarity. These figures do show variability indicating that the order in which they experienced the modalities may affect where participants choose to initiate their recall. It appears participants are less likely to initiate their recall with the first item in auditory trials in block 2 for both IFR and ISR but instead are more likely to initiate their recall with one of the last four words. The opposite is true for IFR visual block 2 where participants instead are more likely to initiate their recall with the first item and less likely to initiate their recall with one of the last four items. The visual ISR data seems more similar across blocks 1 and 2.

-----------------------------------

--Figure A5 about here—

------------------------------------

Table A3 summarises the findings of a series of 2 (block: 1 or 2) x 7 (list length: 2, 4, 5, 6, 7, 8, and 12) mixed ANOVAs that were performed on the proportion of trials where recall started with SP1, and the proportion of trials where recall started with one of the last four serial positions for both IFR and ISR. The main effect for block was significant for IFR auditory when PFR=SP1 and when PFR=Last4. IFR auditory participants were more likely to initiate their recall with the first word in block 1 and more likely to initiate their recall with one of the last four words word in block 2. The IFR visual PFR=Last4 interaction was driven by the higher likelihood of initiating recall with one of the last four words in block 1 for LL6 only (p = 0.007, all other p’s > 0.05). The ISR auditory PFR=Last4 interaction was driven by the higher likelihood of initiating recall with one of the last four words in block 2 for LL8 only (p = 0.024, all other p’s > 0.05). Therefore, the order of the modalities does appear to affect the likelihood of initiating recall. Importantly though the overall SPC analysis indicates that these differences in recall initiation point do not affect the modality effect overall.

-----------------------------------

--Table A3 about here—

------------------------------------

**Experiment 2**

**Analyses of the serial position curves of all the data.**. A series of four figures below compare the block 1 and block 2 performance for IFR read aloud (Figure A6), IFR visual (Figure A7), ISR read aloud (Figure A8), ISR visual (Figure A9) for Experiment 2. The IFR tasks are scored with FR scoring, and the ISR tasks are scored with SR scoring. All figures show similar performance for block 1 and 2, indicating that the order in which participants experienced the modalities did not appear to greatly affect recall.

-----------------------------------

--Figures A6-A9 about here—

------------------------------------

The serial position curves were analysed separately for each task by a series of 2 (block: 1 or 2) x *n* (serial position, where *n* is the list length) mixed ANOVAs; full statistical analyses for each list length can be found in Table A4 (IFR) and Table A5 (ISR). To summarise these findings, there were no significant main effects of block and the majority of interactions between block and serial position were also not significant. The two significant interactions occurred in the read aloud modality. The IFR read aloud list length 8 interaction was driven by the higher recall in block 2 for SP2 (p = .013) and SP6 (p = .024) (all other p’s > 0.05). The ISR read aloud list length 12 interaction was driven by the higher recall in block 1 for SP2 only (p = .038, all other p’s > 0.05). Therefore, for both tasks and both modalities there was very little difference in recall based on block.

-----------------------------------

--Tables A4-A5 about here—

------------------------------------

**PFR.** Figure A10 compares likelihood of initiating recall with one the four PFR categories for block 1 and block 2 for both tasks. The PFR categories are split over two panels to improve clarity. These figures do show variability indicating that the order in which they experienced the modalities may affect where participants choose to initiate their recall. For IFR it appears participants are less likely to initiate their recall with the first item in auditory trials in block 2 but instead are more likely to initiate their recall with one of the last four words. For ISR the trends are more similar across the blocks.

-----------------------------------

--Figure A10 about here—

------------------------------------

Table A6 summarises the findings of a series of 2 (block: 1 or 2) x 7 (list length: 2, 4, 5, 6, 7, 8, and 12) mixed ANOVAs that were performed on the proportion of trials where recall started with SP1, and the proportion of trials where recall started with one of the last four serial positions for both IFR and ISR. To summarise these findings, there were no significant main effects of block or significant block and serial position interactions. Therefore, the order of the modalities does not appear to affect the likelihood of initiating recall at the start or end of the list.

-----------------------------------

--Table A6 about here—

------------------------------------

**Table A1.** Experiment 1. Analyses of the SPCs, shown in Figure A1 and A2, using FR scoring for IFR. At each LL, the data were subjected to a 2 (block: 1 or 2) x *n* (SP: 1, …, *n*, where *n* is the LL) mixed ANOVA. The values represent the p-value.

| **List length** | **Task** | **Modality** | **Main Effects** | | **2-way interaction** |
| --- | --- | --- | --- | --- | --- |
|  |  |  | **Block** | **SP** | **Block x SP** |
| 2 | IFR | Auditory | *F* (1, 18) = 1.52, *MSE* = .041,  *p* = .233, *η^2^_p_* = .078 | *F* (1, 18) = 2.88, *MSE* = .006  *p* = .107, *η^2^_p_* = .138 | *F* (1, 18) = 0.72, *MSE* = .006,  *p* = .407, *η^2^_p_* = .038 |
|  | IFR | Visual | *F* (1, 18) = 0.53, *MSE* = .017,  *p* = .476, *η^2^_p_* = .029 | *F* (1, 18) = 1.00, *MSE* = .001  *p* = .331, *η^2^_p_* = .053 | *F* (1, 18) = 1.00, *MSE* = .001  *p* = .331, *η^2^_p_* = .053 |
| 4 | IFR | Auditory | *F* (1, 18) = 0.73, *MSE* = .083,  *p* = .403, *η^2^_p_* = .039 | ***F* (3, 54) = 5.11, *MSE* = .023**  ***p* = .003, *η^2^_p_* = .221** | *F* (3, 54) = 0.72, *MSE* = .023  *p* = .543, *η^2^_p_* = .039 |
|  | IFR | Visual | *F* (1, 18) = 0.10, *MSE* = .081,  *p* = .757, *η^2^_p_* = .005 | *F* (3, 54) = 2.69, *MSE* = .030  *p* = .056, *η^2^_p_* = .130 | *F* (3, 54) = 1.70, *MSE* = .030  *p* = .178, *η^2^_p_* = .086 |
| 5 | IFR | Auditory | *F* (1, 18) = 1.19, *MSE* = .086,  *p* = .291, *η^2^_p_* = .062 | ***F* (4, 72) = 9.46, *MSE* = .043**  ***p* < .001, *η^2^_p_* = .345** | *F* (4, 72) = 1.13, *MSE* = .043  *p* = .351, *η^2^_p_* = .059 |
|  | IFR | Visual | *F* (1, 18) < 0.001, *MSE* = .048,  *p* = 1.000, *η^2^_p_* < .001 | ***F* (4, 72) = 5.77, *MSE* = .052**  ***p* < .001, *η^2^_p_* = .243** | *F* (4, 72) = 0.54, *MSE* = .052  *p* = .708, *η^2^_p_* = .029 |
| 6 | IFR | Auditory | *F* (1, 18) = 0.09, *MSE* = .061,  *p* = .771, *η^2^_p_* = .005 | ***F* (5, 90) = 11.32, *MSE* = .049**  ***p* < .001, *η^2^_p_* = .386** | *F* (5, 90) = 0.73, *MSE* = .049  *p* = .605, *η^2^_p_* = .039 |
|  | IFR | Visual | *F* (1, 18) = 0.07, *MSE* = .074,  *p* = .791, *η^2^_p_* = .004 | *F* (5, 90) = 2.21, *MSE* = .062  *p* = .060, *η^2^_p_* = .109 | *F* (5, 90) = 0.17, *MSE* = .062  *p* = .975, *η^2^_p_* = .009 |
| 7 | IFR | Auditory | *F* (1, 18) = 0.43, *MSE* = .081,  *p* = .523, *η^2^_p_* = .023 | ***F* (6, 108) = 8.49, *MSE* = .057**  ***p* < .001, *η^2^_p_* = .320** | *F* (6, 108) = 0.69, *MSE* = .057  *p* = .657, *η^2^_p_* = .037 |
|  | IFR | Visual | *F* (1, 18) = 0.22, *MSE* = .047,  *p* = .645, *η^2^_p_* = .012 | *F* (6, 108) = 1.46, *MSE* = .078  *p* = .198, *η^2^_p_* = .075 | *F* (6, 108) = 0.57, *MSE* = .078  *p* = .755, *η^2^_p_* = .031 |
| 8 | IFR | Auditory | *F* (1, 18) = 0.11, *MSE* = .037,  *p* = .744, *η^2^_p_* = .006 | ***F* (7, 126) = 22.51, *MSE* = .051**  ***p* < .001, *η^2^_p_* = .556** | *F* (7, 126) = 1.92, *MSE* = .051  *p* = .071, *η^2^_p_* = .096 |
|  | IFR | Visual | *F* (1, 18) = 0.38, *MSE* = .066,  *p* = .546, *η^2^_p_* = .021 | ***F* (7, 126) = 4.58, *MSE* = .050**  ***p* < .001, *η^2^_p_* = .203** | *F* (7, 126) = 1.15, *MSE* = .050  *p* = .337, *η^2^_p_* = .060 |
| 12 | IFR | Auditory | *F* (1, 18) = 1.25, *MSE* = .039,  *p* = .279, *η^2^_p_* = .065 | ***F* (11, 198) = 21.47, *MSE* = .046**  ***p* < .001, *η^2^_p_* = .544** | *F* (11, 198) = 0.75, *MSE* = .046  *p* = .752, *η^2^_p_* = .037 |
|  | IFR | Visual | *F* (1, 18) = 0.63, *MSE* = .060,  *p* = .438, *η^2^_p_* = .034 | ***F* (11, 198) = 10.19, *MSE* = .057**  ***p* < .001, *η^2^_p_* = .361** | *F* (11, 198) = 1.33, *MSE* = .057  *p* = .208, *η^2^_p_* = .069 |

*Note:* Significant main effects and interactions are presented in bold. Bonferroni corrected *p*-level = .0071.

**Table A2.** Experiment 1. Analyses of the SPCs, shown in Figure A3 and A4, using SR scoring for ISR. At each LL, the data were subjected to a 2 (block: 1 or 2) x *n* (SP: 1, …, *n*, where *n* is the LL) mixed ANOVA.

| **List length** | **Task** | **Modality** | **Main Effects** | | **2-way interaction** |
| --- | --- | --- | --- | --- | --- |
|  |  |  | **Block** | **SP** | **Block x SP** |
| 2 | ISR | Auditory | *F* (1, 18) = 9.00, *MSE* = .003,  *p* = .008, *η^2^_p_* = .333 | *F* (1, 18) = 1.98, *MSE* = .005  *p* = .177, *η^2^_p_* = .099 | *F* (1, 18) = 1.98, *MSE* = .005  *p* = .177, *η^2^_p_* = .099 |
|  | ISR | Visual | *-* | *-* | *-* |
| 4 | ISR | Auditory | *F* (1, 18) = 0.88, *MSE* = .069,  *p* = .361, *η^2^_p_* = .047 | *F* (3, 54) = 2.56, *MSE* = .015  *p* = .065, *η^2^_p_* = .124 | *F* (3, 54) = 0.39, *MSE* = .015  *p* = .758, *η^2^_p_* = .021 |
|  | ISR | Visual | *F* (1, 18) = 0.45, *MSE* = .083,  *p* = .447, *η^2^_p_* = .032 | ***F* (3, 54) = 8.05, *MSE* = .027**  ***p* < .001, *η^2^_p_* = .309** | *F* (3, 54) = 0.53, *MSE* = .027  *p* = .666, *η^2^_p_* = .028 |
| 5 | ISR | Auditory | *F* (1, 18) = 0.49, *MSE* = .210,  *p* = .494, *η^2^_p_* = .026 | ***F* (4, 72) = 18.75, *MSE* = .033**  ***p* < .001, *η^2^_p_* = .510** | *F* (4, 72) = 0.71, *MSE* = .033  *p* = .587, *η^2^_p_* = .038 |
|  | ISR | Visual | *F* (1, 18) = 0.05, *MSE* = .269,  *p* = .820, *η^2^_p_* = .003 | ***F* (4, 72) = 21.95, *MSE* = .034**  ***p* < .001, *η^2^_p_* = .549** | *F* (4, 72) = 0.60, *MSE* = .034  *p* = .596, *η^2^_p_* = .037 |
| 6 | ISR | Auditory | *F* (1, 18) = 0.01, *MSE* = .207,  *p* = .968, *η^2^_p_* < .001 | ***F* (5, 90) = 18.38, *MSE* = .043**  ***p* < .001, *η^2^_p_* = .505** | *F* (5, 90) = 0.80, *MSE* = .043  *p* = .556, *η^2^_p_* = .042 |
|  | ISR | Visual | *F* (1, 18) = 0.81, *MSE* = .119,  *p* = .379, *η^2^_p_* = .043 | ***F* (5, 90) = 31.16, *MSE* = .039**  ***p* < .001, *η^2^_p_* = .634** | *F* (5, 90) = 0.29, *MSE* = .039  *p* = .916, *η^2^_p_* = .016 |
| 7 | ISR | Auditory | *F* (1, 18) = 0.02, *MSE* = .210,  *p* = .884, *η^2^_p_* = .001 | ***F* (6, 108) = 17.40, *MSE* = .057**  ***p* < .001, *η^2^_p_* = .492** | *F* (6, 108) = 2.19, *MSE* = .057  *p* = .050, *η^2^_p_* = .108 |
|  | ISR | Visual | *F* (1, 18) = 0.95, *MSE* = .077,  *p* = .343, *η^2^_p_* = .050 | ***F* (6, 108) = 19.71, *MSE* = .047**  ***p* < .001, *η^2^_p_* = .523** | *F* (6, 108) = 0.52, *MSE* = .047  *p* = .794, *η^2^_p_* = .028 |
| 8 | ISR | Auditory | *F* (1, 18) = 0.33, *MSE* = .129,  *p* = .574, *η^2^_p_* = .018 | ***F* (7, 126) = 18.74, *MSE* = .056**  ***p* < .001, *η^2^_p_* = .510** | ***F* (7, 126) = 3.26, *MSE* = .056**  ***p* = .003, *η^2^_p_* = .154** |
|  | ISR | Visual | *F* (1, 18) < 0.001, *MSE* = .134,  *p* = .983, *η^2^_p_* < .001 | ***F* (7, 126) = 10.86, *MSE* = .053**  ***p* < .001, *η^2^_p_* = .376** | *F* (7, 126) = 1.08, *MSE* = .053  *p* = .378, *η^2^_p_* = .057 |
| 12 | ISR | Auditory | *F* (1, 18) = 0.02, *MSE* = .087,  *p* = .897, *η^2^_p_* = .001 | ***F* (11, 198) = 22.87, *MSE* = .046**  ***p* < .001, *η^2^_p_* = .560** | *F* (11, 198) = 1.08, *MSE* = .046  *p* = .378, *η^2^_p_* = .057 |
|  | ISR | Visual | *F* (1, 18) = 0.21, *MSE* = .064,  *p* = .652, *η^2^_p_* = .012 | ***F* (11, 198) = 10.13, *MSE* = .039**  ***p* < .001, *η^2^_p_* = .360** | *F* (11, 198) = 0.58, *MSE* = .039  *p* = .843, *η^2^_p_* = .031 |

*Note:* Significant main effects and interactions are presented in bold. Bonferroni corrected *p*-level = .0071. ISR visual LL2 could not be computed due to no variation within the values.

**Table A3.** Experiment 1. Analyses of the PFR data shown in Figure A5. The data were subjected to a 2 (block: 1 or 2) x 7 (list length: 2, 4, 5, 6, 7, 8, and 12) mixed ANOVA.

| **Task** | **Modality** | **Main Effects** | | **2-way interaction** |
| --- | --- | --- | --- | --- |
|  |  | **Block** | **LL** | **Block x LL** |
| Probability of first recall = Serial Position 1 | | | | |
| IFR | Auditory | ***F* (1, 18) = 9.86, *MSE* = .111,**  ***p* = .006, *η^2^_p_* = .354** | ***F* (6, 108) = 36.37, *MSE* = .038**  ***p* < .001, *η^2^_p_* = .669** | *F* (6, 108) = 1.88, *MSE* = .038  *p* = .091, *η^2^_p_* = .095 |
| IFR | Visual | *F* (1, 18) = 1.57, *MSE* = .223,  *p* = .227, *η^2^_p_* = .080 | ***F* (6, 108) = 39.07, *MSE* = .045**  ***p* < .001, *η^2^_p_* = .685** | *F* (6, 108) = 0.82, *MSE* = .045  *p* = .556, *η^2^_p_* = .044 |
| ISR | Auditory | *F* (1, 18) = 2.91, *MSE* = .199,  *p* = .105, *η^2^_p_* = .139 | ***F* (6, 108) = 21.56, *MSE* = .030**  ***p* < .001, *η^2^_p_* = .545** | *F* (6, 108) = 1.07, *MSE* = .030  *p* = .386, *η^2^_p_* = .056 |
| ISR | Visual | *F* (1, 18) = 0.03, *MSE* = .101,  *p* = .875, *η^2^_p_* = .001 | ***F* (6, 108) = 16.85, *MSE* = .035**  ***p* < .001, *η^2^_p_* = .483** | *F* (6, 108) = 0.36, *MSE* = .035  *p* = .904, *η^2^_p_* = .020 |
|  |  |  |  |  |
| Probability of first recall = Last four | | | | |
| IFR | Auditory | ***F* (1, 18) = 5.75, *MSE* = .161,**  ***p* = .028, *η^2^_p_* = .242** | ***F* (6, 108) = 16.91, *MSE* = .041**  ***p* < .001, *η^2^_p_* = .484** | *F* (6, 108) = 1.87, *MSE* = .041  *p* = .092, *η^2^_p_* = .094 |
| IFR | Visual | *F* (1, 18) = 2.57, *MSE* = .178,  *p* = .126, *η^2^_p_* = .125 | ***F* (6, 108) = 22.32, *MSE* = .049**  ***p* < .001, *η^2^_p_* = .554** | ***F* (6, 108) = 2.28, *MSE* = .049**  ***p* = .041, *η^2^_p_* = .112** |
| ISR | Auditory | *F* (1, 12) = 2.53, *MSE* = .157,  *p* = .138, *η^2^_p_* = .174 | ***F* (6, 72) = 9.53, *MSE* = .020**  ***p* < .001, *η^2^_p_* = .443** | ***F* (6, 72) = 2.88, *MSE* = .020**  ***p* = .014, *η^2^_p_* = .193** |
| ISR | Visual | *F* (1, 14) = 1.95, *MSE* = .041,  *p* = .184, *η^2^_p_* = .122 | ***F* (6, 84) = 5.02, *MSE* = .020**  ***p* < .001, *η^2^_p_* = .264** | *F* (6, 84) = 1.62, *MSE* = .020  *p* = .151, *η^2^_p_* = .104 |

*Note:* Significant main effects and interactions are presented in bold.

**Table A4.** Experiment 2. Analyses of the SPCs, shown in Figure A6 and A7, using FR scoring for IFR. At each LL, the data were subjected to a 2 (block: 1 or 2) x *n* (SP: 1, …, *n*, where *n* is the LL) mixed ANOVA. The values represent the p-value.

| **List length** | **Task** | **Modality** | **Main Effects** | | **2-way interaction** |
| --- | --- | --- | --- | --- | --- |
|  |  |  | **Block** | **SP** | **Block x SP** |
| 2 | IFR | Read aloud | *F* (1, 18) = 0.72, *MSE* = .006,  *p* = .407, *η^2^_p_* = .038 | *F* (1, 18) = 2.88, *MSE* = .006  *p* = .107, *η^2^_p_* = .138 | *F* (1, 18) = 0.72, *MSE* = .006  *p* = .407, *η^2^_p_* = .038 |
|  | IFR | Visual | *F* (1, 18) = 2.25, *MSE* = .002,  *p* = .151, *η^2^_p_* = .111 | *F* (1, 18) < 0.001, *MSE* = .002  *p* = 1.000, *η^2^_p_* < .001 | *F* (1, 18) < 0.001, *MSE* = .002  *p* = 1.000, *η^2^_p_* < .001 |
| 4 | IFR | Read aloud | *F* (1, 18) < 0.001, *MSE* = .020,  *p* = 1.000, *η^2^_p_* < .001 | *F* (3, 54) = 0.86, *MSE* = .011  *p* = .466, *η^2^_p_* = .046 | *F* (3, 54) = 0.86, *MSE* = .011  *p* = .466, *η^2^_p_* = .046 |
|  | IFR | Visual | *F* (1, 18) = 1.29, *MSE* = .031,  *p* = .271, *η^2^_p_* = .067 | *F* (3, 54) = 3.72, *MSE* = .021  *p* = .017, *η^2^_p_* = .171 | *F* (3, 54) = 0.48, *MSE* = .021  *p* = .699, *η^2^_p_* = .026 |
| 5 | IFR | Read aloud | *F* (1, 18) = 2.61, *MSE* = .035,  *p* = .124, *η^2^_p_* = .126 | ***F* (4, 72) = 4.05, *MSE* = .041**  ***p* = .005, *η^2^_p_* = .184** | *F* (4, 72) = 1.61, *MSE* = .041  *p* = .182, *η^2^_p_* = .082 |
|  | IFR | Visual | *F* (1, 18) = 0.50, *MSE* = .086,  *p* = .504, *η^2^_p_* = .025 | ***F* (4, 72) = 9.23, *MSE* = .037**  ***p* < .001, *η^2^_p_* = .339** | *F* (4, 72) = 2.76, *MSE* = .037  *p* = .034, *η^2^_p_* = .133 |
| 6 | IFR | Read aloud | *F* (1, 18) = 2.28, *MSE* = .091,  *p* = .148, *η^2^_p_* = .113 | ***F* (5, 90) = 5.71, *MSE* = .060**  ***p* < .001, *η^2^_p_* = .241** | *F* (5, 90) = 1.33, *MSE* = .060  *p* = .260, *η^2^_p_* = .069 |
|  | IFR | Visual | *F* (1, 18) = 0.16, *MSE* = .102,  *p* = .694, *η^2^_p_* = .009 | ***F* (5, 90) = 3.84, *MSE* = .050**  ***p* = .003, *η^2^_p_* = .176** | *F* (5, 90) = 0.15, *MSE* = .050  *p* = .980, *η^2^_p_* = .008 |
| 7 | IFR | Read aloud | *F* (1, 18) = 0.57, *MSE* = .098,  *p* = .459, *η^2^_p_* = .031 | ***F* (6, 108) = 9.84, *MSE* = .056**  ***p* < .001, *η^2^_p_* = .353** | *F* (6, 108) = 1.76, *MSE* = .056  *p* = .114, *η^2^_p_* = .089 |
|  | IFR | Visual | *F* (1, 18) = 1.75, *MSE* = .065,  *p* = .203, *η^2^_p_* = .089 | ***F* (6, 108) = 4.30, *MSE* = .062**  ***p* < .001, *η^2^_p_* = .193** | *F* (6, 108) = 1.14, *MSE* = .062  *p* = .346, *η^2^_p_* = .059 |
| 8 | IFR | Read aloud | *F* (1, 18) = 0.64, *MSE* = .053,  *p* = .638, *η^2^_p_* = .013 | ***F* (7, 126) = 22.32, *MSE* = .045**  ***p* < .001, *η^2^_p_* = .554** | ***F* (7, 126) = 3.77, *MSE* = .045**  ***p* = .001, *η^2^_p_* = .173** |
|  | IFR | Visual | *F* (1, 18) = 0.49, *MSE* = .087,  *p* = .494, *η^2^_p_* = .026 | ***F* (7, 126) = 8.79, *MSE* = .062**  ***p* < .001, *η^2^_p_* = .328** | *F* (7, 126) = 1.54, *MSE* = .062  *p* = .160, *η^2^_p_* = .079 |
| 12 | IFR | Read aloud | *F* (1, 18) = 0.39, *MSE* = .070,  *p* = .391, *η^2^_p_* = .041 | ***F* (11, 198) = 40.96, *MSE* = .036**  ***p* < .001, *η^2^_p_* = .695** | *F* (11, 198) = 1.65, *MSE* = .036  *p* = .087, *η^2^_p_* = .084 |
|  | IFR | Visual | *F* (1, 18) = 0.07, *MSE* = .092,  *p* = .801, *η^2^_p_* = .004 | ***F* (11, 198) = 22.42, *MSE* = .042**  ***p* < .001, *η^2^_p_* = .555** | *F* (11, 198) = 1.38, *MSE* = .042  *p* = .184, *η^2^_p_* = .071 |

*Note:* Significant main effects and interactions are presented in bold. Bonferroni corrected *p*-level = .0071.

**Table A5**. Experiment 2. Analyses of the SPCs, shown in Figure A8 and A9, using SR scoring for ISR. At each LL, the data were subjected to a 2 (block: 1 or 2) x *n* (SP: 1, …, *n*, where *n* is the LL) mixed ANOVA.

| **List length** | **Task** | **Modality** | **Main Effects** | | **2-way interaction** |
| --- | --- | --- | --- | --- | --- |
|  |  |  | **Block** | **SP** | **Block x SP** |
| 2 | IFR | Read aloud | *F* (1, 18) = 1.00, *MSE* = .001,  *p* = .331, *η^2^_p_* = .053 | *F* (1, 18) = 1.00, *MSE* = .001  *p* = .331, *η^2^_p_* = .053 | *F* (1, 18) = 1.00, *MSE* = .001  *p* = .331, *η^2^_p_* = .053 |
|  | IFR | Visual | *F* (1, 18) = 1.00, *MSE* = .009,  *p* = .331, *η^2^_p_* = .053 | *F* (1, 18) = 1.00, *MSE* = .001  *p* = .331, *η^2^_p_* = .053 | *F* (1, 18) = 1.00, *MSE* = .001  *p* = .331, *η^2^_p_* = .053 |
| 4 | IFR | Read aloud | *F* (1, 18) = 0.42, *MSE* = .043,  *p* = .525, *η^2^_p_* = .023 | *F* (3, 54) = 1.67, *MSE* = .008  *p* = .183, *η^2^_p_* = .085 | *F* (3, 54) = 2.93, *MSE* = .008  *p* = .042, *η^2^_p_* = .140 |
|  | IFR | Visual | *F* (1, 18) = 0.53, *MSE* = .044,  *p* = .531, *η^2^_p_* = .022 | ***F* (3, 54) = 11.91, *MSE* = .014**  ***p* < .001, *η^2^_p_* = .398** | *F* (3, 54) = 1.26, *MSE* = .014  *p* = .299, *η^2^_p_* = .065 |
| 5 | IFR | Read aloud | *F* (1, 18) = 0.30, *MSE* = .164,  *p* = .594, *η^2^_p_* = .016 | ***F* (4, 72) = 14.33, *MSE* = .036**  ***p* < .001, *η^2^_p_* = .443** | *F* (4, 72) = 1.92, *MSE* = .036  *p* = .116, *η^2^_p_* = .097 |
|  | IFR | Visual | *F* (1, 18) = 0.53, *MSE* = .163,  *p* = .528, *η^2^_p_* = .022 | ***F* (4, 72) = 12.13, *MSE* = .046**  ***p* < .001, *η^2^_p_* = .402** | *F* (4, 72) = 0.87, *MSE* = .046  *p* = .488, *η^2^_p_* = .046 |
| 6 | IFR | Read aloud | *F* (1, 18) = 0.26, *MSE* = .250,  *p* = .616, *η^2^_p_* = .014 | ***F* (5, 90) = 8.00, *MSE* = .057**  ***p* < .001, *η^2^_p_* = .308** | *F* (5, 90) = 1.28, *MSE* = .057  *p* = .278, *η^2^_p_* = .067 |
|  | IFR | Visual | *F* (1, 18) = 1.56, *MSE* = .206,  *p* = .228, *η^2^_p_* = .080 | ***F* (5, 90) = 8.14, *MSE* = .057**  ***p* < .001, *η^2^_p_* = .311** | *F* (5, 90) = 0.44, *MSE* = .057  *p* = .821, *η^2^_p_* = .024 |
| 7 | IFR | Read aloud | *F* (1, 18) = 2.50, *MSE* = .132,  *p* = .131, *η^2^_p_* = .122 | ***F* (6, 108) = 13.53, *MSE* = .055**  ***p* < .001, *η^2^_p_* = .429** | *F* (6, 108) = 0.70, *MSE* = .055  *p* = .647, *η^2^_p_* = .038 |
|  | IFR | Visual | *F* (1, 18) = 0.80, *MSE* = .206,  *p* = .383, *η^2^_p_* = .043 | ***F* (6, 108) = 13.16, *MSE* = .050**  ***p* < .001, *η^2^_p_* = .422** | *F* (6, 108) = 2.77, *MSE* = .050  *p* = .015, *η^2^_p_* = .133 |
| 8 | IFR | Read aloud | *F* (1, 18) = 3.71, *MSE* = .124,  *p* = .070, *η^2^_p_* = .171 | ***F* (7, 126) = 16.34, *MSE* = .051**  ***p* < .001, *η^2^_p_* = .476** | *F* (7, 126) = 1.81, *MSE* = .051  *p* = .091, *η^2^_p_* = .091 |
|  | IFR | Visual | *F* (1, 18) = 0.01, *MSE* = .193,  *p* = .943, *η^2^_p_* < .001 | ***F* (7, 126) = 16.51, *MSE* = .049**  ***p* < .001, *η^2^_p_* = .478** | *F* (7, 126) = 0.57, *MSE* = .049  *p* = .783, *η^2^_p_* = .030 |
| 12 | IFR | Read aloud | *F* (1, 18) = 0.59, *MSE* = .056,  *p* = .454, *η^2^_p_* = .032 | ***F* (11, 198) = 34.31, *MSE* = .039**  ***p* < .001, *η^2^_p_* = .656** | ***F* (11, 198) = 2.90, *MSE* = .039**  ***p* = .001, *η^2^_p_* = .139** |
|  | IFR | Visual | *F* (1, 18) = 0.78, *MSE* = .070,  *p* = .390, *η^2^_p_* = .041 | ***F* (11, 198) = 14.93, *MSE* = .041**  ***p* < .001, *η^2^_p_* = .453** | *F* (11, 198) = 0.57, *MSE* = .041  *p* = .854, *η^2^_p_* = .031 |

*Note:* Significant main effects and interactions are presented in bold. Bonferroni corrected *p*-level = .0071.

**Table A6.** Experiment 2. Analyses of the PFR data shown in Figure A10. The data were subjected to a 2 (block: 1 or 2) x 7 (list length: 2, 4, 5, 6, 7, 8, and 12) mixed ANOVA.

| **Task** | **Modality** | **Main Effects** | | **2-way interaction** |
| --- | --- | --- | --- | --- |
|  |  | **Block** | **LL** | **Block x LL** |
| Probability of first recall = Serial Position 1 | | | | |
| IFR | Read aloud | *F* (1, 18) = 2.97, *MSE* = .222,  *p* = .102, *η^2^_p_* = .142 | ***F* (6, 108) = 46.78, *MSE* = .044**  ***p* < .001, *η^2^_p_* = .722** | *F* (6, 108) = 1.42, *MSE* = .044  *p* = .213, *η^2^_p_* = .073 |
| IFR | Visual | *F* (1, 18) = 0.47, *MSE* = .175,  *p* = .500, *η^2^_p_* = .026 | ***F* (6, 108) = 22.96, *MSE* = .040**  ***p* < .001, *η^2^_p_* = .561** | *F* (6, 108) = 1.68, *MSE* = .040  *p* = .134, *η^2^_p_* = .085 |
| ISR | Read aloud | *F* (1, 18) = 0.01, *MSE* = .160,  *p* = .934, *η^2^_p_* < .001 | ***F* (6, 108) = 39.82, *MSE* = .050**  ***p* < .001, *η^2^_p_* = .689** | *F* (6, 108) = 0.46, *MSE* = .050  *p* = .836, *η^2^_p_* = .025 |
| ISR | Visual | *F* (1, 18) = 0.13, *MSE* = .111,  *p* = .727, *η^2^_p_* = .007 | ***F* (6, 108) = 11.90, *MSE* = .033**  ***p* < .001, *η^2^_p_* = .398** | *F* (6, 108) = 0.33, *MSE* = .033  *p* = .922, *η^2^_p_* = .018 |
|  |  |  |  |  |
| Probability of first recall = Last four | | | | |
| IFR | Read aloud | *F* (1, 18) = 1.45, *MSE* = .255,  *p* = .244, *η^2^_p_* = .075 | ***F* (6, 108) = 33.53, *MSE* = .043**  ***p* < .001, *η^2^_p_* = .651** | *F* (6, 108) = 1.62, *MSE* = .043  *p* = .149, *η^2^_p_* = .083 |
| IFR | Visual | *F* (1, 14) = 0.47, *MSE* = .093,  *p* = .506, *η^2^_p_* = .032 | ***F* (6, 84) = 6.20, *MSE* = .029**  ***p* < .001, *η^2^_p_* = .307** | *F* (6, 84) = 0.72, *MSE* = .029  *p* = .633, *η^2^_p_* = .049 |
| ISR | Read aloud | *F* (1, 18) = 0.66, *MSE* = .191,  *p* = .428, *η^2^_p_* = .035 | ***F* (6, 108) = 27.27, *MSE* = .051**  ***p* < .001, *η^2^_p_* = .602** | *F* (6, 108) = 1.13, *MSE* = .051  *p* = .349, *η^2^_p_* = .059 |
| ISR | Visual | *F* (1, 13) = 0.15, *MSE* = .042,  *p* = .701, *η^2^_p_* = .012 | ***F* (6, 78) = 2.34, *MSE* = .026**  ***p* = .040, *η^2^_p_* = .152** | *F* (6, 78) = 0.90, *MSE* = .026  *p* = .500, *η^2^_p_* = .065 |

*Note:* Significant main effects and interactions are presented in bold.


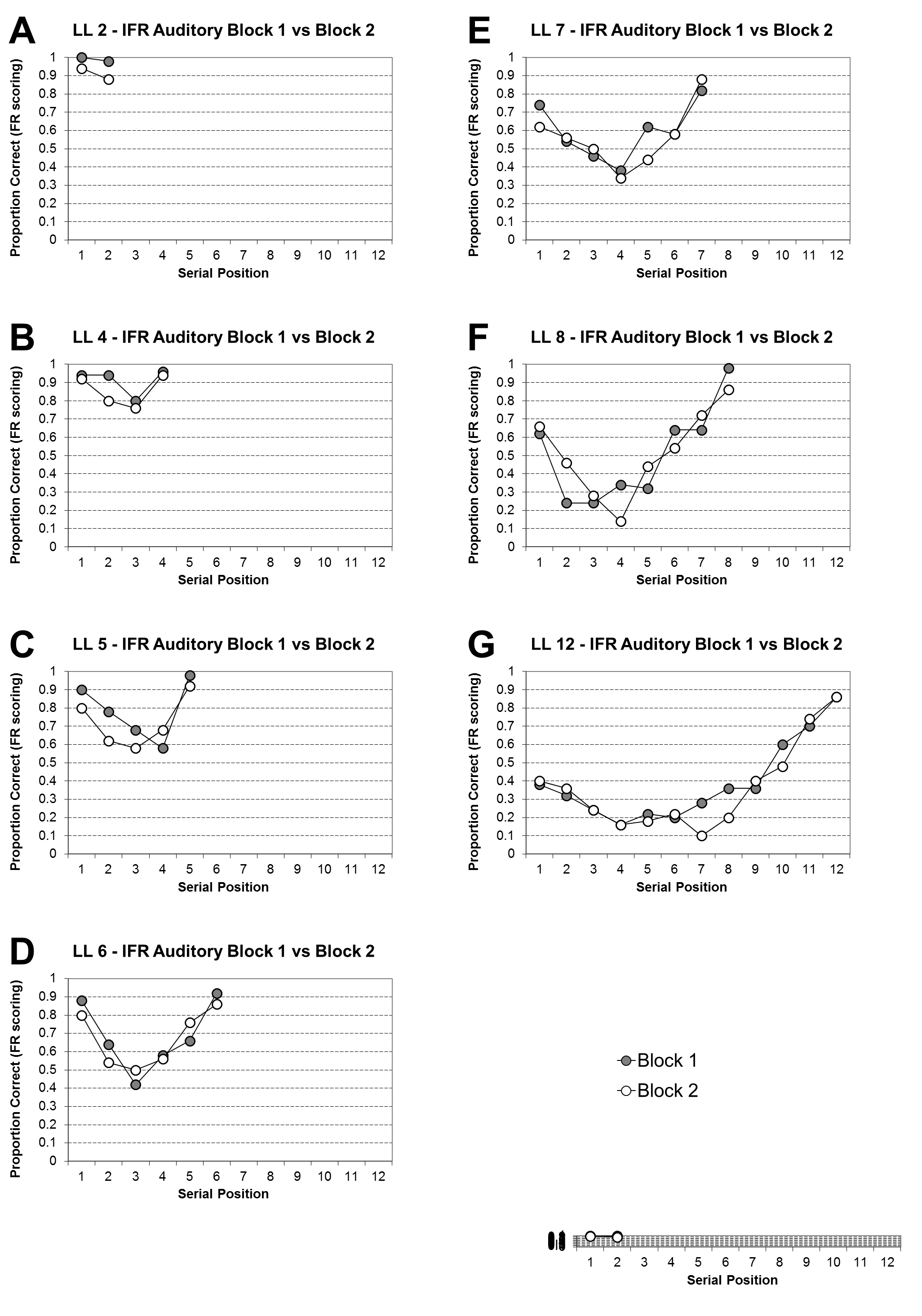


**Figure A1.** Experiment 1. Comparison of IFR auditory block 1 and block 2 using FR scoring.


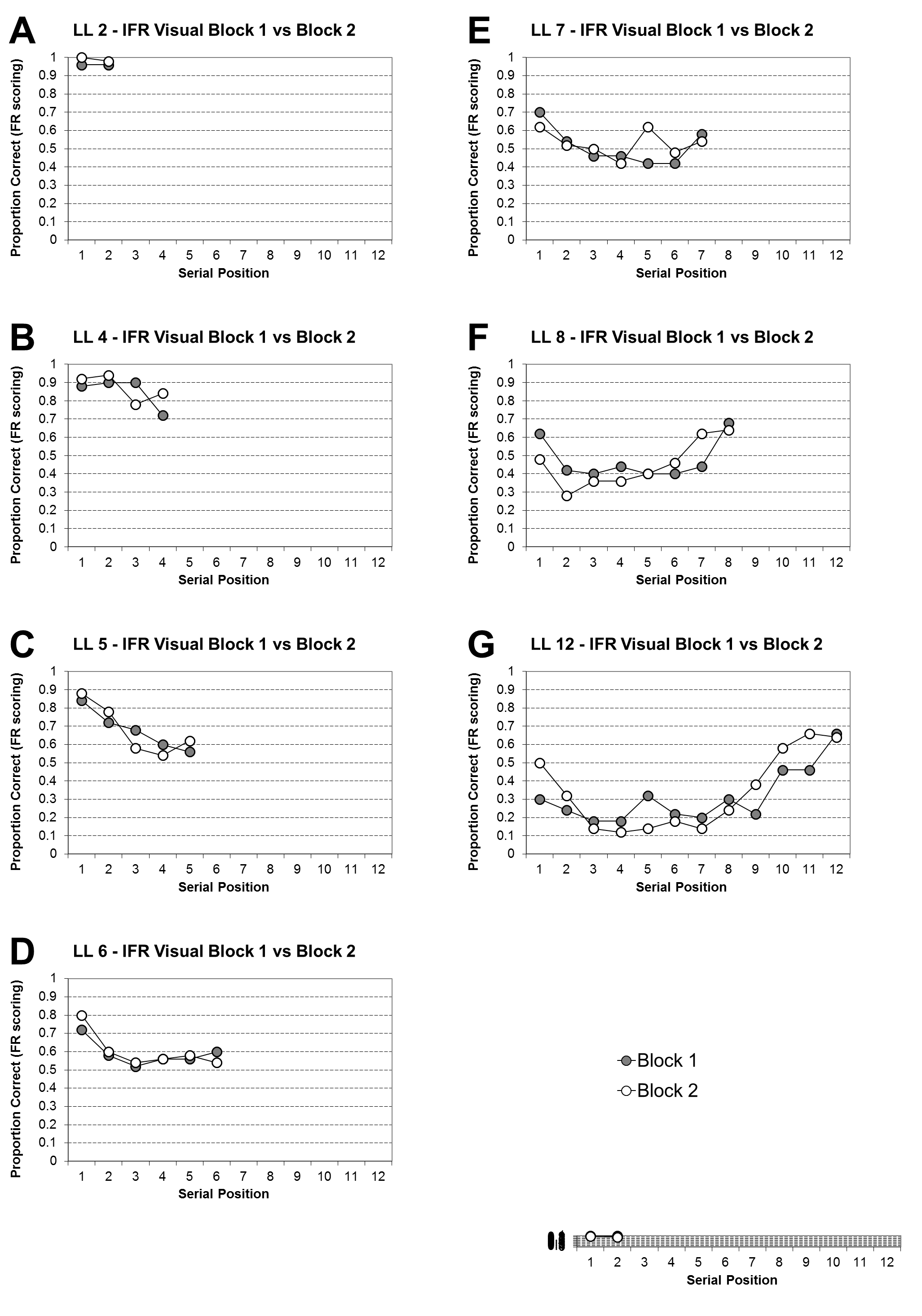


**Figure A2**. Experiment 1. Comparison of IFR visual block 1 and block 2 using FR scoring.


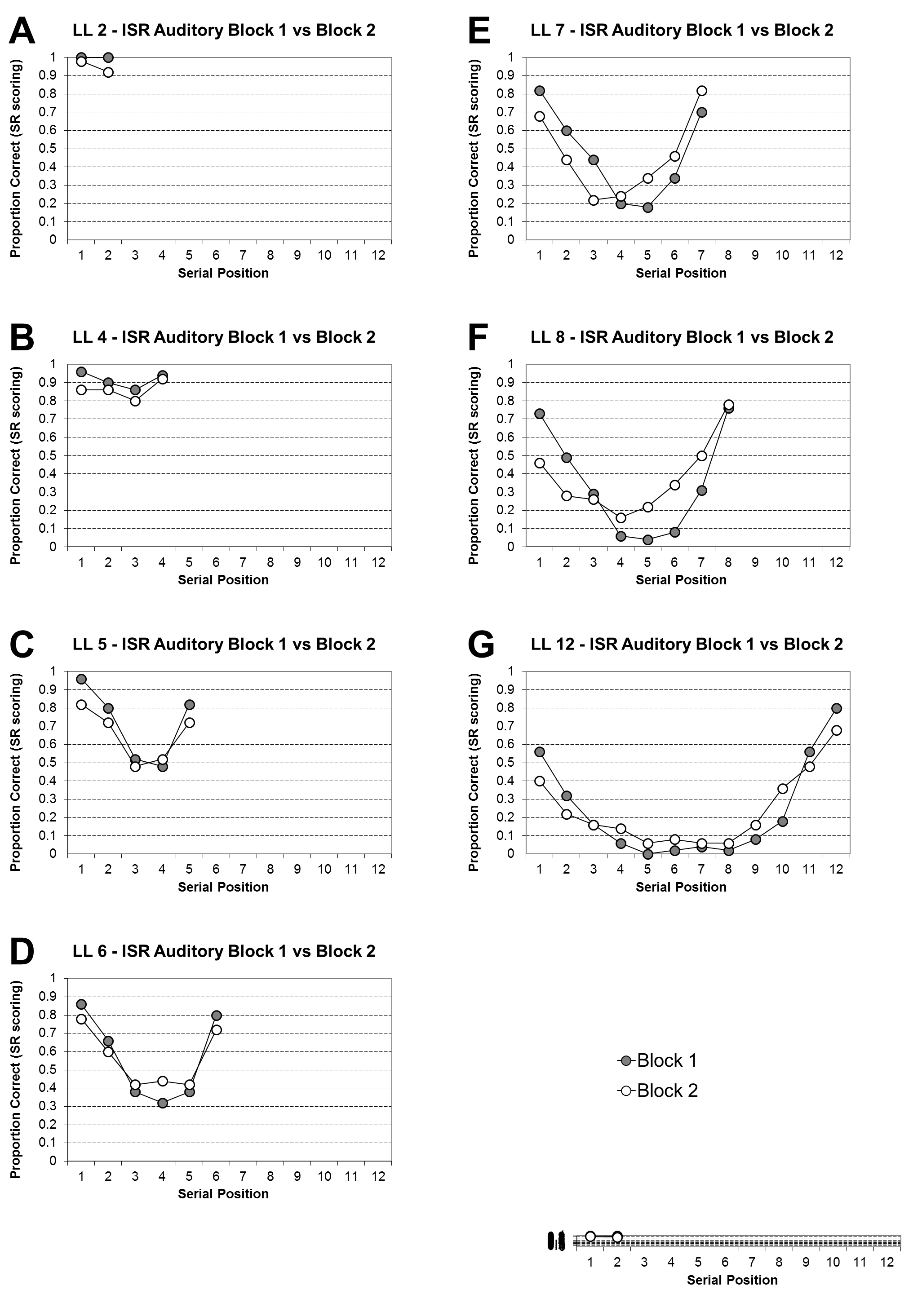


**Figure A3**. Experiment 1. Comparison of ISR auditory block 1 and block 2 using SR scoring.


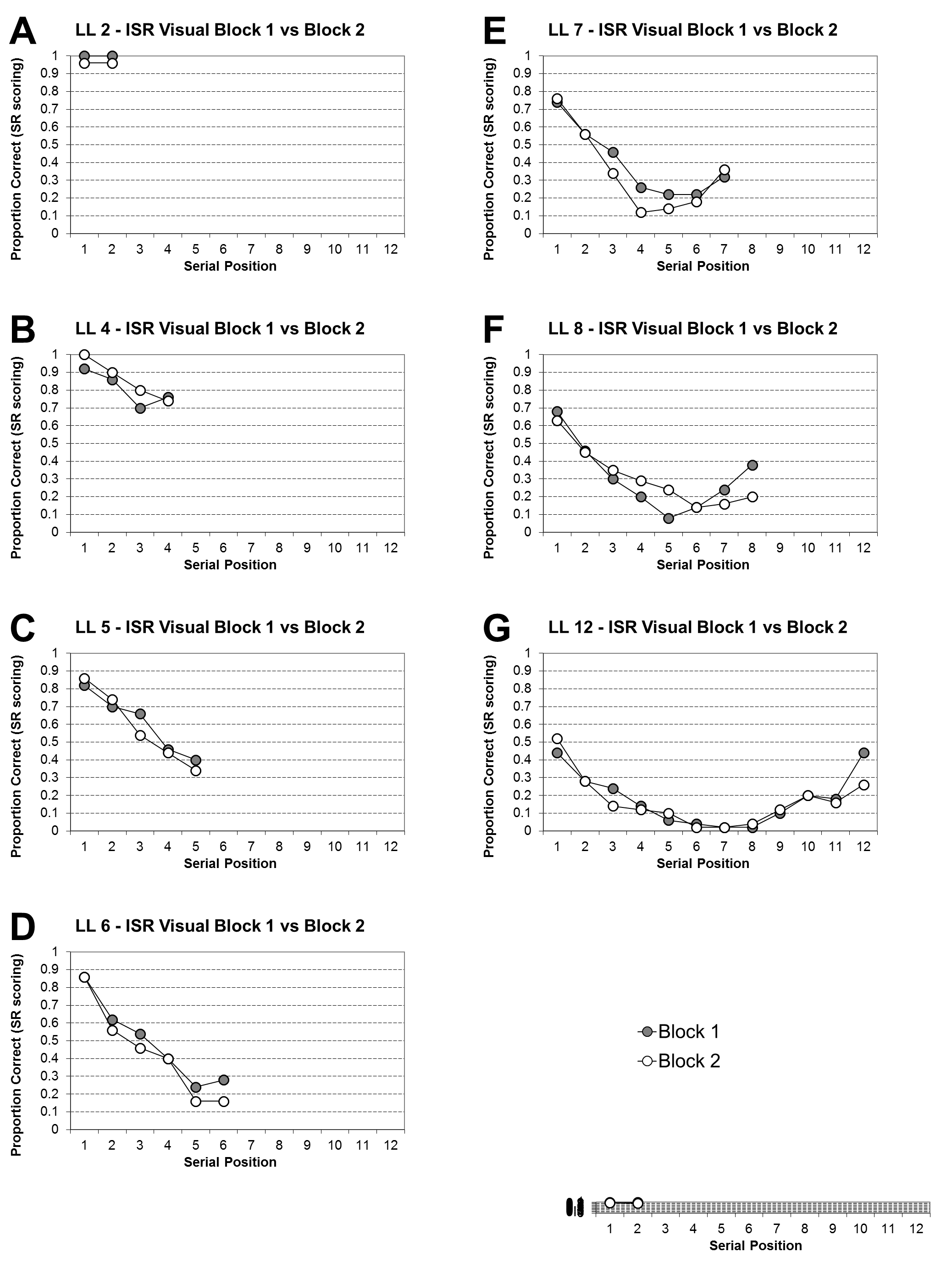


**Figure A4**. Experiment 1. Comparison of ISR visual block 1 and block 2 using SR scoring.


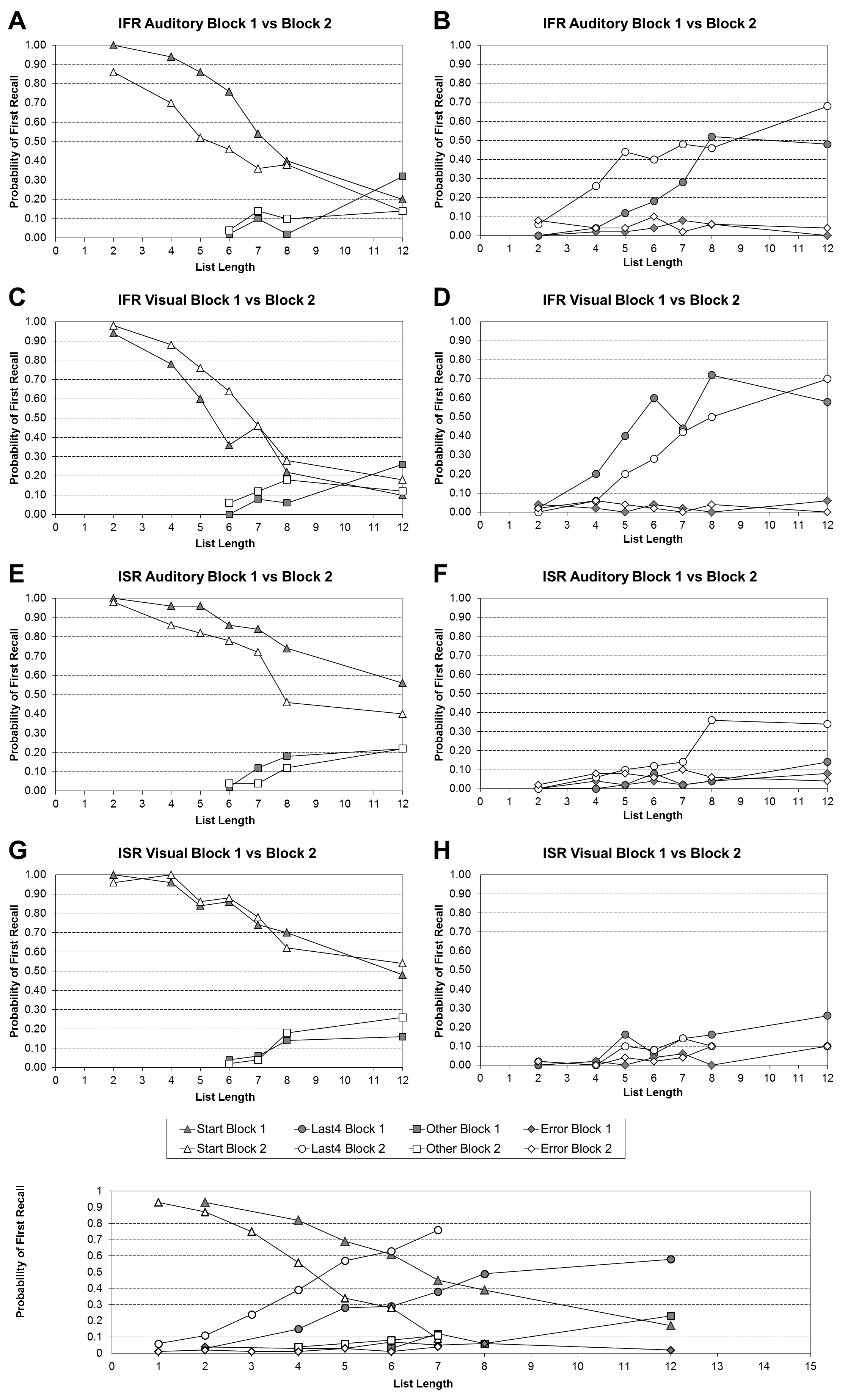


**Figure A5**. Experiment 1. Comparison of PFR for block 1 and block 2 for both tasks.


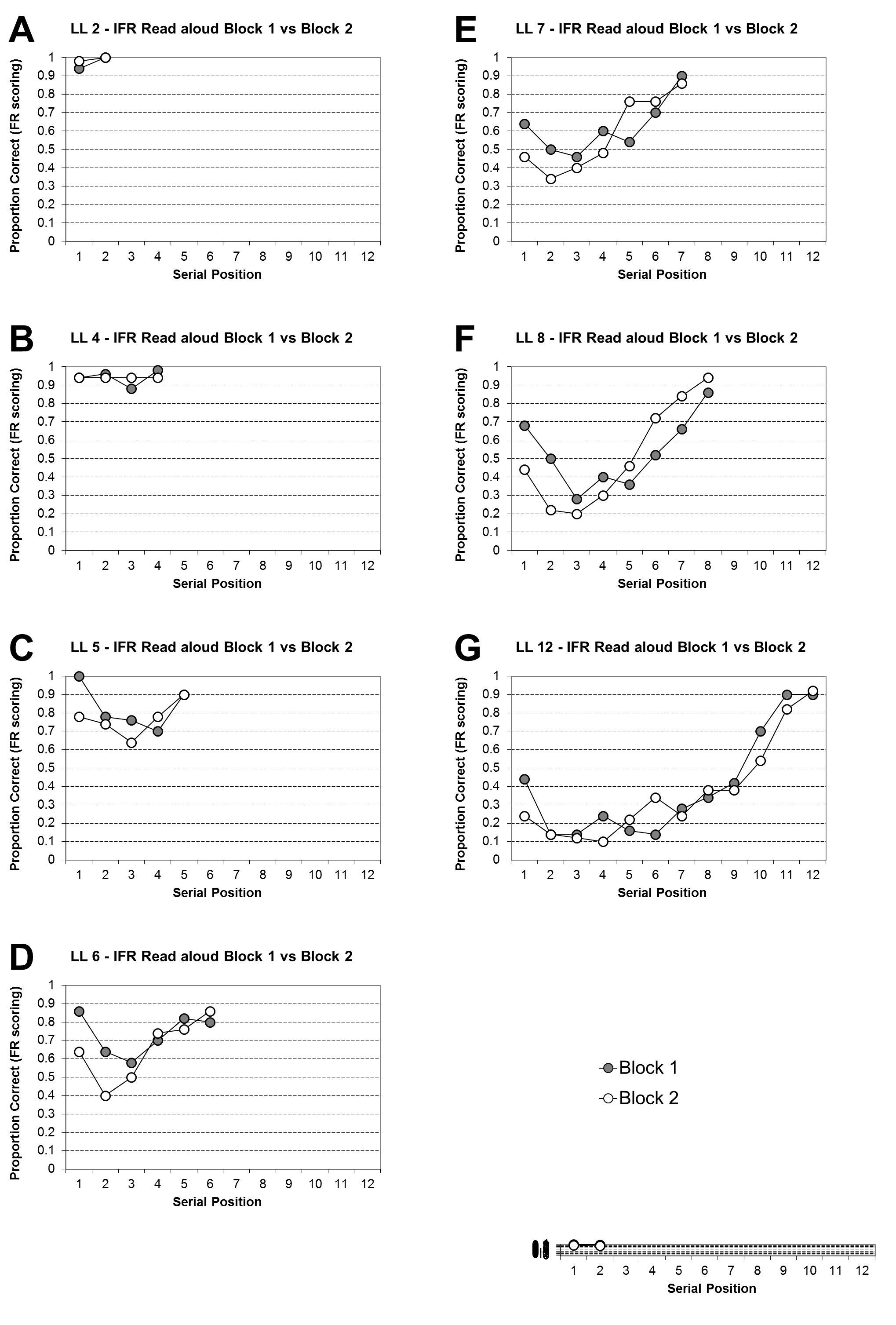


**Figure A6**. Experiment 2. Comparison of IFR read aloud block 1 and block 2 using FR scoring.


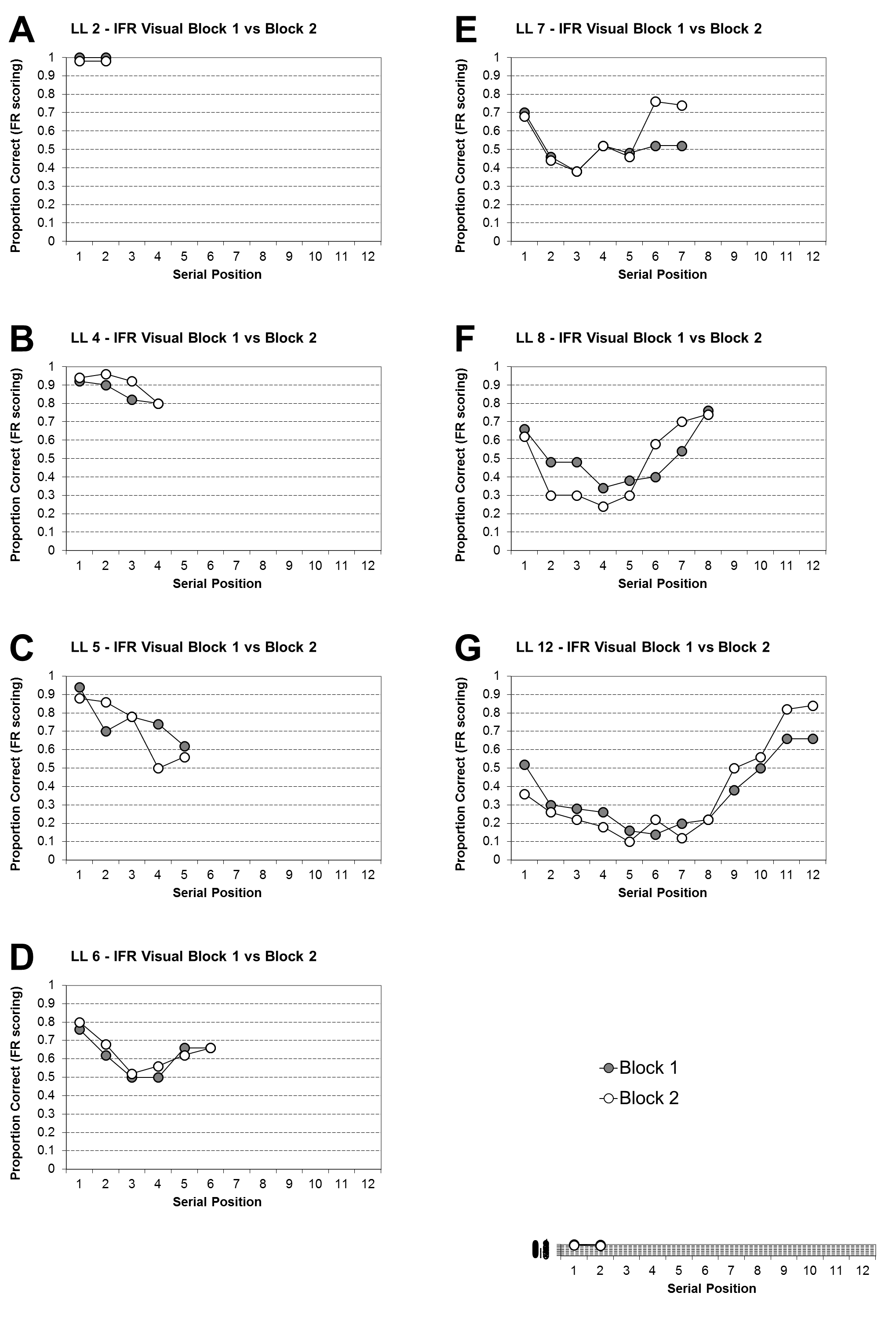


**Figure A7.** Experiment 2. Comparison of IFR visual block 1 and block 2 using FR scoring.


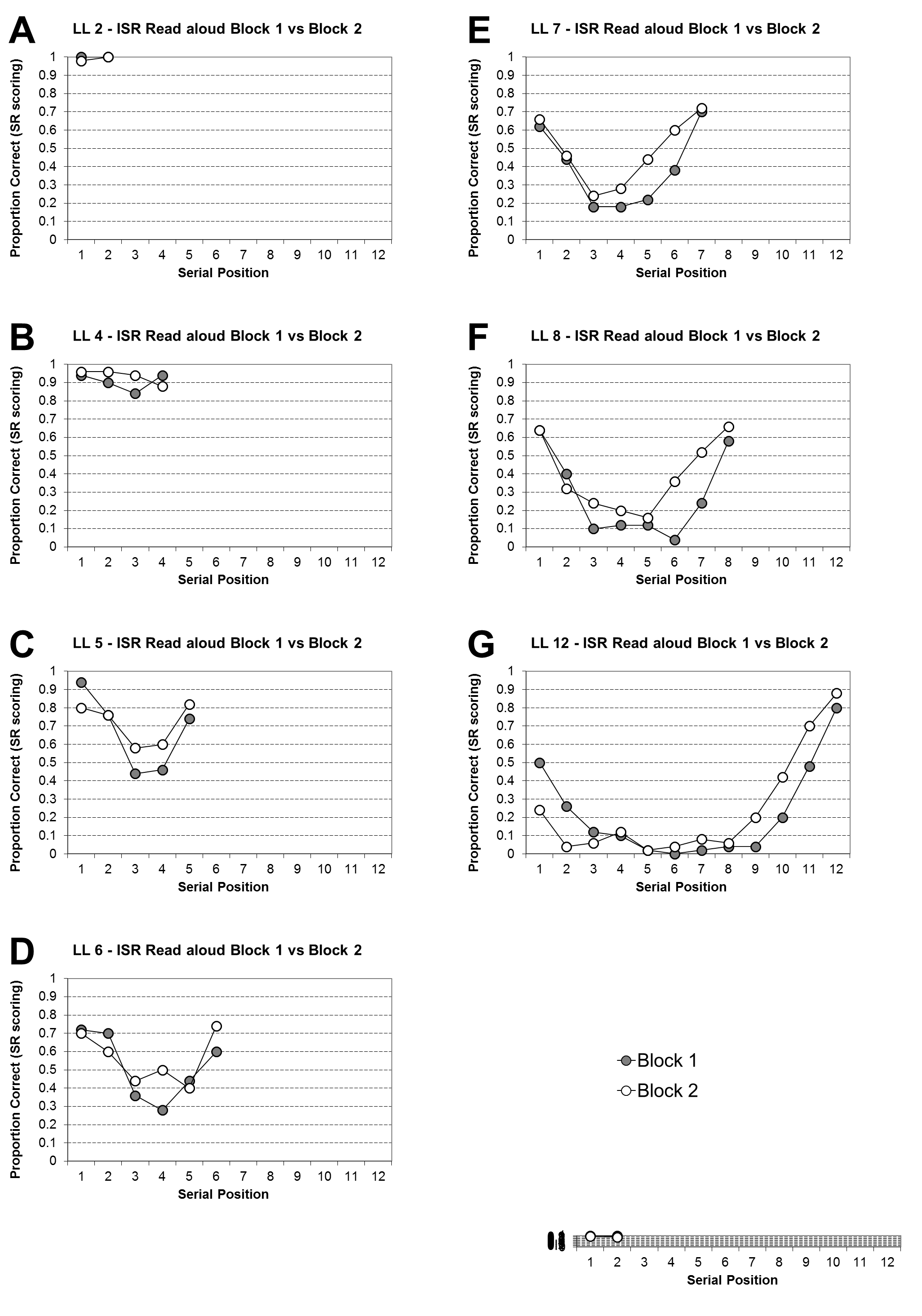


**Figure A8**. Experiment 2. Comparison of ISR read aloud block 1 and block 2 using SR scoring.


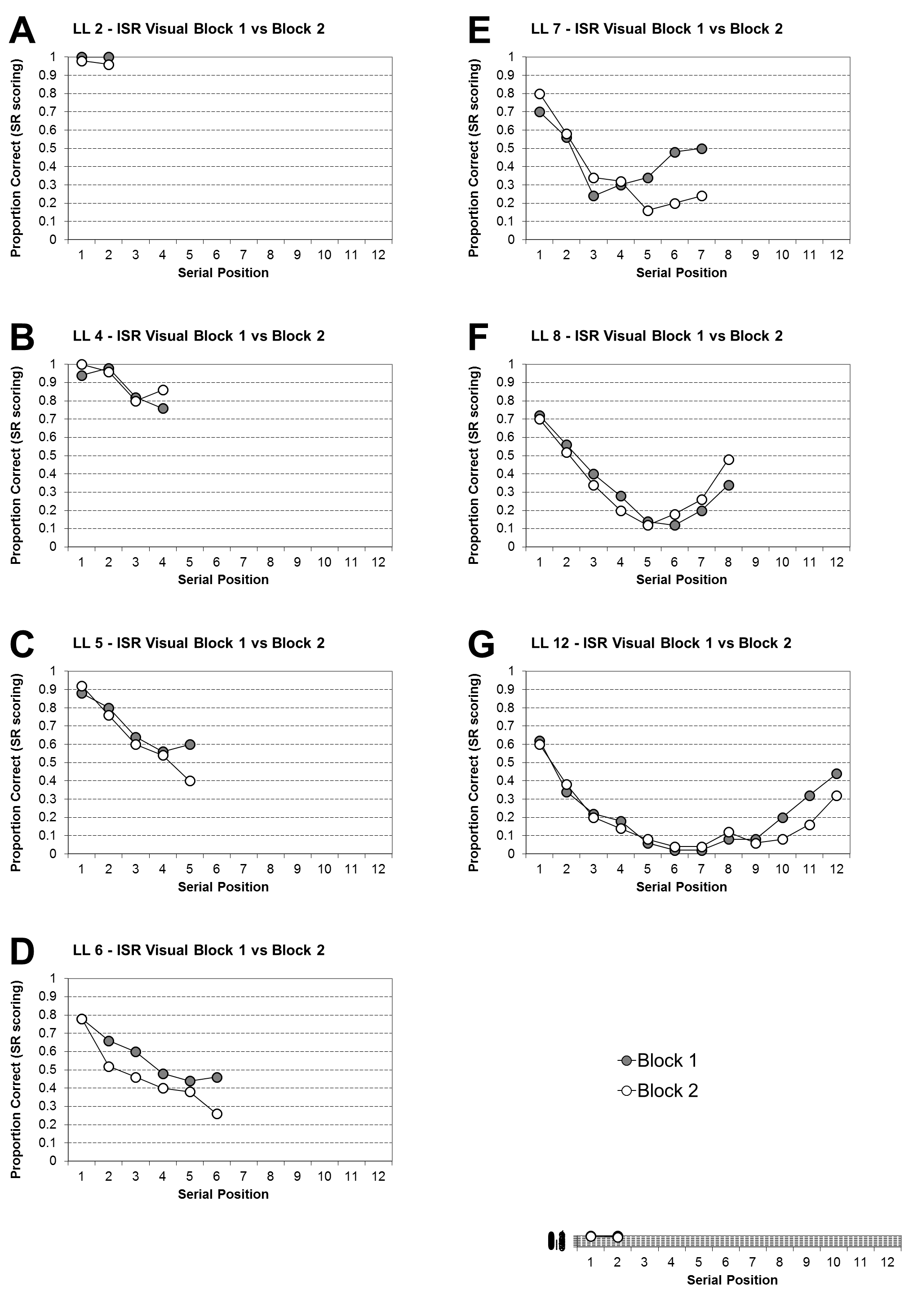


**Figure A9**. Experiment 2. Comparison of ISR visual block 1 and block 2 using SR scoring.

**
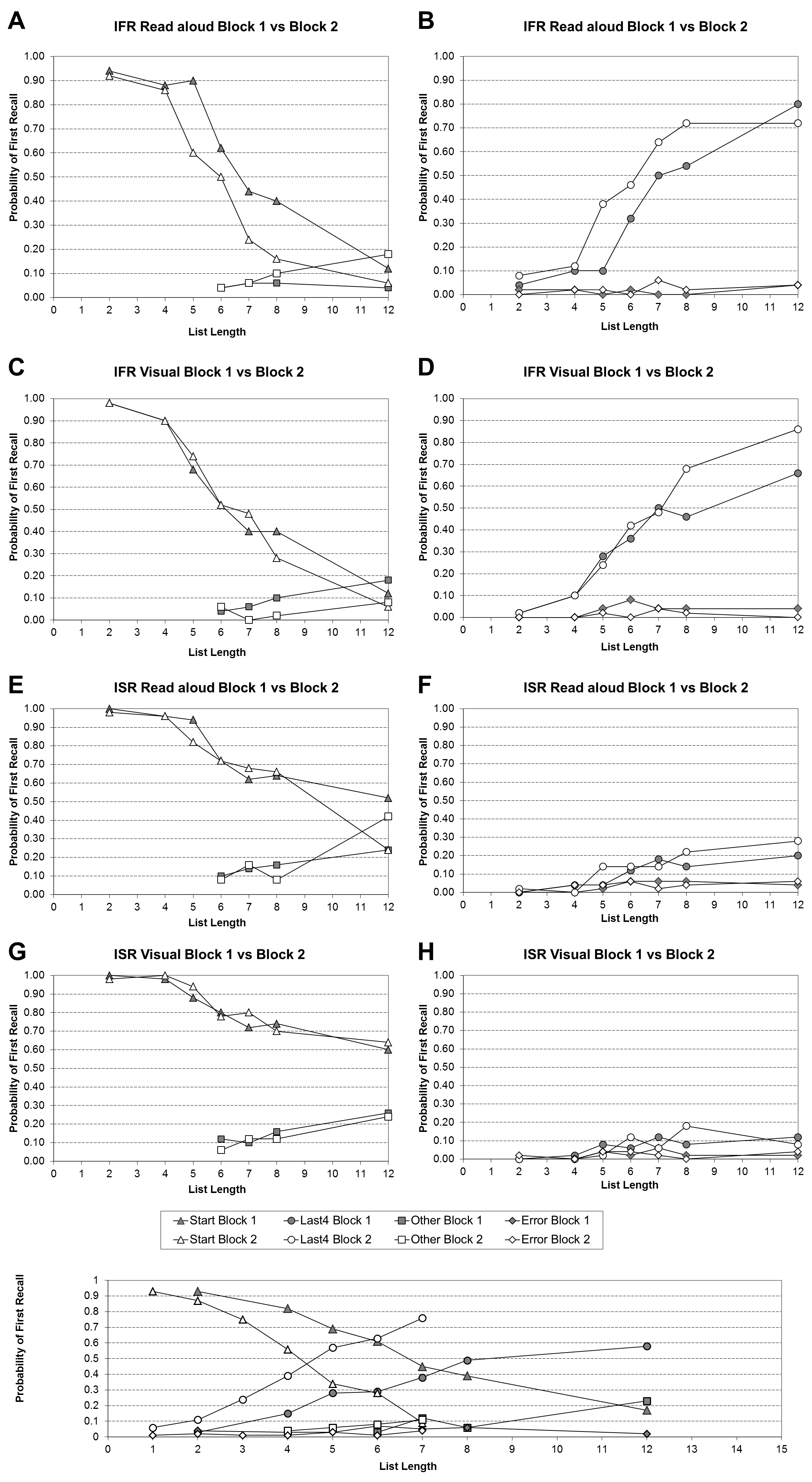
Figure A10**. Experiment 2. Comparison of PFR for block 1 and block 2 for both tasks.

**Supplementary Appendices B1-B24: Detailed analyses of serial position curves following the Analyses of Variance (ANOVAs)**

**Experiment 1.** Supplementary Appendices for Experiment 1 detailing the effect of modality, serial position, and their interaction for each list length for IFR and ISR with different scoring systems (Appendix B1-B4), analyses of resultant serial position curves starting at the start of the list (Appendix B5-B8), and analyses of resultant serial position curves starting at the end of the list (Appendix B9-B12).

**Experiment 2.** Supplementary Appendices for Experiment 2 detailing the effect of modality, serial position, and their interaction for each list length for IFR and ISR with different scoring systems (Appendix B13-B16), analyses of resultant serial position curves starting at the start of the list (Appendix B17-B20), and analyses of resultant serial position curves starting at the end of the list (Appendix B21-B24).

**Appendix B1**. Experiment 1. Analyses of the SPCs for the auditory and visual IFR and ISR conditions using FR scoring. At each LL, the data were subjected to a 2 (task: IFR or ISR) x 2 (modality: auditory or visual) x *n* (SP: 1, …, *n*, where *n* is the LL) mixed ANOVA.

| **LL** | **Main Effects** | | | **2-way interactions** | | | **3-way interaction** |
| --- | --- | --- | --- | --- | --- | --- | --- |
|  | **Task** | **Modality** | **SP** | **Task x Modality** | **Task x SP** | **Modality x SP** | **Task x Modality x SP** |
| 2 | *F* (1, 38) = 0.564,  *MSE* = .022,  *p* = .457, *η^2^_p_* = .015 | *F* (1, 38) = 0.979,  *MSE* = .013,  *p* = .329, *η^2^_p_* = .025 | *F* (1, 38) = 4.03,  *MSE* = .003,  *p*  = .052, *η^2^_p_* = .096 | *F* (1, 38) = 0.180,  *MSE* = .013,  *p* = .674, *η^2^_p_* = .005 | *F* (1, 38) = 0.740,  *MSE* = .003,  *p*  = .395, *η^2^_p_* = .019 | *F* (1, 38) = 3.96,  *MSE* = .003,  *p*  = .054, *η^2^_p_* = .094 | *F* (1, 38) = 0.081,  *MSE* = .003,  *p* = .778, *η^2^_p_* = .002 |
| 4 | *F* (1, 38) = 0.362,  *MSE* = .100,  *p* = .551, *η^2^_p_* = .009 | *F* (1, 38) = 3.05,  *MSE* = .030,  *p* = .089, *η^2^_p_* = .074 | ***F* (3, 114) = 7.41,**  ***MSE* = .023,**  ***p*  < .001, *η^2^_p_* = .163** | *F* (1, 38) = 0.339,  *MSE* = .030,  *p* = .564, *η^2^_p_* = .009 | *F* (3, 114) = 0.164,  *MSE* = .023,  *p*  = .921, *η^2^_p_* = .004 | ***F* (3, 114) = 6.21,**  ***MSE* = .025,**  ***p*  = .001, *η^2^_p_* = .140** | *F* (3, 114) = 1.86,  *MSE* = .025,  *p* = .141, *η^2^_p_* = .047 |
| 5 | *F* (1, 38) = 0.893,  *MSE* = .145,  *p* = .351, *η^2^_p_* = .023, | ***F* (1, 38) = 11.1,**  ***MSE* = .058,**  ***p* = .002, *η^2^_p_* = .226** | ***F* (4, 152) = 16.0,**  ***MSE* = .042,**  ***p*  < .001, *η^2^_p_* = .296** | *F* (1, 38) = 0.111,  *MSE* = .058,  *p* = .741, *η^2^_p_* = .003 | *F* (4, 152) = 2.44,  *MSE* = .042,  *p*  = .049, *η^2^_p_* = .060 | ***F* (4, 152) = 14.5,**  ***MSE* = .035,**  ***p*  < .001, *η^2^_p_* = .276** | *F* (4, 152) = 0.575,  *MSE* = .035,  *p* = .681, *η^2^_p_* = .015 |
| 6 | *F* (1, 38) = 0.044,  *MSE* = .154,  *p* = .835, *η^2^_p_* = .001 | ***F* (1, 38) = 21.0,**  ***MSE* = .040,**  ***p*  < .001, *η^2^_p_* = .356** | ***F* (5, 190) = 16.1,**  ***MSE* = .053,**  ***p*  < .001, *η^2^_p_* = .297** | *F* (1, 38) = 0.051,  *MSE* = .040,  *p* = .822, *η^2^_p_* = .001 | ***F* (5, 190) = 6.15,**  ***MSE* = .053,**  ***p*  < .001, *η^2^_p_* = .139** | ***F* (5, 190) = 16.1,**  ***MSE* = .040,**  ***p*  < .001, *η^2^_p_* = .297** | *F* (5, 190) = 1.71,  *MSE* = .040,  *p* = .135, *η^2^_p_* = .043 |
| 7 | *F* (1, 38) = 0.049,  *MSE* = .147,  *p* = .826, *η^2^_p_* = .001 | ***F* (1, 38) = 16.3,**  ***MSE* = .044,**  ***p* < .001, *η^2^_p_* = .300** | ***F* (6, 228) = 13.6,**  ***MSE* = .066,**  ***p* < .001, *η^2^_p_* = .264** | *F* (1, 38) = 0.787,  *MSE* = .044,  *p* = .380, *η^2^_p_* = .020 | *F* (6, 228) = 1.52,  *MSE* = .066,  *p =* .172, *η^2^_p_* = .038 | ***F* (6, 228) = 7.26,**  ***MSE* = .057,**  ***p* < .001, *η^2^_p_* = .160** | *F* (6, 228) = 0.535,  *MSE* = .057,  *p* = .781, *η^2^_p_* = .014 |
| 8 | *F* (1, 38) = 0.094,  *MSE* = .139,  *p* = .760, *η^2^_p_* = .002 | *F* (1, 38) = 7.41,  *MSE* = .037,  *p* = .010, *η^2^_p_* = .163 | ***F* (7, 266) = 22.7,**  ***MSE* = .062,**  ***p* < .001, *η^2^_p_* = .374** | *F* (1, 38) = 0.051,  *MSE* = .037,  *p* = .823, *η^2^_p_* = .001 | ***F* (7, 266) = 5.04,**  ***MSE* = .062,**  ***p* < .001, *η^2^_p_* = .117** | ***F* (7, 266) = 10.9,**  ***MSE* = .053,**  ***p* < .001, *η^2^_p_* = .222** | *F* (7, 266) = 1.11,  *MSE* = .053,  *p* = .358, *η^2^_p_* = .028 |
| 12 | *F* (1, 38) = 1.53,  *MSE* = .192  *p* = .224, *η^2^_p_* = .039 | ***F* (1, 38) = 18.9,**  ***MSE* = .031,**  ***p*  < .001, *η^2^_p_* = .332** | ***F* (11, 418) = 41.7,**  ***MSE* = .057,**  ***p*  < .001, *η^2^_p_* = .523** | *F* (1, 38) = 0.049,  *MSE* = .031,  *p* = .826, *η^2^_p_* = .001 | ***F* (11, 418) = 2.44,**  ***MSE* = .057,**  ***p*  = .006, *η^2^_p_* = .060** | ***F* (11, 418) = 7.14,**  ***MSE* = .039,**  ***p*  < .001, *η^2^_p_* = .158** | *F* (11, 418) = 2.14,  *MSE* = .039,  *p* = .017, *η^2^_p_* = .053 |

*Note:* Significant main effects and interactions are presented in bold. Bonferroni corrected *p*-level = .0071.

**Appendix B2**. Experiment 1. Analyses of the IFR SPCs, shown in Figure 2, using FR scoring. At each LL, the data were subjected to a 2 (modality: auditory or visual) x *n* (SP: 1, …, *n*, where *n* is the LL) within-subjects ANOVA.

| **LL** | **Main Effects** | | **2-way interaction** | **SME (Modality advantage: SP)** |
| --- | --- | --- | --- | --- |
|  | **Modality** | **SP** | **Modality x SP** |  |
| 2 | *F* (1, 19) = 0.629, *MSE* = .020,  *p* = .437, *η^2^_p_* = .032 | *F* (1, 19) = 4.13, *MSE* = .003,  *p*  = .056, *η^2^_p_* = .179 | *F* (1, 19) = 1.31, *MSE* = .003,  *p*  = .267, *η^2^_p_* = .064 | - |
| 4 | *F* (1, 19) = 0.588, *MSE* = .034,  *p* = .453, *η^2^_p_* = .030 | *F* (3, 57) = 3.63, *MSE* = .023,  *p*  = .018, *η^2^_p_* = .160 | *F* (3, 57) = 3.73, *MSE* = .030,  *p*  = .016, *η^2^_p_* = .164 | 4 |
| 5 | *F* (1, 19) = 5.47, *MSE* = .047,  *p* = .030, *η^2^_p_* = .223 | ***F* (4, 76) = 8.37, *MSE* = .052,**  ***p*  < .001, *η^2^_p_* = .306** | ***F* (4, 76) = 6.48, *MSE* = .042,**  ***p*  < .001, *η^2^_p_* = .254** | 5 |
| 6 | ***F* (1, 19) = 10.4, *MSE* = .037,**  ***p* = .004, *η^2^_p_* = .353** | ***F* (5, 95) = 8.36, *MSE* = .060,**  ***p*  < .001, *η^2^_p_* = .306** | ***F* (5, 95) = 4.03, *MSE* = .047,**  ***p*  = .002, *η^2^_p_* = .175** | 5, 6 |
| 7 | *F* (1, 19) = 5.04, *MSE* = .043,  *p* = .037, *η^2^_p_* = .210 | ***F* (6, 114) = 6.00, *MSE* = .075,**  ***p* < .001, *η^2^_p_* = .240** | *F* (6, 114) = 2.54, *MSE* = .057,  *p* = .024, *η^2^_p_* = .118 | 7 |
| 8 | *F* (1, 19) = 3.98, *MSE* = .041,  *p* = .061, *η^2^_p_* = .173 | ***F* (7, 133) = 24.0, *MSE* = .048,**  ***p* < .001, *η^2^_p_* = .558** | ***F* (7, 133) = 3.85, *MSE* = .055,**  ***p* = .001, *η^2^_p_* = .168** | 4, 6, 7, 8 |
| 12 | ***F* (1, 19) = 13.4, *MSE* = .024,**  ***p* = .002, *η^2^_p_* = .414** | ***F* (11, 209) = 28.0, *MSE* = .054,**  ***p*  < .001, *η^2^_p_* = .595** | *F* (11, 209) = 1.05, *MSE* = .049,  *p*  = .406, *η^2^_p_* = .052 | 11, 12 |

*Note:* Significant main effects and interactions are presented in bold; underlined values signify a significant inversion of the modality effect. Bonferroni corrected *p*-level = .0071.

**Appendix B3**. Experiment 1. Analyses of the ISR SPCs using FR scoring. At each LL, the data were subjected to a 2 (modality: auditory or visual) x *n* (SP: 1, …, *n*, where *n* is the LL) within-subjects ANOVA.

| **LL** | **Main Effects** | **2-way interaction** |  | **SME (Modality advantage: SP)** |
| --- | --- | --- | --- | --- |
|  | **Modality** | **SP** | **Modality x SP** |  |
| 2 | *F* (1, 19) = 0.388, *MSE* = .005,  *p* = .541, *η^2^_p_* = .020 | *F* (1, 19) = 0.655, *MSE* = .003,  *p*  = .428, *η^2^_p_* = .033 | *F* (1, 19) = 2.92 *MSE* = .003,  *p*  = .104, *η^2^_p_* = .133 | - |
| 4 | *F* (1, 19) = 3.21, *MSE* = .025,  *p* = .089, *η^2^_p_* = .145 | *F* (3, 57) = 3.94, *MSE* = .023,  *p*  = .013, *η^2^_p_* = .172 | ***F* (3, 57) = 4.49, *MSE* = .020,**  ***p*  = .007, *η^2^_p_* = .191** | 4 |
| 5 | *F* (1, 19) = 5.69, *MSE* = .068,  *p* = .028, *η^2^_p_* = .230 | ***F* (4, 76) = 10.6, *MSE* = .032,**  ***p*  < .001, *η^2^_p_* = .357** | ***F* (4, 76) = 9.16, *MSE* = .028,**  ***p*  < .001, *η^2^_p_* = .325** | 5 |
| 6 | ***F* (1, 19) = 10.7, *MSE* = .044,**  ***p* = .004, *η^2^_p_* = .359** | ***F* (5, 95) = 14.6, *MSE* = .047,**  ***p*  < .001, *η^2^_p_* = .435** | ***F* (5, 95) = 15.9, *MSE* = .033,**  ***p*  < .001, *η^2^_p_* = .456** | 5, 6 |
| 7 | ***F* (1, 19) = 11.9, *MSE* = .045,**  ***p* = .003, *η^2^_p_* = .385** | ***F* (6, 114) = 9.65, *MSE* = .057,**  ***p* < .001, *η^2^_p_* = .337** | ***F* (6, 114) = 5.24, *MSE* = .057,**  ***p* < .001, *η^2^_p_* = .216** | 6, 7 |
| 8 | *F* (1, 19) = 3.43, *MSE* = .034,  *p* = .080, *η^2^_p_* = .153 | ***F* (7, 133) = 7.43, *MSE* = .075,**  ***p* < .001, *η^2^_p_* = .281** | ***F* (7, 133) = 8.26, *MSE* = .052,**  ***p* < .001, *η^2^_p_* = .303** | 7, 8 |
| 12 | ***F* (1, 19) = 6.97, *MSE* = .037,**  ***p*  = .016, *η^2^_p_* = .268** | ***F* (11, 209) = 16.7, *MSE* = .060,**  ***p*  < .001, *η^2^_p_* = .468** | ***F* (11, 209) = 10.5, *MSE* = .030,**  ***p*  < .001, *η^2^_p_* = .356** | 2, 4, 5, 10, 11, 12 |

*Note:* Significant main effects and interactions are presented in bold; underlined values signify a significant inversion of the modality effect. Bonferroni corrected *p*-level = .0071.

**Appendix B4**. Experiment 1. Analyses of the ISR SPCs, shown in Figure 3, using SR scoring. At each LL, the data were subjected to a 2 (modality: auditory or visual) x *n* (SP: 1, …, *n*, where *n* is the LL) within-subjects ANOVA.

| **LL** | **Main Effects** | | **2-way interaction** | **SME (Modality advantage: SP)** |
| --- | --- | --- | --- | --- |
|  | **Modality** | **SP** | **Modality x SP** |  |
| 2 | *F* (1, 19) = 0.073, *MSE* = .007,  *p* = .789, *η^2^_p_* = .004 | *F* (1, 19) = 1.88, *MSE* = .002,  *p*  = .186, *η^2^_p_* = .090 | *F* (1, 19) = 1.88, *MSE* = .002,  *p*  = .186, *η^2^_p_* = .090 | - |
| 4 | *F* (1, 19) = 2.85, *MSE* = .039,  *p* = .108, *η^2^_p_* = .130 | ***F* (3, 57) = 7.53, *MSE* = .020,**  ***p*  < .001, *η^2^_p_* = .284** | ***F* (3, 57) = 5.00, *MSE* = .020,**  ***p*  = .004, *η^2^_p_* = .208** | 4 |
| 5 | *F* (1, 19) = 7.89, *MSE* = .049,  *p* = .011, *η^2^_p_* = .293 | ***F* (4, 76) = 23.7, *MSE* = .042,**  ***p*  < .001, *η^2^_p_* = .555** | ***F* (4, 76) = 15.0, *MSE* = .023,**  ***p*  < .001, *η^2^_p_* = .442** | 5 |
| 6 | ***F* (1, 19) = 9.72, *MSE* = .066,**  ***p* = .006, *η^2^_p_* = .338** | ***F* (5, 95) = 29.7, *MSE* = .049,**  ***p*  < .001, *η^2^_p_* = .610** | ***F* (5, 95) = 18.0, *MSE* = .031,**  ***p*  < .001, *η^2^_p_* = .486** | 5, 6 |
| 7 | *F* (1, 19) = 7.58, *MSE* = .072,  *p* = .013, *η^2^_p_* = .285 | ***F* (6, 114) = 30.3, *MSE* = .054,**  ***p* < .001, *η^2^_p_* = .615** | ***F* (6, 114) = 5.55, *MSE* = .053,**  ***p* < .001, *η^2^_p_* = .226** | 6, 7 |
| 8 | *F* (1, 19) = 2.34, *MSE* = .079,  *p* = .143, *η^2^_p_* = .109 | ***F* (7, 133) = 18.0, *MSE* = .068,**  ***p* < .001, *η^2^_p_* = .487** | ***F* (7, 133) = 8.35, *MSE* = .048,**  ***p* = .001, *η^2^_p_* = .305** | 7, 8 |
| 12 | ***F* (1, 19) = 18.7, *MSE* = .026,**  ***p*  < .001, *η^2^_p_* = .496** | ***F* (11, 209) = 20.5, *MSE* = .061,**  ***p*  < .001, *η^2^_p_* = .519** | ***F* (11, 209) = 8.91, *MSE* = .024,**  ***p*  < .001, *η^2^_p_* = .319** | 11, 12 |

*Note:* Significant main effects and interactions are presented in bold. Bonferroni corrected *p*-level = .0071.

**Appendix B5.** Experiment 1. Analyses of the SPCs using only data from trials starting with SP1 for the auditory and visual IFR and ISR conditions using FR scoring. At each LL, the data were subjected to a 2 (task: IFR or ISR) x 2 (modality: auditory or visual) *n-1* (SP: 1, …, *n*, where *n* is the LL) mixed ANOVA.

| **LL** | **Main Effects** | | | **2-way interactions** | | | **3-way interaction** |
| --- | --- | --- | --- | --- | --- | --- | --- |
|  | **Task** | **Modality** | **SP** | **Task x Modality** | **Task x SP** | **Modality x SP** | **Task x Modality x SP** |
| 4 | *F* (1, 37) = 0.221,  *MSE* = .075,  *p* = .641, *η^2^_p_*= .006 | *F* (1, 37) = 5.47,  *MSE* = .031,  *p* = .025, *η^2^_p_*= .129 | ***F* (2, 74) = 6.33,**  ***MSE* = .024,**  ***p*  = .003, *η^2^_p_*= .146** | *F* (1, 37) = 2.98,  *MSE* = .031,  *p* = .093, *η^2^_p_*= .074 | *F* (2, 74) = 0.538,  *MSE* = .024,  *p*  = .586, *η^2^_p_*= .014 | *F* (2, 74) = 4.96,  *MSE* = .029,  *p*  = .010, *η^2^_p_*= .118 | *F* (2, 74) = 0.252,  *MSE* = .029,  *p* = .778, *η^2^_p_*= .007 |
| 5 | *F* (1, 38) = 2.69,  *MSE* = .156,  *p* = .109, *η^2^_p_*= .066 | ***F* (1, 38) = 12.6,**  ***MSE* = .076,**  ***p* = .001, *η^2^_p_*= .249** | ***F* (3, 114) = 9.56,**  ***MSE* = .070,**  ***p*  < .001, *η^2^_p_*= .201** | *F* (1, 38) = 0.016,  *MSE* = .076,  *p* = .900, *η^2^_p_*< .001 | *F* (3, 114) = 1.27,  *MSE* = .070,  *p*  = .287, *η^2^_p_*= .032 | ***F* (3, 114) = 19.7,**  ***MSE* = .057,**  ***p*  < .001, *η^2^_p_*= .341** | *F* (3, 114) = 1.65,  *MSE* = .057,  *p* = .183, *η^2^_p_*= .041 |
| 6 | *F* (1, 36) = 0.167,  *MSE* = .188,  *p* = .685, *η^2^_p_*= .005 | ***F* (1, 36) = 16.7,**  ***MSE* = .049,**  ***p*  < .001, *η^2^_p_*= .316** | ***F* (4, 144) = 16.1,**  ***MSE* = .078,**  ***p*  < .001, *η^2^_p_*= .195** | *F* (1, 36) = 0.690,  *MSE* = .049,  *p* = .412, *η^2^_p_*= .019 | *F* (4, 144) = 3.35,  *MSE* = .078,  *p*  = .012, *η^2^_p_*= .085 | ***F* (4, 144) = 17.9,**  ***MSE* = .074,**  ***p*  < .001, *η^2^_p_*= .332** | *F* (4, 144) = 0.514,  *MSE* = .074,  *p* = .514, *η^2^_p_*= .022 |
| 7 | *F* (1, 32) = 0.685,  *MSE* = .157,  *p* = .414, *η^2^_p_*= .021 | ***F* (1, 32) = 12.1,**  ***MSE* = .070,**  ***p* = .001, *η^2^_p_*= .275** | ***F* (5, 160) = 6.65,**  ***MSE* = .106,**  ***p* < .001, *η^2^_p_*= .172** | *F* (1, 32) < 0.001,  *MSE* = .070,  *p* = .986, *η^2^_p_*< .001 | *F* (5, 160) = 0.234,  *MSE* = .106,  *p =* .947, *η^2^_p_*= .007 | ***F* (5, 160) = 10.3,**  ***MSE* = .084,**  ***p* < .001, *η^2^_p_*= .244** | *F* (5, 160) = 0.689,  *MSE* = .084,  *p* = .626, *η^2^_p_*= .021 |
| 8 | *F* (1, 27) < 0.001,  *MSE* = .155,  *p* = .992, *η^2^_p_*< .001 | *F* (1, 27) = 4.81,  *MSE* = .090,  *p* = .037, *η^2^_p_*= .151 | ***F* (6, 162) = 7.63,**  ***MSE* = .113,**  ***p* < .001, *η^2^_p_*= .220** | *F* (1, 27) = 0.610,  *MSE* = .090,  *p* = .441, *η^2^_p_*= .022 | *F* (6, 162) = 0.945,  *MSE* = .113,  *p* = .465, *η^2^_p_*= .034 | ***F* (6, 162) = 12.5,**  ***MSE* = .111,**  ***p* < .001, *η^2^_p_*= .317** | *F* (6, 162) = 1.06,  *MSE* = .111,  *p* = .391, *η^2^_p_*= .038 |
| 12 | *F* (1, 18) = 0.035,  *MSE* = .342,  *p* = .854, *η^2^_p_*= .002 | *F* (1, 18) = 6.48,  *MSE* = .062,  *p*  = .020, *η^2^_p_*= .265 | ***F* (10, 180) = 5.88,**  ***MSE* = .097,**  ***p*  < .001, *η^2^_p_*= .246** | *F* (1, 18) = 0.454,  *MSE* = .062,  *p* = .509, *η^2^_p_*= .025 | *F* (10, 180) = 1.61,  *MSE* = .097,  *p*  = .106, *η^2^_p_*= .082 | ***F* (10, 180) = 7.43,**  ***MSE* = .084,**  ***p*  < .001, *η^2^_p_*= .292** | *F* (10, 180) = 1.94,  *MSE* = .084,  *p* = .042, *η^2^_p_*= .097 |

*Note:* SP1 was excluded since it was, by definition, always recalled. Significant main effects and interactions are presented in bold. Bonferroni corrected *p*-level = .0083.

**Appendix B6**. Experiment 1. Analyses of the IFR SPCs, shown in Figure 5, using only data from trials starting with SP1 for auditory and visual IFR conditions using FR scoring. At each LL, the data were subjected to a 2 (modality: auditory or visual) x *n-1* (SP: 1, …, *n*, where *n* is the LL) within-subjects ANOVA.

| **LL** | **Main Effects** | | **2-way interaction** | **SME (Modality advantage: SP)** |
| --- | --- | --- | --- | --- |
|  | **Modality** | **SP** | **Modality x SP** |  |
| 4 | *F* (1, 18) = 0.169, *MSE* = .033,  *p* = .686, *η^2^_p_* = .009 | *F* (2, 36) = 4.24, *MSE* = .026,  *p*  = .022, *η^2^_p_* = .191 | *F* (2, 36) = 2.78, *MSE* = .035,  *p*  = .075, *η^2^_p_* = .134 | 4 |
| 5 | *F* (1, 19) = 4.49, *MSE* = .099,  *p* = .047, *η^2^_p_* = .191 | *F* (3, 57) = 3.91, *MSE* = .097,  *p*  = .013, *η^2^_p_* = .171 | ***F* (3, 57) = 9.45, *MSE* = .079,**  ***p*  < .001, *η^2^_p_* = .332** | 5 |
| 6 | *F* (1, 17) = 4.27, *MSE* = .058,  *p* = .055, *η^2^_p_* = .200 | *F* (4, 68) = 2.58, *MSE* = .110,  *p*  = .045, *η^2^_p_* = .132 | ***F* (4, 68) = 6.94, *MSE* = .109,**  ***p*  < .001, *η^2^_p_* = .290** | 6 |
| 7 | *F* (1, 14) = 4.30, *MSE* = .088,  *p* = .057, *η^2^_p_* = .235 | *F* (5, 70) = 1.74, *MSE* = .162,  *p* = .137, *η^2^_p_* = .111 | *F* (5, 70) = 2.86, *MSE* = .093,  *p* = .021, *η^2^_p_* = .170 | 6, 7 |
| 8 | *F* (1, 11) = 2.76, *MSE* = .124,  *p* = .125, *η^2^_p_* = .201 | *F* (6, 66) = 3.03, *MSE* = .147,  *p =* .011, *η^2^_p_* = .216 | ***F* (6, 66) = 5.69, *MSE* = .146,**  ***p* < .001, *η^2^_p_* = .341** | 3, 4, 7, 8 |
| 12 | *F* (1, 4) = 3.75, *MSE* = .057,  *p* = .125, *η^2^_p_* = .484 | *F* (10, 40) = 1.71, *MSE* = .160,  *p*  = .113, *η^2^_p_* = .299 | ***F* (10, 40) = 2.96, *MSE* = .141,**  ***p*  = .007, *η^2^_p_* = .426** | 9, 11, 12 |

*Note:* SP1 was excluded since it was, by definition, always recalled. Significant main effects and interactions are presented in bold; underlined values signify a significant inversion of the modality effect. Bonferroni corrected *p*-level = .0083.

**Appendix B7**. Experiment 1. Analyses of the ISR SPCs, using only data from trials starting with SP1 for auditory and visual ISR conditions using FR scoring. At each LL, the data were subjected to a 2 (modality: auditory or visual) x *n-1* (SP: 2, …, *n*, where *n* is the LL) within-subjects ANOVA.

| **LL** | **Main Effects** | | **2-way interaction** | **SME (Modality advantage: SP)** |
| --- | --- | --- | --- | --- |
|  | **Modality** | **SP** | **Modality x SP** |  |
| 4 | ***F* (1, 19) = 9.24, *MSE* = .028,**  ***p* = .007, *η^2^_p_* = .327** | *F* (2, 38) = 2.43, *MSE* = .022,  *p*  = .101, *η^2^_p_* = .113 | *F* (2, 38) = 2.26, *MSE* = .023,  *p*  = .119, *η^2^_p_* = .106 | 4 |
| 5 | ***F* (1, 19) = 9.74, *MSE* = .052,**  ***p* = .006, *η^2^_p_* = .339** | ***F* (3, 57) = 8.88, *MSE* = .042,**  ***p*  < .001, *η^2^_p_* = .319** | ***F* (3, 57) = 12.9, *MSE* = .040,**  ***p*  < .001, *η^2^_p_* = .404** | 5 |
| 6 | ***F* (1, 19) = 15.1, *MSE* = .042,**  ***p* = .001, *η^2^_p_* = .443** | ***F* (4, 76) = 13.8, *MSE* = .049,**  ***p*  < .001, *η^2^_p_* = .420** | ***F* (4, 76) = 14.6, *MSE* = .043,**  ***p*  < .001, *η^2^_p_* = .434** | 2, 6 |
| 7 | *F* (1, 18) = 8.64, *MSE* = .057,  *p* = .009, *η^2^_p_* = .324 | ***F* (5, 90) = 7.51, *MSE* = .063,**  ***p <* .001, *η^2^_p_* = .294** | ***F* (5, 90) = 9.28, *MSE* = .076,**  ***p* < .001, *η^2^_p_* = .340** | 6, 7 |
| 8 | *F* (1, 16) = 1.61, *MSE* = .068,  *p* = .223, *η^2^_p_* = .091 | ***F* (6, 96) = 6.03, *MSE* = .089,**  ***p <* .001, *η^2^_p_* = .274** | ***F* (6, 96) = 7.43, *MSE* = .087,**  ***p* < .001, *η^2^_p_* = .317** | 8 |
| 12 | *F* (1, 14) = 3.43, *MSE* = .063,  *p* = .085, *η^2^_p_* = .197 | ***F* (10, 140) = 8.06, *MSE* = .079,**  ***p*  < .001, *η^2^_p_* = .365** | ***F* (10, 140) = 4.76, *MSE* = .068,**  ***p*  < .001, *η^2^_p_* = .254** | 11, 12 |

*Note:* SP1 was excluded since it was, by definition, always recalled. Significant main effects and interactions are presented in bold. Bonferroni corrected *p*-level = .0083.

**Appendix B8**. Experiment 1. Analyses of the ISR SPCs, shown in Figure 6, using only data from trials starting with SP1 for auditory and visual ISR conditions using SR scoring. At each LL, the data were subjected to a 2 (modality: auditory or visual) x *n-1* (SP: 2, …, *n*, where *n* is the LL) within-subjects ANOVA.

| **LL** | **Main Effects** | | **2-way interaction** | **SME (Modality advantage: SP)** |
| --- | --- | --- | --- | --- |
|  | **Modality** | **SP** | **Modality x SP** |  |
| 4 | *F* (1, 19) = 5.68, *MSE* = .038,  *p* = .028, *η^2^_p_* = .230 | *F* (2, 38) = 4.54, *MSE* = .020,  *p*  = .017, *η^2^_p_* = .193 | *F* (2, 38) = 2.96, *MSE* = .023,  *p*  = .064, *η^2^_p_* = .135 | 4 |
| 5 | *F* (1, 19) = 6.17, *MSE* = .059,  *p* = .022, *η^2^_p_* = .245 | ***F* (3, 57) = 17.8, *MSE* = .047,**  ***p*  < .001, *η^2^_p_* = .484** | ***F* (3, 57) = 15.7, *MSE* = .040,**  ***p*  < .001, *η^2^_p_* = .453** | 3, 5 |
| 6 | ***F* (1, 19) = 11.0, *MSE* = .069,**  ***p* = .004, *η^2^_p_* = .367** | ***F* (4, 76) = 22.3, *MSE* = .044,**  ***p*  < .001, *η^2^_p_* = .540** | ***F* (4, 76) = 16.0, *MSE* = .039,**  ***p*  < .001, *η^2^_p_* = .457** | 6 |
| 7 | *F* (1, 18) = 6.32, *MSE* = .106,  *p* = .022, *η^2^_p_* = .260 | ***F* (5, 90) = 24.8, *MSE* = .057,**  ***p* < .001, *η^2^_p_* = .580** | ***F* (5, 90) = 8.25, *MSE* = .074,**  ***p* < .001, *η^2^_p_* = .314** | 6, 7 |
| 8 | *F* (1, 16) = 3.69, *MSE* = .138,  *p* = .073, *η^2^_p_* = .188 | ***F* (6, 96) = 13.3, *MSE* = .062,**  ***p* < .001, *η^2^_p_* = .454** | ***F* (6, 96) = 6.76, *MSE* = .063,**  ***p* < .001, *η^2^_p_* = .297** | 7, 8 |
| 12 | ***F* (1, 14) = 12.1, *MSE* = .046,**  ***p* = .004, *η^2^_p_* = .463** | ***F* (10, 140) = 14.9, *MSE* = .055,**  ***p*  < .001, *η^2^_p_* = .516** | ***F* (10, 140) = 5.41, *MSE* = .043,**  ***p*  < .001, *η^2^_p_* = .279** | 11, 12 |

*Note:* SP1 was excluded since it was, by definition, almost always recalled. Significant main effects and interactions are presented in bold; underlined values signify a significant inversion of the modality effect. Bonferroni corrected *p*-level = .0083.

**Appendix B9.** Experiment 1. Analyses of the SPCs, shown in Figures 7 and 8, using only data from trials starting with one of the last four words for the auditory and visual IFR and ISR conditions using FR scoring. At each LL, the data were subjected to a 2 (task: IFR or ISR) x 2 (modality: auditory or visual) *n* (SP: 1, …, *n*, where *n* is the LL) mixed ANOVA.

| **LL** | **Main Effects** | | | **2-way interactions** | | | **3-way interaction** |
| --- | --- | --- | --- | --- | --- | --- | --- |
|  | **Task** | **Modality** | **SP** | **Task x Modality** | **Task x SP** | **Modality x SP** | **Task x Modality x SP** |
| 4 |  |  |  |  |  |  |  |
| 5 | *F* (1, 10) = 3.73,  *MSE* = .203,  *p* = .082, *η^2^_p_* = .272 | *F* (1, 10) = 0.167,  *MSE* = .104,  *p* = .692, *η^2^_p_* = .016 | *F* (4, 40) = 3.63,  *MSE* = .096,  *p*  = .013, *η^2^_p_* = .267 | *F* (1, 10) = 0.327,  *MSE* = .104,  *p* = .580, *η^2^_p_* = .032 | *F* (4, 40) = 1.50,  *MSE* = .096,  *p*  = .220, *η^2^_p_* = .131 | *F* (4, 40) = 0.888,  *MSE* = .219,  *p*  = .480, *η^2^_p_* = .082 | *F* (4, 40) = 0.208,  *MSE* = .219,  *p* = .933, *η^2^_p_* = .020 |
| 6 | *F* (1, 16) = 3.40,  *MSE* = .218,  *p* = .084, *η^2^_p_* = .175 | *F* (1, 16) = 4.57,  *MSE* = .120,  *p*  = .048, *η^2^_p_* = .222 | ***F* (5, 80) = 6.05,**  ***MSE* = .198,**  ***p*  < .001, *η^2^_p_* = .274** | *F* (1, 16) = 0.650,  *MSE* = .120,  *p* = .432, *η^2^_p_* = .039 | *F* (5, 80) = 1.45,  *MSE* = .198,  *p*  = .215, *η^2^_p_* = .083 | *F* (5, 80) = 0.900,  *MSE* = .099,  *p*  = .486, *η^2^_p_* = .053 | *F* (5, 80) = 0.833,  *MSE* = .099,  *p* = .530, *η^2^_p_* = .050 |
| 7 | *F* (1, 14) = 8.63,  *MSE* = .089,  *p* = .011, *η^2^_p_* = .381 | *F* (1, 14) = 0.430,  *MSE* = .045,  *p* = .523, *η^2^_p_* = .030 | ***F* (6, 84) = 19.2,**  ***MSE* = .115,**  ***p* < .001, *η^2^_p_* = .578** | *F* (1, 14) = 0.430,  *MSE* = .045,  *p* = .523, *η^2^_p_* = .030 | ***F* (6, 84) = 3.00,**  ***MSE* = .115,**  ***p =* .010, *η^2^_p_* = .177** | *F* (6, 84) = 0.208,  *MSE* = .128,  *p* = .973, *η^2^_p_* = .015, | *F* (6, 84) = 0.607,  *MSE* = .128,  *p* = .724, *η^2^_p_* = .042 |
| 8 | ***F* (1, 21) = 17.2,**  ***MSE* = .070,**  ***p* < .001, *η^2^_p_* = .450** | *F* (1, 21) = 3.23,  *MSE* = .050,  *p* = .087, *η^2^_p_* = .133 | ***F* (7, 147) = 51.0,**  ***MSE* = .078,**  ***p* < .001, *η^2^_p_* = .708** | *F* (1, 21) = 1.18,  *MSE* = .050,  *p* = .291, *η^2^_p_* = .053 | *F* (7, 147) = 2.42,  *MSE* = .078,  *p* = .023, *η^2^_p_* = .103 | *F* (7, 147) = 1.03,  *MSE* = .063,  *p* = .412, *η^2^_p_* = .047 | *F* (7, 147) = 0.339,  *MSE* = .063,  *p* = .935, *η^2^_p_* = .016 |
| 12 | ***F* (1, 23) = 9.05,**  ***MSE* = .105,**  ***p* = .006, *η^2^_p_* = .282** | *F* (1, 23) = 2.25,  *MSE* = .024,  *p*  = .151, *η^2^_p_* = .089 | ***F* (11, 253) = 55.1,**  ***MSE* = .067,**  ***p*  < .001, *η^2^_p_* = .706** | *F* (1, 23) < 0.001,  *MSE* = .024,  *p* = .989, *η^2^_p <_* .001 | ***F* (11, 253) = 5.21,**  ***MSE* = .067,**  ***p*  < .001, *η^2^_p_* = .185** | *F* (11, 253) = 0.459,  *MSE* = .074,  *p*  = .927, *η^2^_p_* = .020 | *F* (11, 253) = 0.216,  *MSE* = .074,  *p* = .996, *η^2^_p_* = .009 |

*Note:* Significant main effects and interactions are presented in bold. Bonferroni corrected *p*-level = .0100.

**Appendix B10.** Experiment 1. Analyses of the SPCs, shown in Figures 7 and 8, restricted to the last four SPs, using only data from trials starting with one of the last four SPs for the auditory and visual IFR and ISR conditions using FR scoring. At each LL, the data were subjected to a 2 (task: IFR or ISR) x 2 (modality: auditory or visual) *n* (SP: 1, …, *n*, where *n* is the LL) mixed ANOVA.

| **LL** | **Main Effects** | | | **2-way interactions** | | | **3-way interaction** |
| --- | --- | --- | --- | --- | --- | --- | --- |
|  | **Task** | **Modality** | **SP** | **Task x Modality** | **Task x SP** | **Modality x SP** | **Task x Modality x SP** |
| 5 | *F* (1, 10) = 1.85,  *MSE* = .133,  *p* = .204, *η^2^_p_* = .156 | *F* (1, 10) = 0.127,  *MSE* = .139,  *p* = .729, *η^2^_p_* = .013 | *F* (3, 30) = 1.88,  *MSE* = .087,  *p*  = .154, *η^2^_p_* = .158 | *F* (1, 10) = 0.127,  *MSE* = .139,  *p* = .729, *η^2^_p_* = .013 | *F* (3, 30) = 0.687,  *MSE* = .087,  *p*  = .567, *η^2^_p_* = .064 | *F* (3, 30) = 0.655,  *MSE* = .236,  *p*  = .586, *η^2^_p_* = .061 | *F* (3, 30) = 0.249,  *MSE* = .236,  *p* = .861, *η^2^_p_* = .024 |
| 6 | *F* (1, 16) = 0.206,  *MSE* = .107,  *p* = .656, *η^2^_p_* = .013 | *F* (1, 16) = 1.93,  *MSE* = .096,  *p*  = .184, *η^2^_p_* = .108 | *F* (3, 48) = 3.44,  *MSE* = .208,  *p*  = .024, *η^2^_p_* = .177 | *F* (1, 16) = 0.387,  *MSE* = .096,  *p* = .542, *η^2^_p_* = .024 | *F* (3, 48) = 0.458,  *MSE* = .208,  *p*  = .713, *η^2^_p_* = .028 | *F* (3, 48) = 0.684,  *MSE* = .117,  *p*  = .566, *η^2^_p_* = .041 | *F* (3, 48) = 0.330,  *MSE* = .117,  *p* = .804, *η^2^_p_* = .020 |
| 7 | *F* (1, 14) = 0.035,  *MSE* = .165,  *p* = .853, *η^2^_p_* = .003 | *F* (1, 14) = 0.069,  *MSE* = .101,  *p* = .797, *η^2^_p_* = .005 | ***F* (3, 42) = 16.2,**  ***MSE* = .090,**  ***p*  < .001, *η^2^_p_* = .536** | *F* (1, 14) = 0.802,  *MSE* = .101,  *p* = .386, *η^2^_p_* = .054 | *F* (3, 42) = 2.90,  *MSE* = .090,  *p*  = .046, *η^2^_p_* = .172 | *F* (3, 42) = 0.278,  *MSE* = .123,  *p*  = .841, *η^2^_p_* = .019 | *F* (3, 42) = 0.906,  *MSE* = .123,  *p* = .446, *η^2^_p_* = .061 |
| 8 | *F* (1, 21) = 0.411,  *MSE* = .117,  *p* = .529, *η^2^_p_* = .019 | *F* (1, 21) = 4.94,  *MSE* = .056,  *p* = .037, *η^2^_p_* = .190 | ***F* (3, 63) = 29.4,**  ***MSE* = .084,**  ***p*  < .001, *η^2^_p_* = .583** | *F* (1, 21) = 0.136,  *MSE* = .056,  *p* = .716, *η^2^_p_* = .006 | *F* (3, 63) = 1.56,  *MSE* = .084,  *p*  = .208, *η^2^_p_* = .069 | *F* (3, 63) = 1.11,  *MSE* = .075,  *p*  = .351, *η^2^_p_* = .050 | *F* (3, 63) = 0.281,  *MSE* = .075,  *p* = .839, *η^2^_p_* = .013 |
| 12 | *F* (1, 23) = 4.36,  *MSE* = .140,  *p* = .048, *η^2^_p_* = .159 | *F* (1, 23) = 3.16,  *MSE* = .068,  *p*  = .088, *η^2^_p_* = .121 | ***F* (3, 69) = 20.0,**  ***MSE* = .068,**  ***p*  < .001, *η^2^_p_* = .465** | *F* (1, 23) = 0.437,  *MSE* = .068,  *p* = .515, *η^2^_p_* = .019 | *F* (3, 69) = 2.60,  *MSE* = .068,  *p*  = .059, *η^2^_p_* = .101 | *F* (3, 69) = 0.373,  *MSE* = .105,  *p*  = .773, *η^2^_p_* = .016 | *F* (3, 69) = 0.236,  *MSE* = .105,  *p* = .871, *η^2^_p_* = .010 |

*Note:* Only the last four SPs were included in these analyses to counteract for the fact that ISR participants are not allowed to recall earlier SPs. Significant main effects and interactions are presented in bold. Bonferroni corrected *p*-level = .0100.

**Appendix B11**. Experiment 1. Analyses of the IFR SPCs, shown in Figure 7, using only data from trials starting with one of the last four words for auditory and visual IFR using FR scoring. At each LL, the data were subjected to a 2 (modality: auditory or visual) x *n* (SP: 1, …, *n*, where *n* is the LL) within-subjects ANOVA.

| **LL** | **Main Effects** | | **2-way interaction** | **SME (Modality advantage: SP)** |
| --- | --- | --- | --- | --- |
|  | **Modality** | **SP** | **Modality x SP** |  |
| 4 | *F* (1, 4) = 0.091, *MSE* = .069,  *p* = .778, *η^2^_p_* = .022 | *F* (3, 12) = 0.385, *MSE* = .135,  *p*  = .766, *η^2^_p_* = .088 | *F* (3, 12) = 0.714, *MSE* = .073,  *p*  = .562, *η^2^_p_* = .152 | - |
| 5 | *F* (1, 8) = 0.039, *MSE* = .072,  *p* = .849, *η^2^_p_* = .005 | *F* (4, 32) = 2.26, *MSE* = .080,  *p*  = .084, *η^2^_p_* = .220 | *F* (4, 32) = 1.40, *MSE* = .172,  *p*  = .258, *η^2^_p_* = .149 | - |
| 6 | *F* (1, 13) = 2.20, *MSE* = .109,  *p* = .162, *η^2^_p_* = .145 | *F* (5, 65) = 2.61, *MSE* = .180,  *p*  = .032, *η^2^_p_* = .167 | *F* (5, 65) = 1.41, *MSE* = .098,  *p*  = .231, *η^2^_p_* = .098 | 6 |
| 7 | *F* (1, 12) < 0.001, *MSE* = .048,  *p* = 1.00, *η^2^_p_* < .001 | ***F* (6, 72) = 9.75, *MSE* = .125,**  ***p* < .001, *η^2^_p_* = .448** | *F* (6, 72) = 0.734, *MSE* = .135,  *p* = .624, *η^2^_p_* = .058 | - |
| 8 | *F* (1, 16) = 0.416, *MSE* = .058,  *p* = .528, *η^2^_p_* = .025 | ***F* (7, 112) = 32.9, *MSE* = .083,**  ***p* < .001, *η^2^_p_* = .673** | *F* (7, 112) = 0.879, *MSE* = .066,  *p* = .525, *η^2^_p_* = .052 | - |
| 12 | *F* (1, 17) = 1.53, *MSE* = .031,  *p* = .234, *η^2^_p_* = .082 | ***F* (11, 187) = 29.2, *MSE* = .080,**  ***p*  < .001, *η^2^_p_* = .632** | *F* (11, 187) = 0.685, *MSE* = .086,  *p*  = .752, *η^2^_p_* = .039 | - |

*Note:* Significant main effects and interactions are presented in bold. Bonferroni corrected *p*-level = .0083.

**Appendix B12.** Experiment 1. Analyses of the ISR SPCs, shown in Figure 8, using only data from trials starting with one of the last four words for auditory and visual ISR using FR scoring. At each LL, the data were subjected to a 2 (modality: auditory or visual) x *n* (SP: 1, …, *n*, where *n* is the LL) within-subjects ANOVA.

| **LL** | **Main Effects** | | **2-way interaction** | **SME (Modality advantage: SP)** |
| --- | --- | --- | --- | --- |
|  | **Modality** | **SP** | **Modality x SP** |  |
| 4 |  |  |  |  |
| 5 | *F* (1, 2) = 0.143, *MSE* = .233,  *p* = .742, *η^2^_p_* = .067 | *F* (4, 8) = 1.68, *MSE* = .160,  *p*  = .247, *η^2^_p_* = .456 | *F* (4, 8) = 0.197, *MSE* = .405,  *p*  = .993, *η^2^_p_* = .090 | - |
| 6 | *F* (1, 3) = 2.00, *MSE* = .167,  *p* = .252, *η^2^_p_* = .400 | *F* (5, 15) = 2.97, *MSE* = .276,  *p*  = .046, *η^2^_p_* = .497 | *F* (5, 95) = 0.680, *MSE* = .104,  *p*  = .645, *η^2^_p_* = .185 | - |
| 7 | *F* (1, 2) = 1.00, *MSE* = .024,  *p* = .423, *η^2^_p_* = .333 | ***F* (6, 12) = 23.4, *MSE* = .055,**  ***p* < .001, *η^2^_p_* = .921** | *F* (6, 12) = 0.497, *MSE* = .083,  *p* = .799, *η^2^_p_* = .199 | - |
| 8 | *F* (1, 5) = 5.99, *MSE* = .023,  *p* = .058, *η^2^_p_* = .545 | ***F* (7, 35) = 29.1, *MSE* = .064,**  ***p* < .001, *η^2^_p_* = .853** | *F* (7, 35) = 0.728, *MSE* = .052,  *p* = .650, *η^2^_p_* = .127 | 6 |
| 12 | *F* (1, 6) = 4.60, *MSE* = .004,  *p* = .076, *η^2^_p_* = .434 | ***F* (11, 66) = 60.8, *MSE* = .031,**  ***p*  < .001, *η^2^_p_* = .910** | *F* (11, 66) = 0.298, *MSE* = .040,  *p*  = .984, *η^2^_p_* = .047 | - |

*Note:* Significant main effects and interactions are presented in bold. Bonferroni corrected *p*-level = .0100.

**Appendix B13**. Experiment 2. Analyses of the SPCs for the read aloud and visual IFR and ISR conditions using FR scoring. At each LL, the data were subjected to a 2 (task: IFR or ISR) x 2 (modality: read aloud or visual) x *n* (SP: 1, …, *n*, where *n* is the LL) mixed ANOVA.

| **LL** | **Main Effects** | | | **2-way interactions** | | | **3-way interaction** |
| --- | --- | --- | --- | --- | --- | --- | --- |
|  | **Task** | **Modality** | **SP** | **Task x Modality** | **Task x SP** | **Modality x SP** | **Task x Modality x SP** |
| 2 | *F* (1, 38) = 0.196,  *MSE* = .005,  *p* = .661, *η^2^_p_* = .005 | *F* (1, 38) < 0.001,  *MSE* = .004,  *p* = 1.00, *η^2^_p_* < .001 | *F* (1, 38) = 1.15,  *MSE* = .003,  *p*  = .290, *η^2^_p_* = .029 | *F* (1, 38) = 1.12,  *MSE* = .004,  *p* = .297, *η^2^_p_* = .029 | *F* (1, 38) = 1.15,  *MSE* = .003,  *p*  = .290, *η^2^_p_* = .029 | *F* (1, 38) = 6.84,  *MSE* = .001,  *p*  = .013, *η^2^_p_* = .153 | *F* (1, 38) = 0.760,  *MSE* = .001,  *p* = .389, *η^2^_p_* = .020 |
| 4 | *F* (1, 38) = 1.55,  *MSE* = .032,  *p* = .221, *η^2^_p_* = .039 | *F* (1, 38) = 6.88,  *MSE* = .019,  *p* = .013, *η^2^_p_* = .153 | ***F* (3, 114) = 8.88,**  ***MSE* = .015,**  ***p*  < .001, *η^2^_p_* = .189** | *F* (1, 38) = 1.32,  *MSE* = .019,  *p* = .259, *η^2^_p_* = .033 | *F* (3, 114) = 0.554,  *MSE* = .015,  *p*  = .646, *η^2^_p_* = .014 | ***F* (3, 114) = 6.71,**  ***MSE* = .011,**  ***p*  < .001, *η^2^_p_* = .150** | *F* (3, 114) = 0.449,  *MSE* = .011,  *p* = .719, *η^2^_p_* = .012 |
| 5 | *F* (1, 38) = 0.848,  *MSE* = .092,  *p* = .363, *η^2^_p_* = .022, | ***F* (1, 38) = 8.13,**  ***MSE* = .033,**  ***p* = .007, *η^2^_p_* = .176** | ***F* (4, 152) = 9.79,**  ***MSE* = .048,**  ***p*  < .001, *η^2^_p_* = .205** | *F* (1, 38) = 0.301,  *MSE* = .033,  *p* = .587, *η^2^_p_* = .008 | *F* (4, 152) = 0.709,  *MSE* = .048,  *p*  = .587, *η^2^_p_* = .018 | ***F* (4, 152) = 11.6,**  ***MSE* = .032,**  ***p*  < .001, *η^2^_p_* = .234** | *F* (4, 152) = 0.730,  *MSE* = .032,  *p* = .730, *η^2^_p_* = .013 |
| 6 | *F* (1, 38) = 0.096,  *MSE* = .170,  *p* = .758, *η^2^_p_* = .003 | ***F* (1, 38) = 6.21,**  ***MSE* = .058,**  ***p*  = .017, *η^2^_p_* = .140** | ***F* (5, 190) = 4.91,**  ***MSE* = .063,**  ***p*  < .001, *η^2^_p_* = .114** | *F* (1, 38) = 0.143,  *MSE* = .058,  *p* = .708, *η^2^_p_* = .004 | ***F* (5, 190) = 4.19,**  ***MSE* = .063,**  ***p*  = .001, *η^2^_p_* = .099** | ***F* (5, 190) = 7.31,**  ***MSE* = .038,**  ***p*  < .001, *η^2^_p_* = .161** | *F* (5, 190) = 2.56,  *MSE* = .038,  *p* = .029, *η^2^_p_* = .063 |
| 7 | *F* (1, 38) = 0.003,  *MSE* = .185,  *p* = .953, *η^2^_p_* < .001 | ***F* (1, 38) = 5.66,**  ***MSE* = .041,**  ***p* = .023, *η^2^_p_* = .130** | ***F* (6, 228) = 11.8,**  ***MSE* = .075,**  ***p* < .001, *η^2^_p_* = .237** | *F* (1, 38) = 1.27,  *MSE* = .041,  *p* = .267, *η^2^_p_* = .032 | ***F* (6, 228) = 3.37,**  ***MSE* = .075,**  ***p =* .003, *η^2^_p_* = .082** | ***F* (6, 228) = 8.94,**  ***MSE* = .043,**  ***p* < .001, *η^2^_p_* = .191** | *F* (6, 228) = 1.31,  *MSE* = .043,  *p* = .253, *η^2^_p_* = .033 |
| 8 | *F* (1, 38) = 0.223,  *MSE* = .161,  *p* = .639, *η^2^_p_* = .006 | *F* (1, 38) = 5.66,  *MSE* = .041,  *p* = .023, *η^2^_p_* = .130 | ***F* (7, 266) = 11.8,**  ***MSE* = .075,**  ***p* < .001, *η^2^_p_* = .237** | *F* (1, 38) = 1.27,  *MSE* = .041,  *p* = .267, *η^2^_p_* = .032 | ***F* (7, 266) = 3.37,**  ***MSE* = .075,**  ***p* = .003, *η^2^_p_* = .082** | ***F* (7, 266) = 8.94,**  ***MSE* = .043,**  ***p* < .001, *η^2^_p_* = .191** | *F* (7, 266) = 1.31,  *MSE* = .043,  *p* = .253, *η^2^_p_* = .033 |
| 12 | *F* (1, 38) = 3.29,  *MSE* = .156  *p* = .078, *η^2^_p_* = .080 | *F* (1, 38) = 6.78,  *MSE* = .024,  *p*  = .013, *η^2^_p_* = .151 | ***F* (11, 418) = 59.4,**  ***MSE* = .054,**  ***p*  < .001, *η^2^_p_* = .610** | *F* (1, 38) = 0.084,  *MSE* = .024,  *p* = .774, *η^2^_p_* = .002 | ***F* (11, 418) = 5.40,**  ***MSE* = .054,**  ***p*  < .001, *η^2^_p_* = .124** | ***F* (11, 418) = 12.6,**  ***MSE* = .037,**  ***p*  < .001, *η^2^_p_* = .250** | *F* (11, 418) = 2.81,  *MSE* = .037,  *p* = .001, *η^2^_p_* = .069 |

*Note:* Significant main effects and interactions are presented in bold. Bonferroni corrected *p*-level = .0071.

**Appendix B14.** Experiment 2. Analyses of the IFR SPCs, shown in Figure 10, using FR scoring. At each LL, the data were subjected to a 2 (modality: read aloud or visual) x *n* (SP: 1, …, *n*, where *n* is the LL) within-subjects ANOVA.

| **LL** | **Main Effects** | | **2-way interaction** | **SME (Modality advantage: SP)** |
| --- | --- | --- | --- | --- |
|  | **Modality** | **SP** | **Modality x SP** |  |
| 2 | *F* (1, 19) = 1.00, *MSE* = .002,  *p* = .330, *η^2^_p_* = .050 | *F* (1, 19) = 1.36, *MSE* = .006,  *p*  = .258, *η^2^_p_* = .067 | *F* (1, 19) = 4.75, *MSE* = .002,  *p*  = .042, *η^2^_p_* = .200 | - |
| 4 | *F* (1, 19) = 6.40, *MSE* = .021,  *p* = .020, *η^2^_p_* = .252 | *F* (3, 57) = 2.32, *MSE* = .016,  *p*  = .085, *η^2^_p_* = .109 | *F* (3, 57) = 3.31, *MSE* = .015,  *p*  = .026, *η^2^_p_* = .148 | 4 |
| 5 | *F* (1, 19) = 6.57, *MSE* = .029,  *p* = .019, *η^2^_p_* = .257 | ***F* (4, 76) = 4.94, *MSE* = .054,**  ***p*  = .001, *η^2^_p_* = .206** | ***F* (4, 76) = 8.33, *MSE* = .030,**  ***p*  < .001, *η^2^_p_* = .305** | 4, 5 |
| 6 | *F* (1, 19) = 3.35, *MSE* = .072,  *p* = .083, *η^2^_p_* = .150 | ***F* (5, 95) = 5.52, *MSE* = .067,**  ***p*  < .001, *η^2^_p_* = .225** | ***F* (5, 95) = 3.93, *MSE* = .042,**  ***p*  = .003, *η^2^_p_* = .171** | 4, 5, 6 |
| 7 | ***F* (1, 19) = 9.64, *MSE* = .026,**  ***p* = .006, *η^2^_p_* = .337** | ***F* (6, 114) = 8.26, *MSE* = .078,**  ***p* < .001, *η^2^_p_* = .303** | *F* (6, 114) = 4.01, *MSE* = .042,  *p* = .001, *η^2^_p_* = .174 | 5, 7 |
| 8 | *F* (1, 19) = 3.15, *MSE* = .031,  *p* = .092, *η^2^_p_* = .142 | ***F* (7, 133) = 26.6, *MSE* = .054,**  ***p* < .001, *η^2^_p_* = .584** | *F* (7, 133) = 1.97, *MSE* = .062,  *p* = .064, *η^2^_p_* = .094 | - |
| 12 | *F* (1, 19) = 2.56, *MSE* = .026,  *p* = .126, *η^2^_p_* = .119 | ***F* (11, 209) = 54.8, *MSE* = .042,**  ***p*  < .001, *η^2^_p_* = .743** | ***F* (11, 209) = 3.01, *MSE* = .038,**  ***p*  = .001, *η^2^_p_* = .137** | 2, 8, 12 |

*Note:* Significant main effects and interactions are presented in bold; underlined values signify a significant inversion of the modality effect. Bonferroni corrected *p*-level = .0071.

**Appendix B15**. Experiment 2. Analyses of the ISR SPCs using FR scoring. At each LL, the data were subjected to a 2 (modality: read aloud or visual) x *n* (SP: 1, …, *n*, where *n* is the LL) within-subjects ANOVA.

| **LL** | **Main Effects** | **2-way interaction** |  | **SME (Modality advantage: SP)** |
| --- | --- | --- | --- | --- |
|  | **Modality** | **SP** | **Modality x SP** |  |
| 2 | *F* (1, 19) = 0.388, *MSE* = .005,  *p* = .541, *η^2^_p_* = .020 | *F* (1, 19) < 0.001, *MSE* = .001,  *p*  = 1.00, *η^2^_p_* < .001 | *F* (1, 19) = 2.11 *MSE* = .001,  *p*  = .163, *η^2^_p_* = .100 | - |
| 4 | *F* (1, 19) = 1.22, *MSE* = .017,  *p* = .283, *η^2^_p_* = .060 | *F* (3, 57) = 7.50, *MSE* = .014,  *p*  < .001, *η^2^_p_* = .283 | *F* (3, 57) = 4.14, *MSE* = .007,  *p*  = .010, *η^2^_p_* = .179 | 4 |
| 5 | *F* (1, 19) = 2.37, *MSE* = .037,  *p* = .140, *η^2^_p_* = .111 | ***F* (4, 76) = 5.65, *MSE* = .043,**  ***p*  < .001, *η^2^_p_* = .229** | ***F* (4, 76) = 4.10, *MSE* = .034,**  ***p*  = .005, *η^2^_p_* = .177** | 5 |
| 6 | ***F* (1, 19) = 2.90, *MSE* = .045,**  ***p* = .105, *η^2^_p_* = .132** | ***F* (5, 95) = 3.42, *MSE* = .0.58,**  ***p*  = .007, *η^2^_p_* = .153** | ***F* (5, 95) = 6.14, *MSE* = .035,**  ***p*  < .001, *η^2^_p_* = .244** | 6 |
| 7 | *F* (1, 19) = 0.575, *MSE* = .056,  *p* = .458, *η^2^_p_* = .029 | ***F* (6, 114) = 6.83, *MSE* = .072,**  ***p* < .001, *η^2^_p_* = .264** | ***F* (6, 114) = 6.21, *MSE* = .044,**  ***p* < .001, *η^2^_p_* = .246** | 6, 7 |
| 8 | *F* (1, 19) = 0.012, *MSE* = .043,  *p* = .916, *η^2^_p_* = .001 | ***F* (7, 133) = 9.28, *MSE* = .072,**  ***p* < .001, *η^2^_p_* = .328** | ***F* (7, 133) = 3.97, *MSE* = .053,**  ***p* = .001, *η^2^_p_* = .173** | 2, 3, 7, 8 |
| 12 | *F* (1, 19) = 4.38, *MSE* = .023,  *p*  = .050, *η^2^_p_* = .187 | ***F* (11, 209) = 18.4, *MSE* = .067,**  ***p*  < .001, *η^2^_p_* = .491** | ***F* (11, 209) = 12.7, *MSE* = .037,**  ***p*  < .001, *η^2^_p_* = .400** | 1, 2, 3, 4, 9, 10, 11, 12 |

*Note:* Significant main effects and interactions are presented in bold; underlined values signify a significant inversion of the modality effect. Bonferroni corrected *p*-level = .0071.

**Appendix B16**. Experiment 2. Analyses of the ISR SPCs, shown in Figure 11, using SR scoring. At each LL, the data were subjected to a 2 (modality: read aloud or visual) x *n* (SP: 1, …, *n*, where *n* is the LL) within-subjects ANOVA.

| **LL** | **Main Effects** | | **2-way interaction** | **SME (Modality advantage: SP)** |
| --- | --- | --- | --- | --- |
|  | **Modality** | **SP** | **Modality x SP** |  |
| 2 | *F* (1, 19) = 0.388, *MSE* = .005,  *p* = .541, *η^2^_p_* = .020 | *F* (1, 19) < 0.001, *MSE* = .001,  *p*  = 1.00, *η^2^_p_* < .001 | *F* (1, 19) = 2.11, *MSE* = .001,  *p*  = .163, *η^2^_p_* = .100 | - |
| 4 | *F* (1, 19) = 1.00, *MSE* = .036,  *p* = .330, *η^2^_p_* = .050 | ***F* (3, 57) = 8.76, *MSE* = .015,**  ***p*  < .001, *η^2^_p_* = .316** | ***F* (3, 57) = 6.22, *MSE* = .008,**  ***p*  = .001, *η^2^_p_* = .247** | 3, 4 |
| 5 | *F* (1, 19) = 0.203, *MSE* = .099,  *p* = .658, *η^2^_p_* = .011 | ***F* (4, 76) = 21.7, *MSE* = .039,**  ***p*  < .001, *η^2^_p_* = .533** | ***F* (4, 76) = 5.10, *MSE* = .044,**  ***p*  = .001, *η^2^_p_* = .212** | 5 |
| 6 | *F* (1, 19) = 0.233, *MSE* = .121,  *p* = .635, *η^2^_p_* = .012 | ***F* (5, 95) = 9.16, *MSE* = .074,**  ***p*  < .001, *η^2^_p_* = .325** | ***F* (5, 95) = 6.19, *MSE* = .039,**  ***p*  < .001, *η^2^_p_* = .246** | 6 |
| 7 | *F* (1, 19) = 0.684, *MSE* = .068,  *p* = .418, *η^2^_p_* = .035 | ***F* (6, 114) = 15.8, *MSE* = .070,**  ***p* < .001, *η^2^_p_* = .454** | ***F* (6, 114) = 7.67, *MSE* = .039,**  ***p* < .001, *η^2^_p_* = .288** | 6, 7 |
| 8 | *F* (1, 19) = 0.179, *MSE* = .085,  *p* = .677, *η^2^_p_* = .009 | ***F* (7, 133) = 24.4, *MSE* = .058,**  ***p* < .001, *η^2^_p_* = .563** | ***F* (7, 133) = 5.06, *MSE* = .043,**  ***p* < .001, *η^2^_p_* = .210** | 2, 3, 7, 8 |
| 12 | ***F* (1, 19) = 2.45, *MSE* = .035,**  ***p*  = .134, *η^2^_p_* = .114** | ***F* (11, 209) = 25.8, *MSE* = .058,**  ***p*  < .001, *η^2^_p_* = .576** | ***F* (11, 209) = 18.0, *MSE* = .024,**  ***p*  < .001, *η^2^_p_* = .486** | 1, 2, 3, 10, 11, 12 |

*Note:* Significant main effects and interactions are presented in bold; underlined values signify a significant inversion of the modality effect. Bonferroni corrected *p*-level = .0071

**Appendix B17**. Experiment 2. Analyses of the SPCs using only data from trials starting with SP1 for the read aloud and visual IFR and ISR conditions using FR scoring. At each LL, the data were subjected to a 2 (task: IFR or ISR) x 2 (modality: read aloud or visual) *n-1* (SP: 1, …, *n*, where *n* is the LL) mixed ANOVA.

| **LL** | **Main Effects** | | | **2-way interactions** | | | **3-way interaction** |
| --- | --- | --- | --- | --- | --- | --- | --- |
|  | **Task** | **Modality** | **SP** | **Task x Modality** | **Task x SP** | **Modality x SP** | **Task x Modality x SP** |
| 4 | *F* (1, 38) = 1.25,  *MSE* = .038,  *p* = .271, *η^2^_p_*= .032 | ***F* (1, 38) = 9.90,**  ***MSE* = .021,**  ***p* = .003, *η^2^_p_*= .207** | ***F* (2, 76) = 10.8,**  ***MSE* = .014,**  ***p*  < .001, *η^2^_p_*= .221** | *F* (1, 38) = 1.43,  *MSE* = .021,  *p* = .239, *η^2^_p_*= .036 | *F* (2, 76) = 0.379,  *MSE* = .014,  *p*  = .686, *η^2^_p_*= .010 | ***F* (2, 76) = 7.96,**  ***MSE* = .0313,**  ***p*  = .001, *η^2^_p_*= .173** | *F* (2, 76) = 0.783,  *MSE* = .013,  *p* = .483, *η^2^_p_*= .019 |
| 5 | *F* (1, 36) = 0.830,  *MSE* = .119,  *p* = .368, *η^2^_p_*= .023 | ***F* (1, 36) = 7.81,**  ***MSE* = .030,**  ***p* = .008, *η^2^_p_*= .178** | *F* (3, 108) = 3.33,  *MSE* = .066,  *p*  = .022, *η^2^_p_*= .085 | *F* (1, 36) = 0.289,  *MSE* = .030,  *p* = .594, *η^2^_p_*= .008 | *F* (3, 108) = 0.751,  *MSE* = .066,  *p*  = .524, *η^2^_p_*= .020 | ***F* (3, 108) = 9.61,**  ***MSE* = .049,**  ***p*  < .001, *η^2^_p_*= .211** | *F* (3, 108) = 1.39,  *MSE* = .049,  *p* = .249, *η^2^_p_*= .037 |
| 6 | *F* (1, 32) = 0.519,  *MSE* = .178,  *p* = .477, *η^2^_p_*= .016 | ***F* (1, 32) = 14.9,**  ***MSE* = .050,**  ***p*  = .001, *η^2^_p_*= .318** | *F* (4, 128) = 2.42,  *MSE* = .094,  *p*  = .051, *η^2^_p_*= .070 | *F* (1, 32) = 0.399,  *MSE* = .050,  *p* = .532, *η^2^_p_*= .012 | *F* (4, 128) = 1.43,  *MSE* = .094,  *p*  = .229, *η^2^_p_*= .043 | *F* (4, 128) = 1.25,  *MSE* = .081,  *p*  = .294, *η^2^_p_*= .038 | ***F* (4, 128) = 4.72,**  ***MSE* = .081,**  ***p* = .001, *η^2^_p_*= .129** |
| 7 | *F* (1, 31) = 0.852,  *MSE* = .299,  *p* = .363, *η^2^_p_*= .027 | *F* (1, 31) = 5.91,  *MSE* = .086,  *p* = .021, *η^2^_p_*= .160 | *F* (5, 155) = 2.45,  *MSE* = .110,  *p* = .036, *η^2^_p_*= .073 | *F* (1, 31) = 0.683,  *MSE* = .086,  *p* = .415, *η^2^_p_*= .022 | *F* (5, 155) = 1.68,  *MSE* = .110,  *p =* .143, *η^2^_p_*= .051 | ***F* (5, 155) = 5.83,**  ***MSE* = .092,**  ***p* < .001, *η^2^_p_*= .158** | *F* (5, 155) = 1.60,  *MSE* = .092,  *p* = .164, *η^2^_p_*= .049 |
| 8 | *F* (1, 27) < 0.001,  *MSE* = .245,  *p* = .988, *η^2^_p_*< .001 | *F* (1, 27) = 7.23,  *MSE* = .072,  *p* = .012, *η^2^_p_*= .211 | ***F* (6, 162) = 10.3,**  ***MSE* = .109,**  ***p* < .001, *η^2^_p_*= .276** | *F* (1, 27) = 2.49,  *MSE* = .072,  *p* = .126, *η^2^_p_*= .084 | *F* (6, 162) = 1.90,  *MSE* = .109,  *p* = .083, *η^2^_p_*= .066 | *F* (6, 162) = 2.74,  *MSE* = .117,  *p* = .015, *η^2^_p_*= .092 | *F* (6, 162) = 1.04,  *MSE* = .117,  *p* = .402, *η^2^_p_*= .037 |
| 12 | *F* (1, 14) = 0.118,  *MSE* = .249,  *p* = .737, *η^2^_p_*= .008 | ***F* (1, 14) = 9.84,**  ***MSE* = .055,**  ***p*  = .007, *η^2^_p_*= .413** | ***F* (10, 140) = 3.55,**  ***MSE* = .103,**  ***p*  < .001, *η^2^_p_*= .202** | *F* (1, 14) = 2.23,  *MSE* = .055,  *p* = .158, *η^2^_p_*= .137 | *F* (10, 140) = 1.45,  *MSE* = .103,  *p*  = .164, *η^2^_p_*= .094 | ***F* (10, 140) = 3.56,**  ***MSE* = .062,**  ***p*  < .001, *η^2^_p_*= .203** | *F* (10, 140) = 1.26,  *MSE* = .062,  *p* = .257, *η^2^_p_*= .083 |

*Note:* SP1 was excluded since it was, by definition, always recalled. Significant main effects and interactions are presented in bold. Bonferroni corrected *p*-level = .0083.

**Appendix B18**. Experiment 2. Analyses of the IFR SPCs, shown in Figure 13, using only data from trials starting with SP1 for read aloud and visual IFR conditions using FR scoring. At each LL, the data were subjected to a 2 (modality: read aloud or visual) x *n-1* (SP: 1, …, *n*, where *n* is the LL) within-subjects ANOVA.

| **LL** | **Main Effects** | | **2-way interaction** | **SME (Modality advantage: SP)** |
| --- | --- | --- | --- | --- |
|  | **Modality** | **SP** | **Modality x SP** |  |
| 4 | *F* (1, 19) = 6.99, *MSE* = .029,  *p* = .016, *η^2^_p_* = .269 | *F* (2, 38) = 4.01, *MSE* = .013,  *p*  = .026, *η^2^_p_* = .174 | *F* (2, 36) = 4.01, *MSE* = .017,  *p*  = .026, *η^2^_p_* = .174 | 4 |
| 5 | *F* (1, 17) = 4.67, *MSE* = .034,  *p* = .045, *η^2^_p_* = .216 | *F* (3, 51) = 2.06, *MSE* = .081,  *p*  = .117, *η^2^_p_* = .108 | ***F* (3, 51) = 6.62, *MSE* = .055,**  ***p*  = .001, *η^2^_p_* = .280** | 3, 4, 5 |
| 6 | *F* (1, 14) = 8.66, *MSE* = .052,  *p* = .011, *η^2^_p_* = .382 | *F* (4, 56) = 0.557, *MSE* = .112,  *p*  = .695, *η^2^_p_* = .038 | *F* (4, 56) = 3.06, *MSE* = .092,  *p*  = .024, *η^2^_p_* = .179 | 4 |
| 7 | *F* (1, 14) = 4.60, *MSE* = .091,  *p* = .050, *η^2^_p_* = .247 | *F* (5, 70) = 0.994, *MSE* = .153,  *p* = .428, *η^2^_p_* = .066 | *F* (5, 70) = 2.02, *MSE* = .123,  *p* = .086, *η^2^_p_* = .126 | 7 |
| 8 | *F* (1, 8) = 9.03, *MSE* = .052,  *p* = .017, *η^2^_p_* = .530 | ***F* (6, 48) = 5.38, *MSE* = .123,**  ***p <* .001, *η^2^_p_* = .402** | *F* (6, 48) = 0.814, *MSE* = .146,  *p* = .565, *η^2^_p_* = .092 | - |
| 12 | *F* (1, 1) = 1.47, *MSE* = .228,  *p* = .440, *η^2^_p_* = .594 | *F* (10, 10) = 1.32, *MSE* = .140,  *p*  = .337, *η^2^_p_* = .568 | *F* (10, 10) = 1.84, *MSE* = .067,  *p*  = .175, *η^2^_p_* = .648 | 10 |

*Note:* SP1 was excluded since it was, by definition, always recalled. Significant main effects and interactions are presented in bold; underlined values signify a significant inversion of the modality effect. Bonferroni corrected *p*-level = .0083.

**Appendix B19.** Experiment 2. Analyses of the ISR SPCs, using only data from trials starting with SP1 for read aloud and visual ISR conditions using FR scoring. At each LL, the data were subjected to a 2 (modality: read aloud or visual) x *n-1* (SP: 2, …, *n*, where *n* is the LL) within-subjects ANOVA.

| **LL** | **Main Effects** | | **2-way interaction** | **SME (Modality advantage: SP)** |
| --- | --- | --- | --- | --- |
|  | **Modality** | **SP** | **Modality x SP** |  |
| 4 | *F* (1, 19) = 2.92, *MSE* = .014,  *p* = .104, *η^2^_p_* = .133 | ***F* (2, 38) = 6.89, *MSE* = .015,**  ***p*  = .003, *η^2^_p_* = .266** | *F* (2, 38) = 5.01, *MSE* = .009,  *p*  = .012, *η^2^_p_* = .209 | 4 |
| 5 | *F* (1, 19) = 3.03, *MSE* = .027,  *p* = .098, *η^2^_p_* = .138 | *F* (3, 57) = 1.87, *MSE* = .052,  *p*  = .144, *η^2^_p_* = .090 | *F* (3, 57) = 3.79, *MSE* = .044,  *p*  = .015, *η^2^_p_* = .166 | 5 |
| 6 | *F* (1, 18) = 6.14, *MSE* = .048,  *p* = .023, *η^2^_p_* = .254 | ***F* (4, 72) = 4.13, *MSE* = .081,**  ***p*  < .005, *η^2^_p_* = .187** | *F* (4, 72) = 2.66, *MSE* = .073,  *p*  = .040, *η^2^_p_* = .129 | 6 |
| 7 | *F* (1, 17) = 1.49, *MSE* = .082,  *p* = .239, *η^2^_p_* = .080 | ***F* (5, 85) = 4.25, *MSE* = .074,**  ***p =* .002, *η^2^_p_* = .200** | ***F* (5, 85) = 6.81, *MSE* = .067,**  ***p* < .001, *η^2^_p_* = .286** | *3,* 6, 7 |
| 8 | *F* (1, 19) = 0.894, *MSE* = .080,  *p* = .356, *η^2^_p_* = .045 | ***F* (6, 114) = 6.47, *MSE* = .103,**  ***p <* .001, *η^2^_p_* = .254** | ***F* (6, 114) = 4.28, *MSE* = .105,**  ***p* = .001, *η^2^_p_* = .184** | 7, 8 |
| 12 | *F* (1, 13) = 7.16, *MSE* = .041,  *p* = .019, *η^2^_p_* = .355 | ***F* (10, 130) = 7.81, *MSE* = .101,**  ***p*  < .001, *η^2^_p_* = .375** | ***F* (10, 130) = 5.45 *MSE* = .062,**  ***p*  < .001, *η^2^_p_* = .295** | 2, 11, 12 |

*Note:* SP1 was excluded since it was, by definition, always recalled. Significant main effects and interactions are presented in bold; underlined values signify a significant inversion of the modality effect. Bonferroni corrected *p*-level = .0083.

**Appendix B20.** Experiment 2. Analyses of the ISR SPCs, shown in Figure 14, using only data from trials starting with SP1 for read aloud and visual ISR conditions using SR scoring. At each LL, the data were subjected to a 2 (modality: read aloud or visual) x *n-1* (SP: 2, …, *n*, where *n* is the LL) within-subjects ANOVA.

| **LL** | **Main Effects** | | **2-way interaction** | **SME (Modality advantage: SP)** |
| --- | --- | --- | --- | --- |
|  | **Modality** | **SP** | **Modality x SP** |  |
| 4 | *F* (1, 19) = 2.41, *MSE* = .032,  *p* = .137, *η^2^_p_* = .112 | ***F* (2, 38) = 7.16, *MSE* = .018,**  ***p*  = .002, *η^2^_p_* = .274** | ***F* (2, 38) = 5.50, *MSE* = .011,**  ***p*  = .008, *η^2^_p_* = .224** | 3, 4 |
| 5 | *F* (1, 19) = 1.13, *MSE* = .111,  *p* = .302, *η^2^_p_* = .056 | ***F* (3, 57) = 11.8, *MSE* = .045,**  ***p*  < .001, *η^2^_p_* = .382** | ***F* (3, 57) = 5.11, *MSE* = .048,**  ***p*  = .003, *η^2^_p_* = .212** | 5 |
| 6 | *F* (1, 18) = 0.731, *MSE* = .203,  *p* = .404, *η^2^_p_* = .039 | ***F* (4, 72) = 6.65, *MSE* = .088,**  ***p*  < .001, *η^2^_p_* = .270** | *F* (4, 72) = 2.94, *MSE* = .068,  *p*  = .026, *η^2^_p_* = .140 | 6 |
| 7 | *F* (1, 17) = 2.15, *MSE* = .140,  *p* = .161, *η^2^_p_* = .112 | ***F* (5, 85) = 11.4, *MSE* = .058,**  ***p* < .001, *η^2^_p_* = .401** | ***F* (5, 85) = 7.31, *MSE* = .053,**  ***p* < .001, *η^2^_p_* = .301** | 6, 7 |
| 8 | *F* (1, 19) = 0.100, *MSE* = .148,  *p* = .755, *η^2^_p_* = .005 | ***F* (6, 114) = 13.3, *MSE* = .078,**  ***p* < .001, *η^2^_p_* = .412** | ***F* (6, 114) = 3.92, *MSE* = .070,**  ***p* = .001, *η^2^_p_* = .171** | 8 |
| 12 | *F* (1, 13) = 3.98, *MSE* = .087,  *p* = .067, *η^2^_p_* = .234 | ***F* (10, 130) = 13.1, *MSE* = .075,**  ***p*  < .001, *η^2^_p_* = .501** | ***F* (10, 130) = 8.01, *MSE* = .044,**  ***p*  < .001, *η^2^_p_* = .381** | 2, 11, 12 |

*Note:* SP1 was excluded since it was, by definition, almost always recalled. Significant main effects and interactions are presented in bold; underlined values signify a significant inversion of the modality effect. Bonferroni corrected *p*-level = .0083.

**Appendix B21**. Experiment 2. Analyses of the SPCs, shown in Figures 15 and 16, using only data from trials starting with one of the last four words for the read aloud and visual IFR and ISR conditions using FR scoring. At each LL, the data were subjected to a 2 (task: IFR or ISR) x 2 (modality: read aloud or visual) *n* (SP: 1, …, *n*, where *n* is the LL) mixed ANOVA.

| **LL** | **Main Effects** | | | **2-way interactions** | | | **3-way interaction** |
| --- | --- | --- | --- | --- | --- | --- | --- |
|  | **Task** | **Modality** | **SP** | **Task x Modality** | **Task x SP** | **Modality x SP** | **Task x Modality x SP** |
| 4 |  |  |  |  |  |  |  |
| 5 |  |  |  |  |  |  |  |
| 6 | *F* (1, 15) = 7.75,  *MSE* = .143,  *p* = .014, *η^2^_p_* = .341 | *F* (1, 15) = 3.40,  *MSE* = .097,  *p*  = .085, *η^2^_p_* = .185 | ***F* (5, 75) = 11.4,**  ***MSE* = .151,**  ***p*  < .001, *η^2^_p_* = .432** | *F* (1, 15) = 2.24,  *MSE* = .097,  *p* = .155, *η^2^_p_* = .130 | *F* (5, 75) = 2.17,  *MSE* = .151,  *p*  = .066, *η^2^_p_* = .127 | *F* (5, 75) = 1.40,  *MSE* = .125,  *p*  = .233, *η^2^_p_* = .086 | *F* (5, 75) = 1.80,  *MSE* = .125,  *p* = .123, *η^2^_p_* = .107 |
| 7 | *F* (1, 20) = 2.41,  *MSE* = .171,  *p* = .136, *η^2^_p_* = .108 | *F* (1, 20) = 0.193,  *MSE* = .029,  *p* = .665, *η^2^_p_* = .010 | ***F* (6, 120) = 15.8,**  ***MSE* = .133,**  ***p* < .001, *η^2^_p_* = .442** | *F* (1, 20) = 1.48,  *MSE* = .029,  *p* = .237, *η^2^_p_* = .069 | *F* (6, 120) = 1.68,  *MSE* = .133,  *p =* .132, *η^2^_p_* = .077 | *F* (6, 120) = 1.97,  *MSE* = .086,  *p* = .075, *η^2^_p_* = .090, | *F* (6, 120) = 0.806,  *MSE* = .086,  *p* = .567, *η^2^_p_* = .039 |
| 8 | ***F* (1, 16) = 9.74,**  ***MSE* = .133,**  ***p* = .007, *η^2^_p_* = .378** | *F* (1, 16) = 0.340,  *MSE* = .055,  *p* = .568, *η^2^_p_* = .021 | ***F* (7, 112) = 18.4,**  ***MSE* = .080,**  ***p* < .001, *η^2^_p_* = .534** | *F* (1, 16) = 0.066,  *MSE* = .055,  *p* = .800, *η^2^_p_* = .004 | *F* (7, 112) = 1.09,  *MSE* = .080,  *p* = .373, *η^2^_p_* = .064 | *F* (7, 112) = 0.552,  *MSE* = .070,  *p* = .793, *η^2^_p_* = .033 | *F* (7, 112) = 0.958,  *MSE* = .070,  *p* = .465, *η^2^_p_* = .057 |
| 12 | ***F* (1, 23) = 10.6,**  ***MSE* = .176,**  ***p* = .003, *η^2^_p_* = .316** | *F* (1, 23) = 0.985,  *MSE* = .063,  *p*  = .331, *η^2^_p_* = .041 | ***F* (11, 253) = 57.8,**  ***MSE* = .054,**  ***p*  < .001, *η^2^_p_* = .715** | *F* (1, 23) = 0.480,  *MSE* = .063,  *p* = .495, *η^2^_p_* = .020 | *F* (11, 253) = 1.81,  *MSE* = .054,  *p*  = .053, *η^2^_p_* = .073 | *F* (11, 253) = 1.19,  *MSE* = .065,  *p*  = .298, *η^2^_p_* = .049 | *F* (11, 253) = 0.989,  *MSE* = .065,  *p* = .457, *η^2^_p_* = .041 |

*Note:* Significant main effects and interactions are presented in bold. Bonferroni corrected *p*-level = .0125.

**Appendix B22**. Experiment 2. Analyses of the SPCs, shown in Figures 15 and 16, restricted to the last four SPs, using only data from trials starting with one of the last four SPs for the read aloud and visual IFR and ISR conditions using FR scoring. At each LL, the data were subjected to a 2 (task: IFR or ISR) x 2 (modality: read aloud or visual) *n* (SP: 1, …, *n*, where *n* is the LL) mixed ANOVA.

| **LL** | **Main Effects** | | | **2-way interactions** | | | **3-way interaction** |
| --- | --- | --- | --- | --- | --- | --- | --- |
|  | **Task** | **Modality** | **SP** | **Task x Modality** | **Task x SP** | **Modality x SP** | **Task x Modality x SP** |
| 6 | *F* (1, 15) = 0.778,  *MSE* = .098,  *p* = .392, *η^2^_p_* = .049 | *F* (1, 15) = 6.05,  *MSE* = .094,  *p*  = .027, *η^2^_p_* = .287 | *F* (3, 45) = 2.67,  *MSE* = .187,  *p*  = .059, *η^2^_p_* = .151 | *F* (1, 15) = 0.101,  *MSE* = .094,  *p* = .755, *η^2^_p_* = .007 | *F* (3, 45) = 0.852,  *MSE* = .187,  *p*  = .473, *η^2^_p_* = .054 | *F* (3, 45) = 1.12,  *MSE* = .128,  *p*  = .350, *η^2^_p_* = .070 | *F* (3, 45) = 2.25,  *MSE* = .128,  *p* = .096, *η^2^_p_* = .130 |
| 7 | *F* (1, 20) = 0.358,  *MSE* = .078,  *p* = .556, *η^2^_p_* = .018 | *F* (1, 20) = 2.07,  *MSE* = .059,  *p* = .165, *η^2^_p_* = .094 | ***F* (3, 60) = 5.36,**  ***MSE* = .125,**  ***p*  = .002, *η^2^_p_* = .211** | *F* (1, 20) = 0.998,  *MSE* = .059,  *p* = .330, *η^2^_p_* = .048 | *F* (3, 60) = 0.874,  *MSE* = .125,  *p*  = .460, *η^2^_p_* = .042 | *F* (3, 60) = 1.55,  *MSE* = .101,  *p*  = .210, *η^2^_p_* = .072 | *F* (3, 60) = 0.737,  *MSE* = .101,  *p* = .534, *η^2^_p_* = .036 |
| 8 | *F* (1, 16) = 2.45,  *MSE* = .115,  *p* = .137, *η^2^_p_* = .133 | *F* (1, 16) = 0.081,  *MSE* = .073,  *p* = .780, *η^2^_p_* = .005 | ***F* (3, 48) = 14.0,**  ***MSE* = .069,**  ***p*  < .001, *η^2^_p_* = .467** | *F* (1, 16) = 0.945,  *MSE* = .073,  *p* = .346, *η^2^_p_* = .056 | *F* (3, 48) = 0.629,  *MSE* = .069,  *p*  = .600, *η^2^_p_* = .038 | *F* (3, 48) = 0.357,  *MSE* = .069,  *p*  = .784, *η^2^_p_* = .022 | *F* (3, 48) = 0.039,  *MSE* = .069,  *p* = .989, *η^2^_p_* = .002 |
| 12 | *F* (1, 23) = 3.10,  *MSE* = .087,  *p* = .092, *η^2^_p_* = .119 | *F* (1, 23) = 4.65,  *MSE* = .071,  *p*  = .042, *η^2^_p_* = .168 | ***F* (3, 69) = 35.0,**  ***MSE* = .070,**  ***p*  < .001, *η^2^_p_* = .604** | *F* (1, 23) = 1.09,  *MSE* = .071,  *p* = .307, *η^2^_p_* = .075 | *F* (3, 69) = 3.26,  *MSE* = .070,  *p*  = .027, *η^2^_p_* = .124 | *F* (3, 69) = 0.634,  *MSE* = .094,  *p*  = .596, *η^2^_p_* = .027 | *F* (3, 69) = 0.583,  *MSE* = .094,  *p* = .628, *η^2^_p_* = .025 |

*Note:* Only the last four SPs were included in these analyses to counteract for the fact that ISR participants are not allowed to recall earlier SPs. Significant main effects and interactions are presented in bold. Bonferroni corrected *p*-level = .0125.

**Appendix B23**. Experiment 2. Analyses of the IFR SPCs, shown in Figure 15, using only data from trials starting with one of the last four words for read aloud and visual IFR using FR scoring. At each LL, the data were subjected to a 2 (modality: read aloud or visual) x *n* (SP: 1, …, *n*, where *n* is the LL) within-subjects ANOVA.

| **LL** | **Main Effects** | | **2-way interaction** | **SME (Modality advantage: SP)** |
| --- | --- | --- | --- | --- |
|  | **Modality** | **SP** | **Modality x SP** |  |
| 4 | *F* (1, 4) = 1.00, *MSE* = .025,  *p* = .374, *η^2^_p_* = .200 | *F* (3, 12) = 3.05, *MSE* = .183,  *p*  = .070, *η^2^_p_* = .432 | *F* (3, 12) = 2.11, *MSE* = .075,  *p*  = .152, *η^2^_p_* = .345 | 3 |
| 5 | *F* (1, 5) = 0.232, *MSE* = .180,  *p* = .650, *η^2^_p_* = .044 | *F* (4, 20) = 1.35, *MSE* = .113,  *p*  = .288, *η^2^_p_* = .212 | *F* (4, 20) = 0.693, *MSE* = .122,  *p*  = .605, *η^2^_p_* = .122 | - |
| 6 | *F* (1, 10) = 0.078, *MSE* = .107,  *p* = .785, *η^2^_p_* = .008 | ***F* (5, 50) = 5.69, *MSE* = .140,**  ***p*  < .001, *η^2^_p_* = .362** | *F* (5, 50) = 2.20, *MSE* = .121,  *p*  = .069, *η^2^_p_* = .180 | 6 |
| 7 | *F* (1, 17) = 0.811, *MSE* = .030,  *p* = 0.380, *η^2^_p_* = .046 | ***F* (6, 102) = 11.6, *MSE* = .141,**  ***p* < .001, *η^2^_p_* = .406** | *F* (6, 102) = 2.37, *MSE* = .071,  *p* = .035, *η^2^_p_* = .122 | 1, 5 |
| 8 | *F* (1, 14) = 0.157, *MSE* = .056,  *p* = .698, *η^2^_p_* = .011 | ***F* (7, 98) = 24.7, *MSE* = .078,**  ***p* < .001, *η^2^_p_* = .638** | *F* (7, 98) = 1.84, *MSE* = .076,  *p* = .088, *η^2^_p_* = .116 | 3 |
| 12 | *F* (1, 18) = 0.095, *MSE* = .061,  *p* = .761, *η^2^_p_* = .005 | ***F* (11, 198) = 57.4, *MSE* = .051,**  ***p*  < .001, *η^2^_p_* = .761** | *F* (11, 198) = 1.77, *MSE* = .052,  *p*  = .062, *η^2^_p_* = .089 | - |

*Note:* Significant main effects and interactions are presented in bold; underlined values signify a significant inversion of the modality effect. Bonferroni corrected *p*-level = .0083.

**Appendix B24**. Experiment 2. Analyses of the ISR SPCs, shown in Figure 16, using only data from trials starting with one of the last four words for read aloud and visual ISR using FR scoring. At each LL, the data were subjected to a 2 (modality: read aloud or visual) x *n* (SP: 1, …, *n*, where *n* is the LL) within-subjects ANOVA.

| **LL** | **Main Effects** | | **2-way interaction** | **SME (Modality advantage: SP)** |
| --- | --- | --- | --- | --- |
|  | **Modality** | **SP** | **Modality x SP** |  |
| 6 | *F* (1, 5) = 5.29, *MSE* = .079,  *p* = .070, *η^2^_p_* = .514 | ***F* (5, 25) = 6.66, *MSE* = .172,**  ***p*  < .001, *η^2^_p_* = .571** | *F* (5, 25) = 1.23, *MSE* = .135,  *p*  = .325, *η^2^_p_* = .197 | 5 |
| 7 | *F* (1, 3) = 1.00, *MSE* = .024,  *p* = .391, *η^2^_p_* = .250 | ***F* (6, 18) = 12.2, *MSE* = .087,**  ***p* < .001, *η^2^_p_* = .802** | *F* (6, 18) = 0.633, *MSE* = .170,  *p* = .702, *η^2^_p_* = .174 | - |
| 8 | *F* (1, 2) = 0.231, *MSE* = .051,  *p* = .678, *η^2^_p_* = .103 | ***F* (7, 14) = 5.87, *MSE* = .093,**  ***p* = .002, *η^2^_p_* = .746** | *F* (7, 14) = 1.19, *MSE* = .030,  *p* = .370, *η^2^_p_* = .372 | 3 |
| 12 | *F* (1, 5) = 0.875, *MSE* = .067,  *p* = .392, *η^2^_p_* = .149 | ***F* (11, 55) = 18.2, *MSE* = .066,**  ***p*  < .001, *η^2^_p_* = .784** | *F* (11, 55) = 0.563, *MSE* = .115,  *p*  = .850, *η^2^_p_* = .101 | - |

*Note:* Significant main effects and interactions are presented in bold; underlined values signify a significant inversion of the modality effect. Bonferroni corrected *p*-level = .0125.

**Supplementary Appendices C1-C4: Probability of First Recall Data**

**Experiment 1.** Supplementary Appendices for Experiment 1 detailing the distribution of the first words recalled on each trial for the auditory and visual IFR (Appendix C1) and ISR (Appendix C2) conditions of Experiment 1 as a function of the words’ serial position and list length.

**Experiment 2.** Supplementary Appendices for Experiment 2 detailing the distribution of the first words recalled on each trial for the visual read aloud and visual IFR (Appendix C3) and ISR (Appendix C4) conditions of Experiment 2 as a function of the words’ serial position and list length.

**Appendix C1**. Experiment 1. The distribution of the first words recalled on each trial for the auditory and visual IFR conditions as a function of the words’ serial position and list length.

|  | List length | | | | | | |
| --- | --- | --- | --- | --- | --- | --- | --- |
| Serial position | 2 | 4 | 5 | 6 | 7 | 8 | 12 |
|  | IFR Auditory | | | | | | |
| 1 | *93* | *82* | *69* | *61* | *45* | *39* | *17* |
| 2 | **3** | **7** | **7** | 3 | 8 | 3 | 5 |
| 3 |  | **5** | **7** | **3** | 4 | 2 | 1 |
| 4 |  | **3** | **9** | **6** | **3** | 1 | 1 |
| 5 |  |  | **5** | **8** | **6** | **6** | 3 |
| 6 |  |  |  | **12** | **13** | **15** | 3 |
| 7 |  |  |  |  | **16** | **11** | 2 |
| 8 |  |  |  |  |  | **17** | 8 |
| 9 |  |  |  |  |  |  | **6** |
| 10 |  |  |  |  |  |  | **11** |
| 11 |  |  |  |  |  |  | **18** |
| 12 |  |  |  |  |  |  | **23** |
| Void trial / Error on first word | 4 | 3 | 3 | 7 | 5 | 6 | 2 |
| *Total* | 100 | 100 | 100 | 100 | 100 | 100 | 100 |
|  |  |  |  |  |  |  |  |
|  | IFR Visual | | | | | | |
| 1 | *96* | *83* | *68* | *50* | *46* | *25* | *14* |
| 2 | **1** | **10** | **6** | 3 | 5 | 3 | 0 |
| 3 |  | **0** | **5** | **9** | 5 | 6 | 4 |
| 4 |  | **3** | **8** | **9** | **5** | 3 | 0 |
| 5 |  |  | **11** | **13** | **9** | **11** | 1 |
| 6 |  |  |  | **13** | **11** | **12** | 5 |
| 7 |  |  |  |  | **18** | **20** | 2 |
| 8 |  |  |  |  |  | **18** | 7 |
| 9 |  |  |  |  |  |  | **7** |
| 10 |  |  |  |  |  |  | **15** |
| 11 |  |  |  |  |  |  | **23** |
| 12 |  |  |  |  |  |  | **19** |
| Void trial / Error on first word | 3 | 4 | 2 | 3 | 1 | 2 | 3 |
| *Total* | 100 | 100 | 100 | 100 | 100 | 100 | 100 |

*Note*: Values in italics represent the frequency of trials in which the first word recalled was from SP1; the bold values represent the frequency of trials in which the first word recalled was from one of the last four serial positions.

**Appendix C2**. Experiment 1. The distribution of the first words recalled on each trial for the auditory and visual ISR conditions as a function of the words’ serial position and LL.

|  | List length | | | | | | |
| --- | --- | --- | --- | --- | --- | --- | --- |
| Serial position | 2 | 4 | 5 | 6 | 7 | 8 | 12 |
|  | ISR Auditory | | | | | | |
| 1 | *99* | *91* | *89* | *82* | *78* | *60* | *48* |
| 2 | **0** | **2** | **2** | 3 | 5 | 6 | 2 |
| 3 |  | **1** | **4** | **3** | 3 | 4 | 2 |
| 4 |  | **0** | **0** | **4** | **4** | 5 | 2 |
| 5 |  |  | **0** | **3** | **3** | **6** | 2 |
| 6 |  |  |  | **0** | **1** | **8** | 2 |
| 7 |  |  |  |  | **0** | **6** | 6 |
| 8 |  |  |  |  |  | **0** | 6 |
| 9 |  |  |  |  |  |  | **11** |
| 10 |  |  |  |  |  |  | **7** |
| 11 |  |  |  |  |  |  | **6** |
| 12 |  |  |  |  |  |  | **0** |
| Void trial / Error on first word | 1 | 6 | 5 | 5 | 6 | 5 | 6 |
| *Total* | 100 | 100 | 100 | 100 | 100 | 100 | 100 |
|  |  |  |  |  |  |  |  |
|  | ISR Visual | | | | | | |
| 1 | *98* | *98* | *85* | *87* | *76* | *66* | *51* |
| 2 | **1** | **1** | **6** | 3 | 1 | 6 | 4 |
| 3 |  | **0** | **2** | **3** | 4 | 2 | 3 |
| 4 |  | **0** | **5** | **0** | **3** | 8 | 2 |
| 5 |  |  | **0** | **2** | **7** | **5** | 4 |
| 6 |  |  |  | **2** | **3** | **1** | 2 |
| 7 |  |  |  |  | **1** | **4** | 2 |
| 8 |  |  |  |  |  | **3** | 4 |
| 9 |  |  |  |  |  |  | **11** |
| 10 |  |  |  |  |  |  | **3** |
| 11 |  |  |  |  |  |  | **2** |
| 12 |  |  |  |  |  |  | **2** |
| Void trial / Error on first word | 1 | 1 | 2 | 3 | 5 | 5 | 10 |
| *Total* | 100 | 100 | 100 | 100 | 100 | 100 | 100 |

*Note*: Values in italics represent the frequency of trials in which the first word recalled was from SP1; the bold values represent the frequency of trials in which the first word recalled was from one of the last four serial positions

**Appendix C3.** Experiment 2. The distribution of the first words recalled on each trial for the read aloud and visual IFR conditions as a function of the words’ serial position and list length.

|  | List length | | | | | | |
| --- | --- | --- | --- | --- | --- | --- | --- |
| Serial position | 2 | 4 | 5 | 6 | 7 | 8 | 12 |
|  | IFR Read aloud | | | | | | |
| 1 | *93* | *87* | *75* | *56* | *34* | *28* | *9* |
| 2 | **6** | **3** | **8** | 4 | 3 | 2 | 1 |
| 3 |  | **3** | **2** | **1** | 3 | 2 | 0 |
| 4 |  | **5** | **4** | **11** | **3** | 4 | 0 |
| 5 |  |  | **10** | **12** | **18** | **4** | 0 |
| 6 |  |  |  | **15** | **17** | **10** | 2 |
| 7 |  |  |  |  | **19** | **24** | 2 |
| 8 |  |  |  |  |  | **25** | 6 |
| 9 |  |  |  |  |  |  | **7** |
| 10 |  |  |  |  |  |  | **10** |
| 11 |  |  |  |  |  |  | **31** |
| 12 |  |  |  |  |  |  | **28** |
| Void trial / Error on first word | 1 | 2 | 1 | 1 | 3 | 1 | 4 |
| *Total* | 100 | 100 | 100 | 100 | 100 | 100 | 100 |
|  |  |  |  |  |  |  |  |
|  | IFR Visual | | | | | | |
| 1 | *98* | *90* | *71* | *52* | *44* | *34* | *9* |
| 2 | **2** | **6** | **9** | 5 | 1 | 3 | 2 |
| 3 |  | **2** | **1** | **7** | 2 | 2 | 3 |
| 4 |  | **2** | **8** | **8** | **6** | 1 | 0 |
| 5 |  |  | **8** | **12** | **9** | **9** | 1 |
| 6 |  |  |  | **12** | **18** | **7** | 0 |
| 7 |  |  |  |  | **16** | **16** | 4 |
| 8 |  |  |  |  |  | **25** | 3 |
| 9 |  |  |  |  |  |  | **14** |
| 10 |  |  |  |  |  |  | **9** |
| 11 |  |  |  |  |  |  | **26** |
| 12 |  |  |  |  |  |  | **27** |
| Void trial / Error on first word | 0 | 0 | 3 | 4 | 4 | 3 | 2 |
| *Total* | 100 | 100 | 100 | 100 | 100 | 100 | 100 |

*Note*: Values in italics represent the frequency of trials in which the first word recalled was from SP1; the bold values represent the frequency of trials in which the first word recalled was from one of the last four serial positions.

**Appendix C4**. Experiment 2. The distribution of the first words recalled on each trial for the read aloud and visual ISR conditions as a function of the words’ serial position and LL.

|  | List length | | | | | | |
| --- | --- | --- | --- | --- | --- | --- | --- |
| Serial position | 2 | 4 | 5 | 6 | 7 | 8 | 12 |
|  | ISR Read aloud | | | | | | |
| 1 | *99* | *96* | *88* | *72* | *65* | *65* | *38* |
| 2 | **1** | **2** | **5** | 9 | 7 | 2 | 3 |
| 3 |  | **0** | **3** | **5** | 8 | 4 | 3 |
| 4 |  | **0** | **1** | **5** | **3** | 6 | 2 |
| 5 |  |  | **0** | **2** | **9** | **9** | 4 |
| 6 |  |  |  | **1** | **3** | **6** | 4 |
| 7 |  |  |  |  | **1** | **2** | 5 |
| 8 |  |  |  |  |  | **1** | 12 |
| 9 |  |  |  |  |  |  | **9** |
| 10 |  |  |  |  |  |  | **9** |
| 11 |  |  |  |  |  |  | **5** |
| 12 |  |  |  |  |  |  | **1** |
| Void trial / Error on first word | 0 | 2 | 3 | 6 | 4 | 5 | 5 |
| *Total* | 100 | 100 | 100 | 100 | 100 | 100 | 100 |
|  |  |  |  |  |  |  |  |
|  | ISR Visual | | | | | | |
| 1 | *99* | *99* | *91* | *79* | *76* | *72* | *62* |
| 2 | **0** | **1** | **4** | 9 | 7 | 7 | 6 |
| 3 |  | **0** | **0** | **5** | 4 | 4 | 3 |
| 4 |  | **0** | **1** | **0** | **2** | 3 | 3 |
| 5 |  |  | **0** | **2** | **4** | **7** | 3 |
| 6 |  |  |  | **2** | **2** | **1** | 2 |
| 7 |  |  |  |  | **1** | **2** | 5 |
| 8 |  |  |  |  |  | **3** | 3 |
| 9 |  |  |  |  |  |  | **4** |
| 10 |  |  |  |  |  |  | **4** |
| 11 |  |  |  |  |  |  | **1** |
| 12 |  |  |  |  |  |  | **1** |
| Void trial / Error on first word | 1 | 0 | 4 | 3 | 4 | 1 | 3 |
| *Total* | 100 | 100 | 100 | 100 | 100 | 100 | 100 |

*Note*: Values in italics represent the frequency of trials in which the first word recalled was from SP1; the bold values represent the frequency of trials in which the first word recalled was from one of the last four serial positions

**Supplementary Appendix D: Analyses comparing Experiment 1 Auditory with Experiment 2 Read aloud conditions**

The analyses in Supplementary Appendix D examined whether performance in the auditory condition of Experiment 1 differed from the read aloud condition of Experiment 2. The data were considered in three different analyses: serial position curves, probability of first recall (PFR), and resultant serial position curves.

**Analyses of the serial position curves of all the data.** Figure D1 shows the serial position curves for IFR auditory and read aloud conditions using FR scoring (Panels A and B) and for ISR auditory and read aloud conditions using SR scoring (Panels C and D). The seven list lengths are split over two panels to improve clarity. Within both tasks the auditory and read aloud conditions show a high degree of similarity.

-----------------------------------

--Figure D1 about here—

------------------------------------

The serial position curves were analysed separately for each task by a series of 2 (modality: auditory or read aloud) x *n* (serial position, where *n* is the list length) mixed ANOVAs; full statistical analyses for each list length can be found in Table D1 (IFR using FR scoring and ISR using SR scoring). To summarise, for both IFR and ISR there is no difference in recall between the auditory and read aloud conditions. Due to the very high degree of similarity between the auditory and read aloud conditions we did not conduct the correlation analysis reported in Experiment 1 and 2.

-----------------------------------

--Table D1 about here—

------------------------------------

**The PFR data.** The main PFR findings are summarised in Figure D2 (Panels A and B: IFR; Panels C and D: ISR). The PFR categories are split over two panels to improve clarity. The complete set of values for these figures can be found in Supplementary Appendices C1-C4. There appears to be a larger difference between the IFR auditory and read aloud comparison compared to the ISR conditions, particularly for the longer list lengths. There is a lower probability of initiating recall with the first serial position and a higher probability of initiating recall with one of the last four serial positions for the IFR read aloud condition compared to the IFR auditory condition.

-----------------------------------

--Figure D2 about here—

------------------------------------

Table D2 summarises the findings of a series of four 2 (modality: read aloud and visual) x 7 (list length: 2, 4, 5, 6, 7, 8, and 12) mixed ANOVAs that were performed on the proportion of trials where recall started with SP1, and the proportion of trials where recall started with one of the last four serial positions for both IFR and ISR. In all analyses, there was a non-significant main effect of modality and a significant main effect of list length. The interaction between modality and list length only reached significance when recall was initiated with any one of the last four words with IFR. Simple main effects showed that participants were more likely to initiate their recall with any one of the last four words in the read aloud condition compared to the auditory condition at list length 12 only (p = 0.045, all other p’s < 0.05). To summarise, for both IFR and ISR there is little, to no, difference in PFR between the auditory and read aloud conditions.

-----------------------------------

--Table D2 about here—

------------------------------------

Tables 3 and 8 within the main paper show how far back participants started their recall for the last four items over list lengths 6-12. Two Chi-squares were performed comparing auditory and read aloud data separately for IFR and ISR. Within the ISR Chi-square n-3 was grouped with n-2, and n-1 was grouped with n due to values less than five within the auditory ISR cell at position n and read aloud ISR cell at position n. Both Chi-squares revealed a non-significant difference between the auditory and read aloud conditions in the initial item output: IFR, χ^2^ (3) = 3.60, *p* = .308 and ISR χ^2^ (1) = 0.19, *p* = .660. Therefore for both IFR and ISR there were no differences in how far back participants started their recall for the last four items in the auditory and read aloud conditions.

**The effect of the first word recalled on the resultant serial position curves.** Figure D3 shows the resultant serial position curves given recall was initiated with SP1 for IFR auditory and read aloud conditions using FR scoring (Panels A and B) and for ISR auditory and read aloud conditions using SR scoring (Panels C and D). The seven list lengths are split over two panels to improve clarity. Within ISR the auditory and read aloud conditions show a high degree of similarity. The degree of similarity is lower within the IFR auditory and read aloud conditions but this is due to the lower probability of initiating recall with SP1.

-----------------------------------

--Figure D3 about here—

------------------------------------

The resultant serial position curves were analysed separately for each task by a series of 2 (modality: auditory or read aloud) x *n* (serial position, where *n* is the list length) mixed ANOVAs; full statistical analyses for each list length can be found in Table D3 (IFR using FR scoring and ISR using SR scoring) for trials in which recall was initiated with SP1. To summarise, for both IFR and ISR there is no difference in recall between the auditory and read aloud conditions.

-----------------------------------

--Table D3 about here—

------------------------------------

Turning now to the resultant serial position curves given recall was initiated with one of the last four words; Figure D4 shows the resultant serial position curves given recall was initiated with any one of the last four words for IFR auditory and read aloud conditions (Panels A and B) and for ISR auditory and read aloud conditions (Panels C and D) using FR scoring. The seven list lengths are split over two panels to improve clarity. Within both tasks the auditory and read aloud conditions show a high degree of similarity.

-----------------------------------

--Figure D4 about here—

------------------------------------

The resultant serial position curves were analysed separately for each task by a series of 2 (modality: auditory or read aloud) x *n* (serial position, where *n* is the list length) mixed ANOVAs; full statistical analyses for each list length can be found in Table D4 (IFR using FR scoring and ISR using SR scoring) for trials in which recall was initiated with any one of the last four words. There were not enough participants to perform either the IFR or ISR list length 2 ANOVA. To summarise, for both IFR and ISR there is no difference in recall between the auditory and read aloud conditions.

-----------------------------------

--Table D4 about here—

------------------------------------

**Table D1.** Analyses of the SPCs, shown in Figure 11, using FR scoring for IFR and SR scoring for ISR. At each LL, the data were subjected to a 2 (modality: auditory or read aloud) x *n* (SP: 1, …, *n*, where *n* is the LL) mixed ANOVA.

| **List length** | **Task** | **Scoring** | **Main Effects** | | **2-way interaction** |
| --- | --- | --- | --- | --- | --- |
|  |  |  | **Modality** | **SP** | **Modality x SP** |
| 2 | IFR | FR | *F* (1, 38) = 0.74, *MSE* = .024,  *p* = .395, *η^2^_p_* = .019 | *F* (1, 38) < 0.001, *MSE* = .005,  *p*  = 1.00, *η^2^_p_* < 0.001 | *F* (1, 38) = 5.85, *MSE* = .005,  *p*  = 0.021, *η^2^_p_* = 0.133 |
|  | ISR | SR | *F* (1, 38) = 3.23, *MSE* = .002,  *p* = .080, *η^2^_p_* = .078 | *F* (1, 38) = 0.69, *MSE* = .003,  *p* = .411, *η^2^_p_* = .018 | *F* (1, 38) = 2.76, *MSE* = .003,  *p* = .105, *η^2^_p_* = .068 |
| 4 | IFR | FR | *F* (1, 38) = 2.65, *MSE* = .050,  *p* = .112, *η^2^_p_* = .065 | *F* (3, 114) = 5.52, *MSE* = .017,  *p* = .001, *η^2^_p_* = .127 | *F* (3, 114) = 2.06, *MSE* = .017,  *p* = .109, *η^2^_p_* = .051 |
|  | ISR | SR | *F* (1, 38) = 0.77, *MSE* = .055,  *p* = .386, *η^2^_p_* = .020 | *F* (3, 114) = 3.31, *MSE* = .012,  *p* = .023, *η^2^_p_* = .080 | *F* (3, 114) = 1.12, *MSE* = .012,  *p* = .345, *η^2^_p_* = .029 |
| 5 | IFR | FR | *F* (1, 38) = 1.70, *MSE* = .062,  *p* = .201, *η^2^_p_* = .043 | ***F* (4, 152) = 12.55, *MSE* = .043,**  ***p* < .001, *η^2^_p_* = .248** | *F* (4, 152) = 0.82, *MSE* = .043,  *p* = .512, *η^2^_p_* = .021 |
|  | ISR | SR | *F* (1, 38) = 0.01, *MSE* = .181,  *p* = .921, *η^2^_p_* < .001 | ***F* (4, 152) = 32.19, *MSE* = .035,**  ***p* < .001, *η^2^_p_* = .459** | *F* (4, 152) = 0.09, *MSE* = .035,  *p* = .984, *η^2^_p_* = .002 |
| 6 | IFR | FR | *F* (1, 38) = 0.17, *MSE* = .078,  *p* = .680, *η^2^_p_* = .005 | ***F* (5, 190) = 14.58, *MSE* = .055,**  ***p* < .001, *η^2^_p_* = .277** | *F* (5, 190) = 1.85, *MSE* = .055,  *p* = .105, *η^2^_p_* = .046 |
|  | ISR | SR | *F* (1, 38) = 0.17, *MSE* = .218,  *p* = .681, *η^2^_p_* = .005 | ***F* (5, 190) = 24.08, *MSE* = .050,**  ***p* < .001, *η^2^_p_* = .388** | *F* (5, 190) = 0.69, *MSE* = .050,  *p* = .630, *η^2^_p_* = .018 |
| 7 | IFR | FR | *F* (1, 38) = 0.47, *MSE* = .087,  *p* = .496, *η^2^_p_* = .012 | ***F* (6, 228) = 15.11, *MSE* = .057,**  ***p* < .001, *η^2^_p_* = .285** | *F* (6, 228) = 2.99, *MSE* = .057,  *p* = .008, *η^2^_p_* = .073 |
|  | ISR | SR | *F* (1, 38) = 0.27, *MSE* = .171,  *p* = .606, *η^2^_p_* = .007 | ***F* (6, 228) = 29.03, *MSE* = .057,**  ***p* < .001, *η^2^_p_* = .433** | *F* (6, 228) = 1.23, *MSE* = .057,  *p* = .291, *η^2^_p_* = .031 |
| 8 | IFR | FR | *F* (1, 38) = 0.49, *MSE* = .043,  *p* = .488, *η^2^_p_* = .013 | ***F* (7, 266) = 40.32, *MSE* = .052,**  ***p* < .001, *η^2^_p_* = .515** | *F* (7, 266) = 0.65, *MSE* = .052,  *p* = .711, *η^2^_p_* = .017 |
|  | ISR | SR | *F* (1, 38) = 0.38, *MSE* = .133,  *p* = .544, *η^2^_p_* = .010 | ***F* (7, 266) = 31.67, *MSE* = .058,**  ***p* < .001, *η^2^_p_* = .455** | *F* (7, 266) = 0.83, *MSE* = .058,  *p* = .562, *η^2^_p_* = .021 |
| 12 | IFR | FR | *F* (1, 38) = 0.19, *MSE* = .054,  *p* = .668, *η^2^_p_* = .005 | ***F* (11, 418) = 57.47, *MSE* = .041,**  ***p* < .001, *η^2^_p_* = .602** | *F* (11, 418) = 2.09, *MSE* = .041,  *p* = .020, *η^2^_p_* = .052 |
|  | ISR | SR | *F* (1, 38) = 0.15, *MSE* = .069,  *p* = .704, *η^2^_p_* = .004 | ***F* (11, 418) = 52.61, *MSE* = .045,**  ***p* < .001, *η^2^_p_* = .581** | *F* (11, 418) = 0.47, *MSE* = .045,  *p* = .466, *η^2^_p_* = .025 |

*Note:* Significant main effects and interactions are presented in bold. Bonferroni corrected *p*-level = .0071.

**Table D2.** Summary of the PFR ANOVA analyses.

|  | *df* | *MSE* | *F* | *η^2^_p_* | *p* |
| --- | --- | --- | --- | --- | --- |
| *Probability of first recall = Serial Position 1* | | | | | |
| **IFR** | | | | | |
| Modality | 1, 38 | .204 | 0.34 | .009 | .564 |
| List length | 6, 228 | .043 | 88.01 | .678 | < .001 |
| Modality x list length | 6, 228 | .043 | 1.11 | .028 | .358 |
|  |  |  |  |  |  |
| **ISR** |  |  |  |  |  |
| Modality | 1, 38 | .194 | 0.42 | .011 | .519 |
| List length | 6, 228 | .036 | 42.20 | .526 | < .001 |
| Modality x list length | 6, 228 | .036 | 1.56 | .039 | .160 |
|  |  |  |  |  |  |
| *Probability of first recall = Last four* | | | | | |
| **IFR** |  |  |  |  |  |
| Modality | 1, 38 | .231 | 2.01 | .050 | .165 |
| List length | 6, 228 | .044 | 46.66 | .551 | < .001 |
| Modality x list length | 6, 228 | .044 | 2.25 | .056 | .040 |
|  |  |  |  |  |  |
| **ISR** |  |  |  |  |  |
| Modality | 1, 29 | .130 | 0.012 | <.001 | .913 |
| List length | 6, 174 | .025 | 14.77 | .337 | < .001 |
| Modality x list length | 6, 174 | .025 | 0.778 | .026 | .588 |

**Table D3.** Analyses of the resultant SPCs using only data from trials starting with SP1, shown in Figure 13, using FR scoring for IFR and SR scoring for ISR. At each LL, the data were subjected to a 2 (modality: auditory or read aloud) x *n* (SP: 1, …, *n*, where *n* is the LL) mixed ANOVA.

| **List length** | **Task** | **Scoring** | **Main Effects** | | **2-way interaction** |
| --- | --- | --- | --- | --- | --- |
|  |  |  | **Modality** | **SP** | **Modality x SP** |
| 4 | IFR | FR | *F* (1, 38) = 3.17, *MSE* = .052,  *p* = .083, *η^2^_p_* = .077 | ***F* (2, 76) = 6.02, *MSE* = .021,**  ***p* = .004, *η^2^_p_* = .137** | *F* (2, 76) = 2.19, *MSE* = .021,  *p* = .119, *η^2^_p_* = .054 |
|  | ISR | SR | *F* (1, 38) = 0.45, *MSE* = .037,  *p* = .507, *η^2^_p_* = .012 | *F* (2, 76) = 4.58, *MSE* = .011,  *p* = .013, *η^2^_p_* = .108 | *F* (2, 76) = 2.22, *MSE* = .011,  *p* = .116, *η^2^_p_* = .055 |
| 5 | IFR | FR | *F* (1, 37) = 1.56, *MSE* = .102,  *p* = .219, *η^2^_p_* = .040 | ***F* (3, 111) = 6.48, *MSE* = .071,**  ***p* < .001, *η^2^_p_* = .149** | *F* (3, 111) = 2.09, *MSE* = .071,  *p* = .105, *η^2^_p_* = .054 |
|  | ISR | SR | *F* (1, 38) = 0.07, *MSE* = .186,  *p* = .794, *η^2^_p_* = .002 | ***F* (3, 114) = 26.63, *MSE* = .039,**  ***p* < .001, *η^2^_p_* = .430** | *F* (3, 114) = 0.40, *MSE* = .039,  *p* = .756, *η^2^_p_* = .010 |
| 6 | IFR | FR | *F* (1, 33) = 1.70, *MSE* = .094,  *p* = .202, *η^2^_p_* = .049 | ***F* (4, 132) = 4.07, *MSE* = .100,**  ***p* = .004, *η^2^_p_* = .110** | *F* (4, 132) = 3.29, *MSE* = .100,  *p* = .013, *η^2^_p_* = .091 |
|  | ISR | SR | *F* (1, 37) = 0.04, *MSE* = .245,  *p* = .853, *η^2^_p_* = .001 | ***F* (4, 148) = 20.01, *MSE* = .065,**  ***p* < .001, *η^2^_p_* = .351** | *F* (4, 148) = 0.79, *MSE* = .065,  *p* = .533, *η^2^_p_* = .021 |
| 7 | IFR | FR | *F* (1, 31) = 0.03, *MSE* = .130,  *p* = .872, *η^2^_p_* = .001 | ***F* (5, 155) = 4.50, *MSE* = .132,**  ***p* = .001, *η^2^_p_* = .127** | *F* (5, 155) = 1.05, *MSE* = .132,  *p* = .391, *η^2^_p_* = .033 |
|  | ISR | SR | *F* (1, 36) = 0.04, *MSE* = .233,  *p* = .844, *η^2^_p_* = .001 | ***F* (5, 180) = 20.16, *MSE* = .069,**  ***p* < .001, *η^2^_p_* = .359** | *F* (5, 180) = 0.92, *MSE* = .069,  *p* = .467, *η^2^_p_* = .025 |
| 8 | IFR | FR | *F* (1, 28) = 0.23, *MSE* = .078,  *p* = .636, *η^2^_p_* = .008 | ***F* (6, 168) = 12.20, *MSE* = .132,**  ***p* < .001, *η^2^_p_* = .303** | *F* (6, 168) = 0.85, *MSE* = .132,  *p* = .530, *η^2^_p_* = .030 |
|  | ISR | SR | *F* (1, 35) < 0.001, *MSE* = .224,  *p* = .989, *η^2^_p_* < .001 | ***F* (6, 210) = 24.16, *MSE* = .066,**  ***p* < .001, *η^2^_p_* = .408** | *F* (6, 210) = 0.84, *MSE* = .066,  *p* = .542, *η^2^_p_* = .023 |
| 12 | IFR | FR | *F* (1, 15) = 0.94, *MSE* = .090,  *p* = .347, *η^2^_p_* = .059 | ***F* (10, 150) = 4.50, *MSE* = .153,**  ***p* < .001, *η^2^_p_* = .231** | *F* (10, 150) = 1.06, *MSE* = .153,  *p* = .397, *η^2^_p_* = .066 |
|  | ISR | SR | *F* (1, 29) = 0.67, *MSE* = .103,  *p* = .421, *η^2^_p_* = .022 | ***F* (10, 290) = 27.41, *MSE* = .062,**  ***p* < .001, *η^2^_p_* = .486** | *F* (10, 290) = 0.81, *MSE* = .062,  *p* = .617, *η^2^_p_* = .027 |

*Note:* SP1 was excluded since it was, by definition, always recalled. Significant main effects and interactions are presented in bold. Bonferroni corrected *p*-level = .0083.

**Table D4.** Analyses of the SPCs, shown in Figure 14, using FR scoring. At each LL, the data were subjected to a 2 (modality: auditory or read aloud) x *n* (SP: 1, …, *n*, where *n* is the LL) mixed ANOVA.

| **List length** | **Task** | **Scoring** | **Main Effects** | | **2-way interaction** |
| --- | --- | --- | --- | --- | --- |
|  |  |  | **Modality** | **SP** | **Modality x SP** |
| 4 | IFR | FR | *F* (1, 17) = 0.20, *MSE* = .122,  *p* = .659, *η^2^_p_* = .012 | *F* (3, 51) = 3.50, *MSE* = .104,  *p* = .022, *η^2^_p_* = .171 | *F* (3, 51) = 0.31, *MSE* = .104,  *p* = .820, *η^2^_p_* = .018 |
|  | ISR | SR | *F* (1, 2) = 5.00, *MSE* = .078,  *p* = .155, *η^2^_p_* = .714 | *F* (3, 6) = 2.68, *MSE* = .161,  *p* = .141, *η^2^_p_* = .572 | *F* (3, 6) = 0.36, *MSE* = .161,  *p* = .788, *η^2^_p_* = .151 |
| 5 | IFR | FR | *F* (1, 21) = 0.18, *MSE* = .159,  *p* = .677, *η^2^_p_* = .008 | ***F* (4, 84) = 5.84, *MSE* = .117,**  ***p* < .001, *η^2^_p_* = .218** | *F* (4, 84) = 0.98, *MSE* = .117,  *p* = .422, *η^2^_p_* = .045 |
|  | ISR | SR | *F* (1, 8) = 5.87, *MSE* = .106,  *p* = .042, *η^2^_p_* = .423 | ***F* (4, 32) = 5.59, *MSE* = .139,**  ***p* = .002, *η^2^_p_* = .411** | *F* (4, 32) = 0.88, *MSE* = .139,  *p* = .486, *η^2^_p_* = .099 |
| 6 | IFR | FR | *F* (1, 26) = 0.03, *MSE* = .168,  *p* = .873, *η^2^_p_* = .001 | ***F* (5, 130) = 7.89, *MSE* = .128,**  ***p* < .001, *η^2^_p_* = .233** | *F* (5, 130) = 0.87, *MSE* = .128,  *p* = .504, *η^2^_p_* = .032 |
|  | ISR | SR | *F* (1, 13) = 0.001, *MSE* = .148,  *p* = .967, *η^2^_p_* < .001 | ***F* (5, 65) = 12.81, *MSE* = .132,**  ***p* < .001, *η^2^_p_* = .496** | *F* (5, 65) = 0.47, *MSE* = .132,  *p* = .797, *η^2^_p_* = .035 |
| 7 | IFR | FR | *F* (1, 31) = 0.80, *MSE* = .109,  *p* = .379, *η^2^_p_* = .025 | ***F* (6, 186) = 18.07, *MSE* = .107,**  ***p* < .001, *η^2^_p_* = .368** | *F* (6, 186) = 1.13, *MSE* = .107,  *p* = .347, *η^2^_p_* = .035 |
|  | ISR | SR | *F* (1, 14) = 0.71, *MSE* = .116,  *p* = .415, *η^2^_p_* = .048 | ***F* (6, 84) = 20.49, *MSE* = .098,**  ***p* < .001, *η^2^_p_* = .594** | *F* (6, 84) = 1.67, *MSE* = .098,  *p* = .139, *η^2^_p_* = .106 |
| 8 | IFR | FR | *F* (1, 32) = 0.65, *MSE* = .071,  *p* = .427, *η^2^_p_* = .020 | ***F* (7, 224) = 45.96, *MSE* = .062,**  ***p* < .001, *η^2^_p_* = .590** | *F* (7, 224) = 0.68, *MSE* = .062,  *p* = .692, *η^2^_p_* = .021 |
|  | ISR | SR | *F* (1, 16) = 0.03, *MSE* = .093,  *p* = .864, *η^2^_p_* = .002 | ***F* (7, 112) = 26.09, *MSE* = .086,**  ***p* < .001, *η^2^_p_* = .620** | *F* (7, 112) = 0.89, *MSE* = .086,  *p* = .521, *η^2^_p_* = .052 |
| 12 | IFR | FR | *F* (1, 36) = 1.33, *MSE* = .092,  *p* = .257, *η^2^_p_* = .036 | ***F* (11, 396) = 47.57, *MSE* = .063,**  ***p* < .001, *η^2^_p_* = .569** | *F* (11, 396) = 1.29, *MSE* = .063,  *p* = .228, *η^2^_p_* = .035 |
|  | ISR | SR | *F* (1, 21) = 0.31, *MSE* = .042,  *p* = .581, *η^2^_p_* = .015 | ***F* (11, 231) = 62.55, *MSE* = .048,**  ***p* < .001, *η^2^_p_* = .749** | *F* (11, 231) = 0.43, *MSE* = .048,  *p* = .941, *η^2^_p_* = .020 |

*Note:* Significant main effects and interactions are presented in bold. Bonferroni corrected *p*-level = .0083.


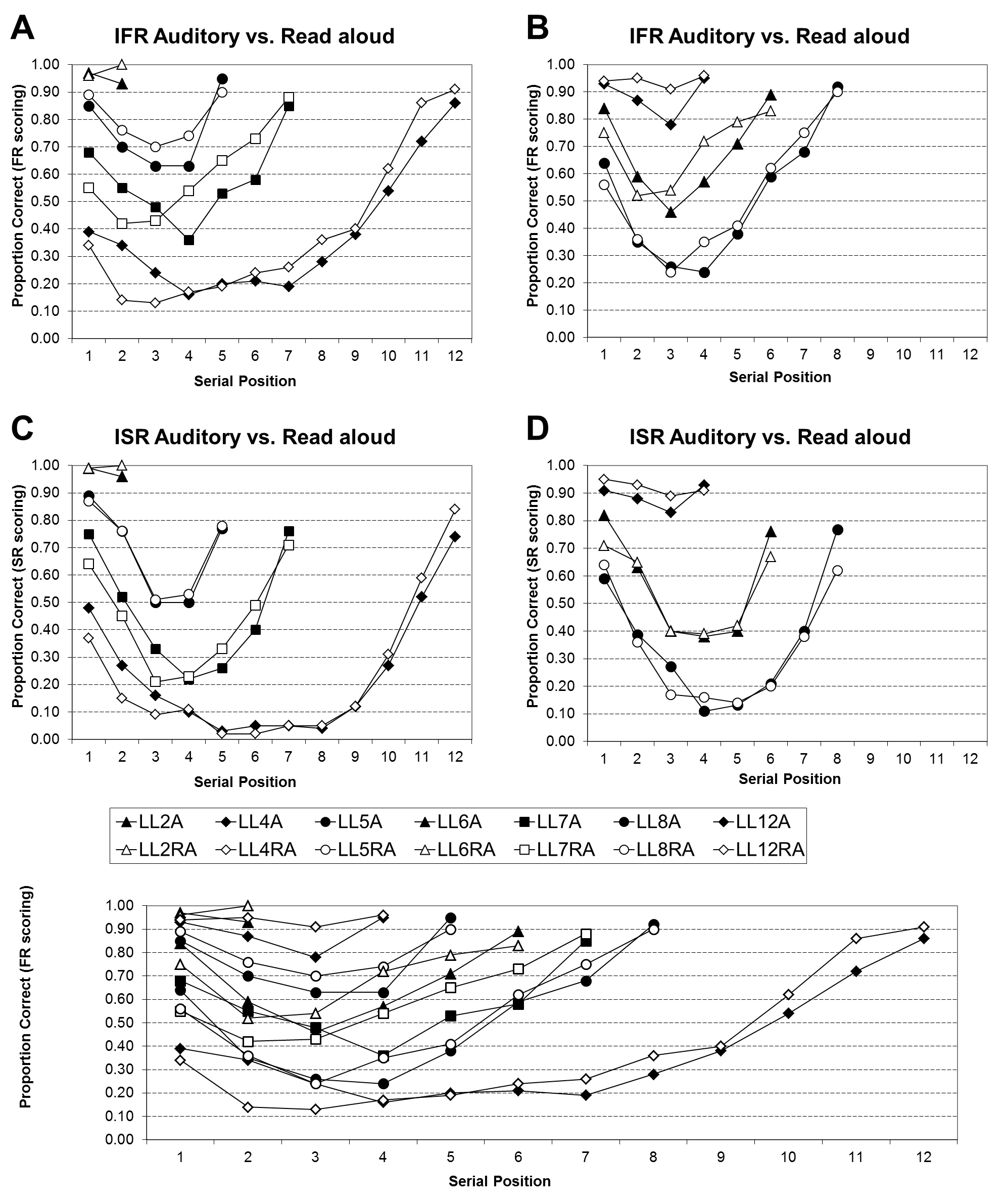


**Figure D1**. Overall serial position curves for the auditory and read aloud IFR conditions (using FR soring) and ISR conditions (using SR scoring).


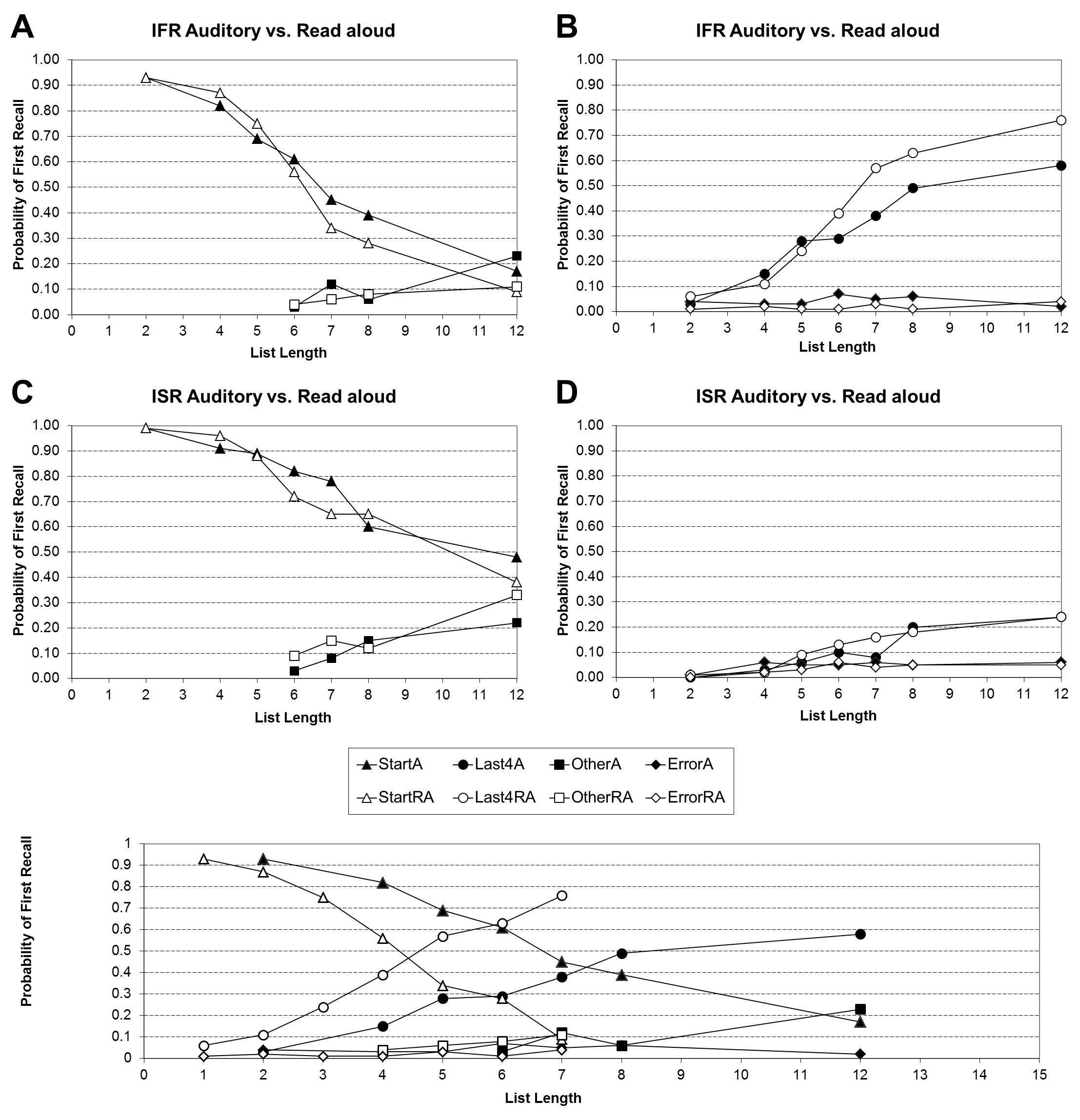


**Figure D2**. Probability of first recall (PFR) data for the auditory and read aloud conditions.


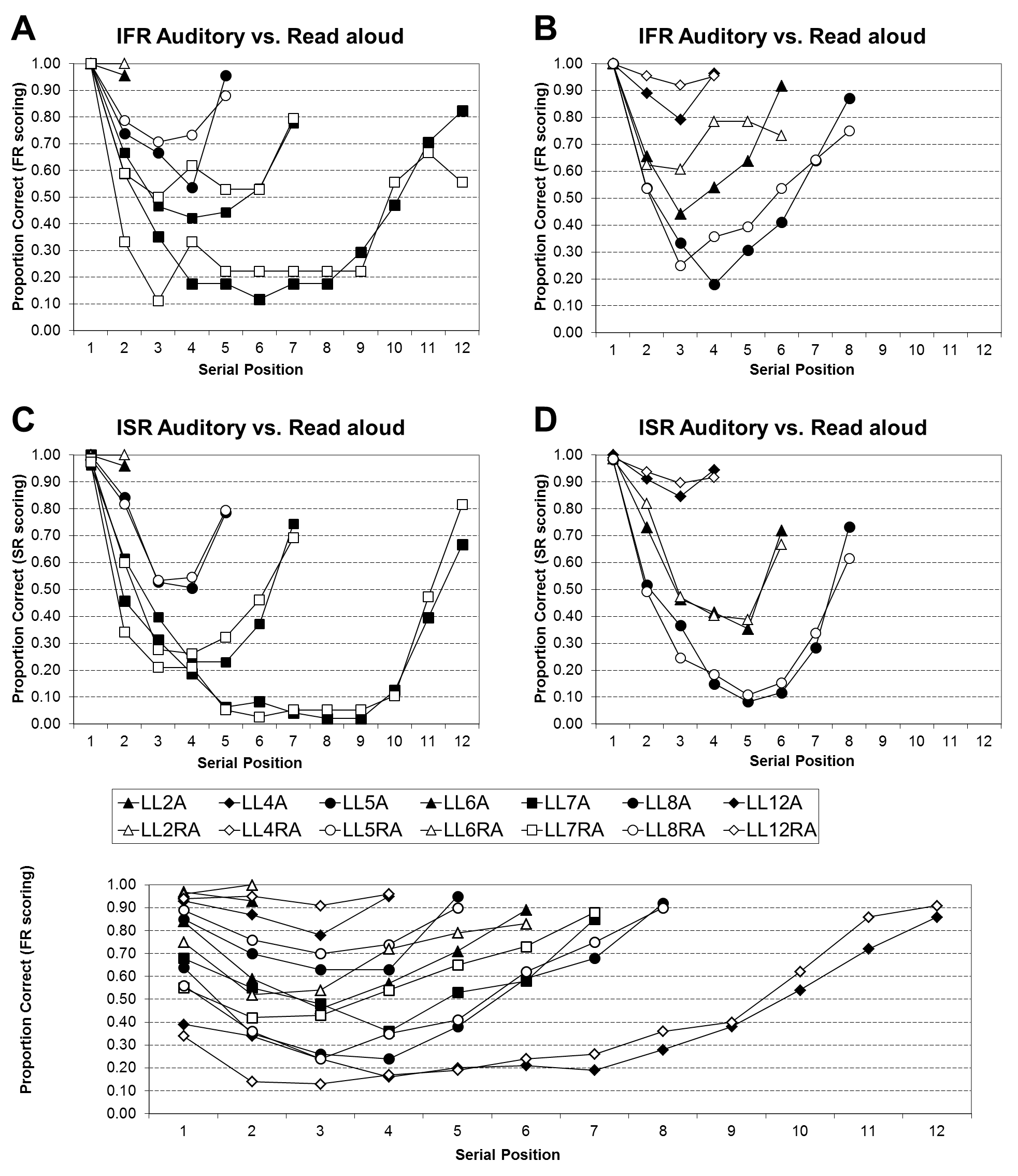


**Figure D3**. Resultant serial position curves for trials that began with SP1 for the auditory and read aloud IFR conditions (using FR soring) and ISR conditions (using SR scoring).

**
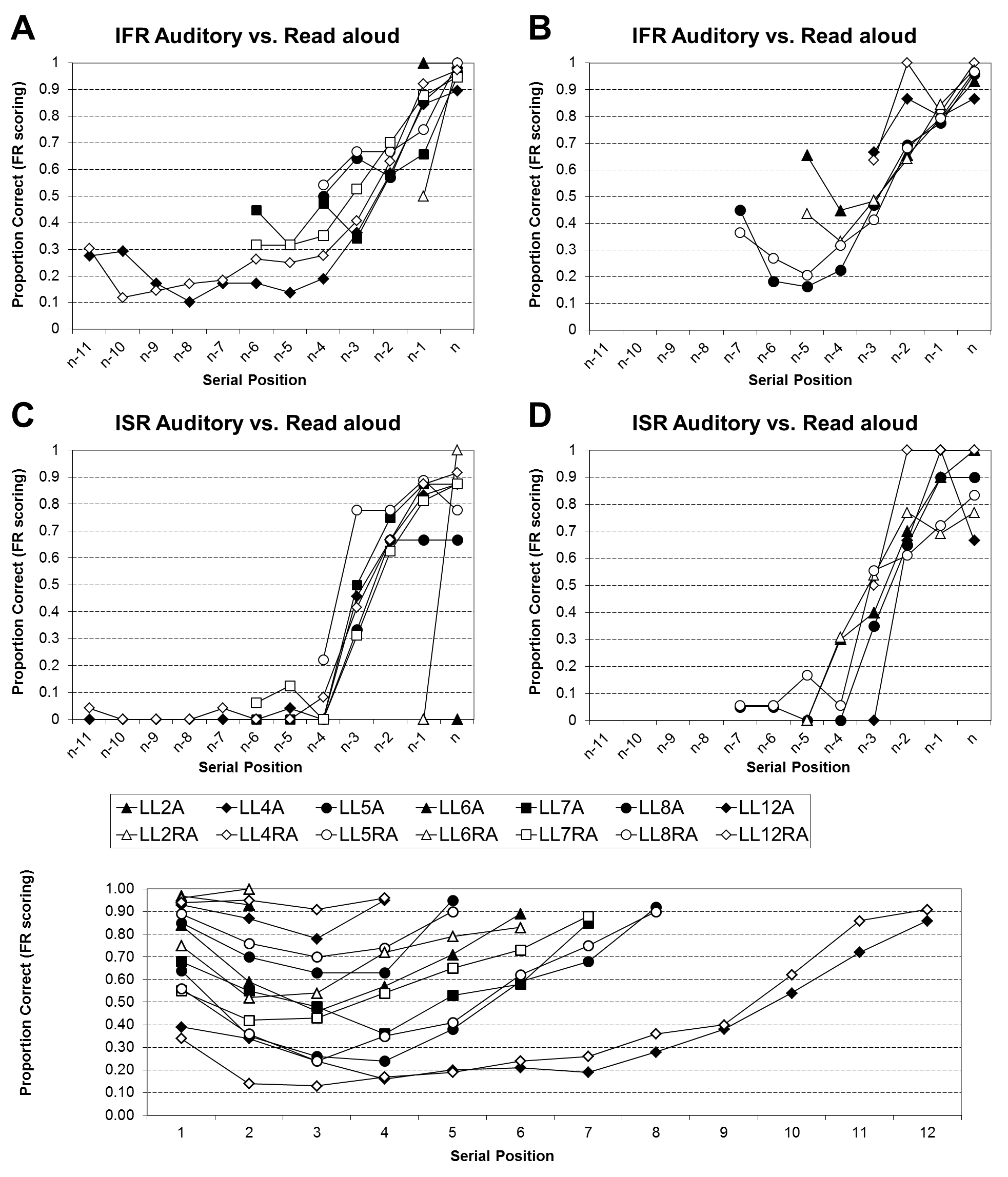
**

**Figure D4.** Resultant serial position curves for trials that began with any one of the last four words for the auditory and read aloud IFR conditions and ISR conditions (using FR scoring).
